# XCP-D: A robust pipeline for the post-processing of fMRI data

**DOI:** 10.1162/imag_a_00257

**Published:** 2024-08-13

**Authors:** Kahini Mehta, Taylor Salo, Thomas J. Madison, Azeez Adebimpe, Danielle S. Bassett, Max Bertolero, Matthew Cieslak, Sydney Covitz, Audrey Houghton, Arielle S. Keller, Jacob T. Lundquist, Audrey Luo, Oscar Miranda-Dominguez, Steve M. Nelson, Golia Shafiei, Sheila Shanmugan, Russell T. Shinohara, Christopher D. Smyser, Valerie J. Sydnor, Kimberly B. Weldon, Eric Feczko, Damien A. Fair, Theodore D. Satterthwaite

**Affiliations:** Lifespan Informatics and Neuroimaging Center (PennLINC), Department of Psychiatry, Perelman School of Medicine, University of Pennsylvania, Philadelphia, PA, United States; Penn/CHOP Lifespan Brain Institute, Perelman School of Medicine, Children’s Hospital of Philadelphia Research Institute, Philadelphia, PA, United States; Department of Psychiatry, Perelman School of Medicine, University of Pennsylvania, Philadelphia, PA, United States; Masonic Institute for the Developing Brain, University of Minnesota Medical School, Minneapolis, MN, United States; Department of Bioengineering, School of Engineering and Applied Science, University of Pennsylvania, Philadelphia, PA, United States; Department of Electrical and Systems Engineering, University of Pennsylvania, Philadelphia, PA, United States; Department of Neurology, University of Pennsylvania, Philadelphia, PA, United States; Department of Physics and Astronomy, University of Pennsylvania, Philadelphia, PA, United States; Santa Fe Institute, Santa Fe, NM, United States; Department of Pediatrics, University of Minnesota, Minneapolis, MN, United States; Penn Statistics in Imaging and Visualization Center, Department of Biostatistics, Epidemiology, and Informatics, Perelman School of Medicine, University of Pennsylvania, Philadelphia, PA, United States; Center for Biomedical Image Computing and Analytics, Department of Radiology, Perelman School of Medicine, University of Pennsylvania, Philadelphia, PA, United States; Departments of Neurology, Pediatrics, and Radiology, Washington University School of Medicine, St. Louis, MO, United States; Institute of Child Development, University of Minnesota Medical School, Minneapolis, MN, United States

**Keywords:** fMRI, software, post-processing, functional connectivity, resting-state

## Abstract

Functional neuroimaging is an essential tool for neuroscience research. Pre-processing pipelines produce standardized, minimally pre-processed data to support a range of potential analyses. However, post-processing is not similarly standardized. While several options for post-processing exist, they may not support output from different pre-processing pipelines, may have limited documentation, and may not follow generally accepted data organization standards (e.g., Brain Imaging Data Structure (BIDS)). In response, we present XCP-D: a collaborative effort between PennLINC at the University of Pennsylvania and the DCAN lab at the University of Minnesota. XCP-D uses an open development model on GitHub and incorporates continuous integration testing; it is distributed as a Docker container or Apptainer image. XCP-D generates denoised BOLD images and functional derivatives from resting-state data in either NIfTI or CIFTI files following pre-processing with fMRIPrep, HCP, or ABCD-BIDS pipelines. Even prior to its official release, XCP-D has been downloaded >5,000 times from DockerHub. Together, XCP-D facilitates robust, scalable, and reproducible post-processing of fMRI data.

## 1 Introduction

Functional neuroimaging using fMRI is an essential tool for human neuroscience research. Widely used pre-processing pipelines, including the fMRIPrep (Esteban et al., 2019), Human Connectome Project (HCP) (Glasser et al., 2013), and Adolescent Brain Cognitive Development^®^—Brain Imaging Data Structure (ABCD-BIDS) (Feczko et al., 2021) methods, produce standardized, minimally pre-processed data to support a range of potential analyses. We define pre-processing as it is designated by fMRIPrep (Esteban et al., 2019). This consists of signal corrections, spatiotemporal filtering, resampling onto a target space appropriate for analysis, and the extraction of confounds. Post-processing typically includes critical steps like denoising and generation of derived measures (e.g., functional networks) that are used in hypothesis testing; it does not include across-participant statistical analysis. Unlike the highly standardized software available for pre-processing, there is far greater variability in how researchers approach post-processing, for example censoring data to remove high-motion outliers or despiking data to remove large spikes in images. In general, different approaches towards denoising in post-processing can lead to different results from the same set of data (Li et al., 2022). Prior work has also established that denoising strategies are highly heterogeneous in their effectiveness (Ciric et al., 2017). This may result in findings that cannot be replicated and contradictory results, making it harder for the field to progress. Here, we introduce XCP-D: a scalable, robust, and generalizable software package for post-processing resting-state fMRI data.

Widely used pre-processing tools such as fMRIPrep build on the Brain Imaging Data Structure (BIDS) for organizing and describing neuroimaging data (K. J. Gorgolewski et al., 2016). As a BIDS App, fMRIPrep builds appropriate pre-processing workflows based on the metadata encoded by BIDS. Following pre-processing with fMRIPrep, many labs use custom workflows for post-processing steps, including denoising and generation of derivatives. While such a bespoke approach to analysis may have advantages—such as being tightly aligned with the needs of a specific study—it leads to the duplication of effort across labs, negatively impacts reproducibility, and may reduce the generalizability of results (Botvinik-Nezer et al., 2020). One alternative to custom post-processing has been provided by the eXtensible Connectivity Pipelines Engine (XCP;Ciric et al., 2018), a broadly adopted (>6,000 Docker pulls) post-processing package that consumes fMRIPrep output. However, XCP has accumulated substantial technical debt over time, is not compatible with other widely used pre-processing formats (e.g., HCP pipelines), does not support surface-based analyses, and lacks certain advanced denoising features provided by other widely used packages such as ABCD-BIDS (Feczko et al., 2021)—a widely used BIDS app from the DCAN that combines both the pre-processing and the post-processing stages (Developmental Cognition and Neuroimaging) lab (>10,000 Docker pulls).

Here, we introduce XCP-D, a collaborative effort between PennLINC (Pennsylvania Lifespan Informatics and Neuroimaging Center) and DCAN lab that includes a new Python codebase and important new features. XCP-D focuses on consuming data pre-processed by other widely used tools. Specifically, XCP-D supports post-processing of multiple pre-processed formats, including the fMRIPrep (Esteban et al., 2019), HCP (Glasser et al., 2013), and ABCD-BIDS (Feczko et al., 2021) methods; this allows XCP-D users to apply the same top-performing denoising strategies to datasets that were pre-processed using different software. The software has been tested extensively on data from children, adolescents, and adults; extensive ongoing development has extended this to infant data via integration with infant-fMRIPrep (previously NiBabies;Goncalves et al., 2023). Throughout, XCP-D adheres to field-standard conventions for data organization specified by BIDS (K. J. Gorgolewski et al., 2016). XCP-D also includes multiple software engineering features to ensure stability and robustness, such as a refactored and highly modular codebase that is built using NiPype (K. Gorgolewski et al., 2011) and incorporation of extensive continuous integration (CI) testing. Additionally, XCP-D now supports CIFTI workflows for surface-based analysis and processing, provides an expanded suite of data quality measures, and includes new visual reports. XCP-D also uses an open, test-driven development model on GitHub, and is distributed as a Docker (Rad et al., 2017) container or Apptainer (previously known as Singularity) image (Kurtzer et al., 2017). XCP-D thus allows users to leverage minimally processed data from diverse data resources, apply uniform post-processing, and generate the same derived measures for hypothesis testing. Prior to publication, XCP-D has already been pulled from DockerHub over 5,000 times.

## 2 Methods

### 2.1 Overview

XCP-D consumes pre-processed resting-state data generated with any of three commonly used pre-processing pipelines and implements top-performing denoising strategies (Ciric et al., 2018) for data in NIfTI or CIFTI format. The software generates resting-state derivatives, including parcellated timeseries and connectivity matrices in multiple popular atlases. XCP-D works on data either in volumetric (i.e., NIfTI) or hybrid surface-volumetric (i.e., CIFTI) formats. Importantly, XCP-D also calculates additional quality assurance measures. XCP-D also produces detailed error logs in the case of failure that can be accessed in each subject’s outputs. In addition, each derivative file’s metadata points to the files used to create it. XCP-D follows the BIDS-Derivatives specification, and aligns with current versions of BIDS Extension Proposals (BEPs) 11, 12, 17, and 38 in the latest version. Finally, XCP-D constructs interactive reports that describe the post-processing methods used and facilitate visualization of each step.

### 2.2 Software design and testing

We developed XCP-D using an open-source, test-driven approach. All source code is publicly available on GitHub to ensure transparency throughout the development cycle. Further, a large majority of the codebase is tested using CI tests via CircleCI to ensure that each module performs as expected prior to the release of new versions. At present, approximately 83% of XCP-D code is covered by our CI tests according to CodeCov, which reports both line and branch coverage. Furthermore, we applied branch protection rules during the development process: all changes to the XCP-D codebase must be approved by a reviewer and then pass CI testing before the updated code is released. These tests are described in detail inSupplemental Table 1.

### 2.3 Installation procedures

#### 2.3.1 Docker

Docker is an open-source platform for developers that makes the distribution of applications easier via the packaging of all supporting dependencies into a lightweight, standard form called a “container” (Rad et al., 2017). Docker images create a container that includes the complete operating system and all necessary dependencies. For every new version of XCP-D, CI testing is performed. If these tests succeed, a new Docker image is automatically generated and deployed to DockerHub (Rad et al., 2017). To run XCP-D via Docker images, Docker Engine must be installed. To pull XCP-D from DockerHub, users must run:

docker pull pennlinc/xcp_d:<version>

where <version> should be replaced with the desired version or tag of XCP-D that users want to download. The image can also be found here:https://hub.docker.com/r/pennlinc/xcp_d.

XCP-D can be run by interacting directly with the Docker Engine via the docker run command.

#### 2.3.2 Apptainer

Apptainer is an open-source software package designed to allow portable computational environments and containers for scientific research (Kurtzer et al., 2017). Many high performance computing (HPC) systems restrict use of Docker (Rad et al., 2017), but support Apptainer instead. Users can create an Apptainer image from a Docker image on DockerHub:

singularity build xcp_d–<version>.sif docker://pennlinc/xcp_d:<version>

### 2.4 Workflow

Post-processing in XCP-D involves multiple customizable steps that are widely used: the removal of dummy volumes, despiking, interpolation, filtering, regression, temporal censoring, smoothing, the calculation of quality assurance variables, and generation of reports (Ciric et al., 2018;Lindquist et al., 2019;Satterthwaite et al., 2013; seeFig. 1). Note that XCP-D supports post-processing of fMRI data with a T1 image, a T2 image, or both. Outputs are in CIFTI, NIfTI, TSV, JSON, HTML and in the case of the DCAN QC file, HDF5.

**Fig. 1.**
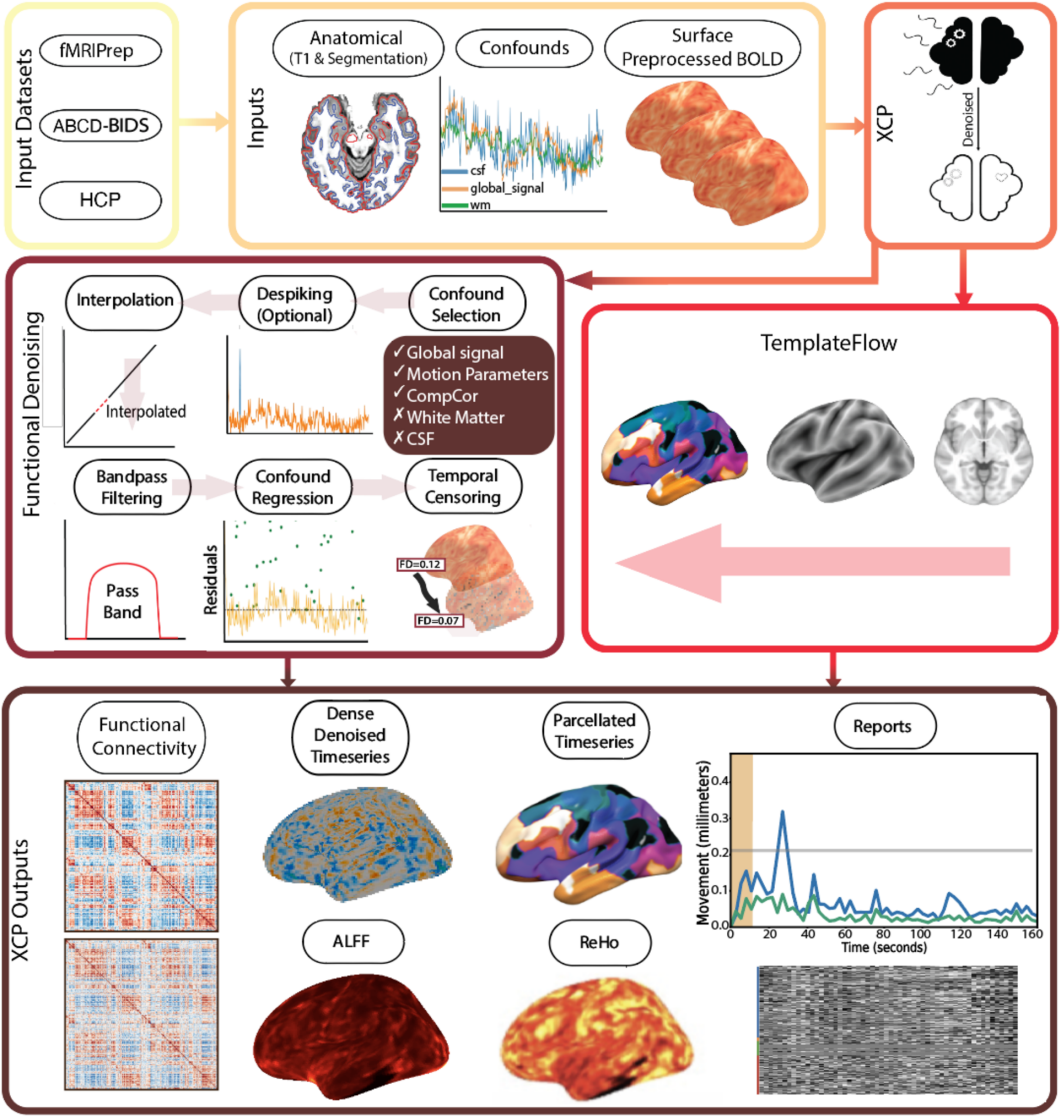
XCP-D Workflow. The XCP-D workflow begins after the pre-processing of fMRI data. XCP-D requires anatomical data, confounds files, and pre-processed BOLD files. It performs functional denoising to produce clean fMRI data and functional derivatives. ReHo: Regional Homogeneity; ALFF: Amplitude of Low Frequency Fluctuations.

Through these processes, XCP-D produces multiple functional derivatives, including the dense (non-parcellated, vertex, or voxel-wise data) volumetric, or surface-based (stored in “dtseries.nii”), denoised timeseries, parcellated timeseries (stored in “ptseries.nii” for CIFTIs), functional connectivity matrices, and derived functional metric maps (such as regional homogeneity and amplitude of low-frequency fluctuations). Furthermore, XCP-D also provides detailed quality assurance information regarding both the fMRI data and image registration, as well as graphical reports (seeTable 1for a list and description of XCP-D outputs).

**Table 1.** XCP-D outputs.

File name	File type	Description
xcp_d/sub–<label> [_ses–<label>]_executive_summary.html	Report	Executive summary per session
xcp_d/sub–<label>.html	Report	NiPreps summary per participant
xcp_d/atlases/atlas–<label>/atlas–<label>_dseg.tsv	Atlases	Label information for the atlas used
xcp_d/atlases/atlas–<label>/atlas–<label>_space–<label>_dseg.nii.gz or atlas–<label>_space– <label>_dseg.dlabel.nii	Atlases	Atlas in NIfTI or CIFTI format
xcp_d/sub–<label>/[ses–<label>/]anat/<source_entities>_space–<label>_desc–preproc_T1w.nii.gz	Anatomical	Pre-processed T1w in MNI space
xcp_d/sub–<label>/[ses–<label>/]anat/<source_entities>_space–<label>_desc–preproc_T2w.nii.gz	Anatomical	Pre-processed T2w in MNI space
xcp_d/sub–<label>/[ses–<label>/]anat/<source_entities>_space–<label>_dseg.nii.gz	Anatomical	Pre-processed .dseg in MNI space
xcp_d/sub–<label>/[ses–<label>/]anat/<source_entities>_space–fsLR_den–32k_hemi–<L|R>_desc–hcp_midthickness.surf.gii	Anatomical	Reconstructed surfaces warped to fsLR space at 32 k density
xcp_d/sub–<label>/[ses–<label>/]anat/<source_entities>_space–fsLR_den–32k_hemi–<L|R>_desc–hcp_inflated.surf.gii	Anatomical	Reconstructed surfaces warped to fsLR space at 32 k density
xcp_d/sub–<label>/[ses–<label>/]anat/<source_entities>_space–fsLR_den–32k_hemi–<L|R>_desc–hcp_vinflated.surf.gii	Anatomical	Reconstructed surfaces warped to fsLR space at 32 k density
xcp_d/sub–<label>/[ses–<label>/]anat/<source_entities>_space–fsLR_den–32k_hemi–<L|R>_desc–pial.surf.gii	Anatomical	Reconstructed surfaces warped to fsLR space at 32 k density
xcp_d/sub–<label>/[ses–<label>/]anat/<source_entities>_space–fsLR_den–32k_hemi–<L|R>_desc–smoothwm.surf.gii	Anatomical	Reconstructed surfaces warped to fsLR space at 32 k density
xcp_d/sub–<label>/[ses–<label>/]anat/<source_entities>_space–fsLR_den–32k_hemi–<L|R>_sulc.shape.gii	Anatomical	Sulcal depth in fsLR space at 32 k density
xcp_d/sub–<label>/[ses–<label>/]anat/<source_entities>_space–fsLR_den–32k_hemi–<L|R>_curv.shape.gii	Anatomical	Sulcal curvature in fsLR space at 32 k density
xcp_d/sub–<label>/[ses–<label>/]anat/<source_entities>_space–fsLR_den–32k_hemi–<L|R>_thickness.shape.gii	Anatomical	Cortical thickness in fsLR space at 32 k density
xcp_d/sub–<label>/[ses–<label>/]anat/<source_entities>_space–fsLR_seg–<label>_den–32k_stat–mean_desc–sulc_morph.tsv	Anatomical	Parcellated sulcal depth estimates
xcp_d/sub–<label>/[ses–<label>/]anat/<source_entities>_space–fsLR_seg–<label>_den–32k_stat–mean_desc–curv_morph.tsv	Anatomical	Parcellated sulcal curvature estimates
xcp_d/sub–<label>/[ses–<label>/]anat/<source_entities>_space–fsLR_seg–<label>_den–32k_stat–mean_desc–thickness_morph.tsv	Anatomical	Parcellated cortical thickness estimates
xcp_d/sub–<label>/[ses–<label>/]func/<source_entities>_space–<label>_desc–denoised_bold.nii.gz or <source_entities>_space–fsLR_den–91k_desc–denoised_bold.dtseries.nii	Functional	Denoised BOLD file
xcp_d/sub–<label>/[ses–<label>/]func/<source_entities>_space–<label>_desc–denoisedSmoothed_bold.nii.gz or <source_entities>_space–fsLR_den–91k_desc–denoisedSmoothed_bold.dtseries.nii	Functional	Smoothed, denoised BOLD file
xcp_d/sub–<label>/[ses–<label>/]func/<source_entities>_space–<label>_desc–interpolated_bold.nii.gz or <source_entities>_space–fsLR_den–91k_desc–interpolated_bold.dtseries.nii	Functional	Interpolated BOLD file
xcp_d/sub–<label>/[ses–<label>/]func/<source_entities>_space–<label>_seg–<label>_stat–coverage_bold.tsv or <source_entities>_space–fsLR_seg–<label>_den–91k_stat–coverage_bold.tsv and <source_entities>_space–fsLR_seg–<label>_den–91k_stat–coverage_boldmap.pscalar.nii	Functional	Coverage information. Produced in .tsv and .pscalar format for CIFTIs
xcp_d/sub–<label>/[ses–<label>/]func/<source_entities>_space–<label>_seg–<label>_stat–mean_timeseries.tsv or <source_entities>_space–fsLR_seg–<label>_den–91k_tstat–mean_timeseries.tsv and <source_entities>_space–fsLR_seg–<label>_den–91k_stat–mean_timeseries.ptseries.nii	Functional	Mean timeseries for functional data, after atlas parcellation. Produced in .tsv and .ptseries format for CIFTIs
xcp_d/sub–<label>/[ses–<label>/]func/<source_entities>_space–<label>_seg–<label>_stat–pearsoncorrelation_relmat.tsv or <source_entities>_space–fsLR_seg–<label>_den–91k_stat–pearsoncorrelation_relmat.tsv and <source_entities>_space–fsLR_seg–<label>_den–91k_stat–pearsoncorrelation_boldmap.pconn.nii	Functional	Connectivity matrix for functional data, after atlas parcellation. Produced in .tsv and .pconn format for CIFTIs
xcp_d/sub–<label>/[ses–<label>/]func/<source_entities>_space–<label>_seg–<label>_stat–pearsoncorrelation_desc–<INT> volumes_relmat.tsv or <source_entities>_space–fsLR_seg–<label>_den–91k_stat–pearsoncorrelation_desc–<INT> volumes_relmat.tsv	Functional	Connectivity matrix for functional data, after atlas parcellation. Correlation matrices with the desc-<INT>volumes entity are produced if the– –exact-time parameter is used.
xcp_d/sub–<label>/[ses–<label>/]func/<source_entities>_space–<label>_stat–reho_boldmap.nii.gz or <source_entities>_space–fsLR_den–91k_stat–reho_boldmap.dscalar.nii	Functional	ReHo image for BOLD data
xcp_d/sub–<label>/[ses–<label>/]func/<source_entities>_space–<label>_seg–<label>_stat–reho_bold.tsv or <source_entities>_space–fsLR_seg–<label>_stat–reho_bold.tsv	Functional	Parcellated ReHo image
xcp_d/sub–<label>/[ses–<label>/]func/<source_entities>_space–<label>_stat–alff_boldmap.nii.gz or <source_entities>_space–fsLR_den–91k_stat–alff_boldmap.dscalar.nii	Functional	ALFF image for BOLD data
Xcp_d/sub–<label>/[ses–<label>/]func/<source_entities>_space–<label>_stat–alff_desc–smooth_boldmap.nii.gz or <source_entities>_space–fsLR_den–91k_stat– alff_desc–smooth_boldmap.dscalar.nii	Functional	Smoothed ALFF image
xcp_d/sub–<label>/[ses–<label>/]func/<source_entities>_space–<source_entities>_space–<label>_seg–<label>_stat–alff_bold.tsv or <source_entities>_space–fsLR_seg–<label>_stat–alff_bold.tsv	Functional	Parcellated ALFF image
xcp_d/sub–<label>/[ses–<label>/]func/<source_entities>_space–<label>_desc–linc_qc.tsv or <source_entities>_space–fsLR_desc–linc_qc.tsv	Quality check	Quality control metrics, including motion and registration information
xcp_d/sub–<label>/[ses–<label>/]func/<source_entities>[_desc–filtered]_motion.tsv	Quality check	A tab-delimited file with seven columns: one for each of the six filtered motion parameters, as well as “framewise_displacement”. If no motion filtering was applied, this file will not have the desc entity. This file includes the high-motion volumes that are removed in most other derivatives.
xcp_d/sub–<label>/[ses–<label>/]func/<source_entities>_outliers.tsv	Quality check	A tab-delimited file with one column: “framewise_displacement”. The “framewise_displacement” column contains zeros for low-motion volumes, and ones for high-motion outliers. This file includes the high-motion volumes that are removed in most other derivatives.
xcp_d/sub–<label>/[ses–<label>/]func/<source_entities>_design.tsv	Quality check	A tab-delimited file with one column for each nuisance regressor, including an intercept column, a linear trend column, and one-hot encoded regressors indicating each of the high-motion outlier volumes. This file includes the high-motion volumes that are removed in most other derivatives.
xcp_d/sub–<label>/[ses–<label>/]func/<source_entities>_desc–dcan_qc.hdf5	DCAN-style quality check	This file is in .hdf5 format (readable by h5py), and contains binary censoring masks from 0.0 to 1 mm FD in 0.01 steps. This file contains: **FD_threshold** : a number ≥ 0 that represents the FD threshold used to calculate the metrics in this list **frame_removal** : a binary vector/array the same length as the number of frames in the concatenated timeseries, indicates whether a frame is removed (1) or not (0). **format_string** (legacy): a string that denotes how the frames were excluded **total_frame_count** : a whole number that represents the total number of frames in the concatenated series **remaining_frame_count** : a whole number that represents the number of remaining frames in the concatenated series **remaining_seconds** : a whole number that represents the amount of time remaining after thresholding **remaining_frame_mean_FD** : a number ≥ 0 that represents the mean FD of the remaining frames

This table describes outputs from a run of XCP-D.

Many internal operations of the software use TemplateFlow (Ciric et al., 2022), Nibabel (Brett et al., 2023), numpy (Harris et al., 2020), and scipy (Virtanen et al., 2020). Below, we describe each of the post-processing modules with accompanying command syntax, relevant information, as well as the CI tests for each module. We also describe a list of command-line options inTable 2.

**Table 2.** Command-line options for an XCP-D run.

Option	Type	Description	Default	Optional
fmri_dir	Positional argument	The root folder of a pre-processed fMRI output	None	No
output_dir	Positional argument	The output path for XCP-D	None	No
analysis_level	Positional argument	The analysis level for XCP-D, must be specified as participant	None	No
– –version	Named argument	Show program’s version number and exit	None	Yes
– –participant_label,– –participant–label	Options for filtering BIDS queries	A space delimited list of participant identifiers or a single identifier (the sub- prefix can be removed)	None	Yes
–t, – –task–id, – –task_id	Options for filtering BIDS queries	Select a specific task to be selected for the post-processing (users can only specify one at a time)	None	Yes
– –bids–filter–file, – –bids–filter–file	Options for filtering BIDS queries	A .JSON file defining BIDS input filters. XCP-D allows users to choose which pre-processed files will be post-processed with the – –bids–filter–file parameter. This argument must point to a .JSON file, containing filters that will be fed into PyBIDS.The keys in this .JSON file are unique to XCP-D. They are our internal terms for different inputs that will be selected from the pre-processed dataset. “bold” determines which pre-processed BOLD files will be chosen. You can set a number of entities here, including session, task, space, resolution, and density.	None	Yes
–s, – –cifti	Options for CIFTI processing	Post-process CIFTI instead of NIfTI—this is set to true automatically for HCP and DCAN input types	False	Yes
– –nprocs, – –nthreads,– –n–cpus, – –n_cpus	Options for resource management	Maximum number of threads across all processes	2	Yes
– –omp–nthreads, – –omp_nthreads	Options for resource management	Maximum number of threads per process	1	Yes
– –mem–gb, – –mem_gb	Options for resource management	Upper bound memory limit for XCP-D processes	None	Yes
– –low–mem, – –low_mem	Options for resource management	Attempt to reduce memory usage (will increase disk usage in working directory).	False	Yes
– –use–plugin, – –use_plugin, – –nipype–plugin–file, – –nipype_plugin_file	Options for resource management	Nipype plugin configuration file. For more information, see https://nipype.readthedocs.io/en/0.11.0/users/plugins.html .	None	Yes
–v, – –verbose	Options for resource management	Increases log verbosity for each occurrence; debug level is -vvv	0	Yes
– –input–type, – –input_type	Input flag	The pipeline used to generate the pre-processed derivatives. The default option is fmriprep. The hcp and dcan options are also supported.	fmriprep	Yes
– –smoothing	Parameters for post-processing	FWHM, in millimeters, of the Gaussian smoothing kernel to apply to the denoised BOLD data. This may be set to 0.	6	Yes
– –despike	Parameters for post-processing	Despike the NIfTI/CIFTI before processing	False	Yes
–p, – –nuisance–regressors, – –nuisance_regressors	Parameters for post-processing	Nuisance parameters to be selected. See Ciric et al. (2017) .Possible choices: 27P, 36P, 24P, acompcor, aroma, acompcor_gsr, aroma_gsr, custom, none, gsr_only	36P	Yes
– –dummy–scans, – –dummy_scans	Parameters for post-processing	Number of volumes to remove from the beginning of each run. If set to auto, xcp_d will extract non-steady-state volume indices from the preprocessing derivatives’ confounds file.	0	Yes
–m, –combineruns	Parameters for post-processing	This option concatenates derivatives across runs, for each task separately	False	Yes
–c, – –custom_confounds, – –custom–confounds	Parameters for post-processing	Custom confounds to be added to nuisance regressors. Must be a folder containing confounds files, in which case the file with the name matching the pre-processing confounds file will be selected.	None	Yes
– –motion–filter–type, – –motion_filter_type	Motion filtering parameters	Type of band-stop filter to use for removing respiratory artifact from motion regressors. If not set, no filter will be applied. Possible choices: lp, notch. If the filter type is set to notch, then both band–stop–min and band–stop–max must be defined. If the filter type is set to lp, then only band–stop–min must be defined.	None	Yes
– –band–stop–min, – –band_stop_min	Motion filtering parameters	Lower frequency for the band-stop motion filter, in breaths-per-minute (bpm). Motion filtering is only performed if motion–filter–type is defined by the user. If used with the lp motion-filter-type, this parameter essentially corresponds to a low-pass filter (the maximum allowed frequency in the filtered data). This parameter is used in conjunction with motion–filter–order and band–stop–max. When motion–filter–type is set to lp (low-pass filter), another commonly-used value for this parameter is 6 BPM (equivalent to 0.1 Hertz.)	None	Yes
– –band–stop–max, – –band_stop_max	Motion filtering parameters	Upper frequency for the band-stop motion filter, in breaths-per-minute (bpm). Motion filtering is only performed if motion–filter–type is defined by the user. This parameter is only used if motion–filter–type is set to notch. This parameter is used in conjunction with motion–filter–order and band–stop–min.	None	Yes
– –motion–filter–order, – –motion_filter_order	Motion filtering parameters	Number of filter coefficients for the band-stop filter	4	Yes
–r, – –head_radius, – –head–radius	Censoring options f	Head radius for computing FD. The default is 50 mm, but 35 mm is recommended for infants. A value of auto is also supported, in which case the brain radius is estimated from the pre-processed brain mask by treating the mask as a sphere	50	Yes
–f, – –fd–thresh, – –fd_thresh	Censoring options	Framewise displacement threshold for censoring. A threshold of 0 will disable censoring.	0.3	Yes
–min–time, – –min_time	Censoring options	Post-scrubbing threshold to apply to individual runs in the dataset. This threshold determines the minimum amount of time, in seconds, needed to post-process a given run, once high-motion outlier volumes are removed. This will have no impact if scrubbing is disabled (i.e., if the FD threshold is zero or negative). This parameter can be disabled by providing a zero or a negative value.	240	Yes
– –disable–bandpass–filter, – –disable_bandpass_filter	Data filtering options	Disable band-pass filtering. If band-pass filtering is disabled, then ALFF derivatives will not be calculated.	Yes	Yes
– –lower–bpf, – –lower_bpf	Data filtering options	Lower cut-off frequency (Hz) for the Butterworth band-pass filter to be applied to the denoised BOLD data. Set to 0.0 or negative to disable high-pass filtering. See Satterthwaite et al. (2013) .	0.01	Yes
– –upper–bpf, – –upper_bpf	Data filtering options	Upper cut-off frequency (Hz) for the Butterworth band-pass filter to be applied to the denoised BOLD data. Set to 0.0 or negative to disable low-pass filtering. See Satterthwaite et al. (2013) .	0.08	Yes
– –bpf–order, – –bpf_order	Data filtering options	Number of filter coefficients for the Butterworth band-pass filter.	2	Yes
– –atlases	Parcellation options	4S1056Parcels, 4S156Parcels, 4S256Parcels, 4S356Parcels, 4S456Parcels, 4S556Parcels, 4S656Parcels, 4S756Parcels, 4S856Parcels, 4S956Parcels, Glasser, Gordon, HCP, TianSelection of atlases to apply to the data. All are used by default.	All	Yes
– –skip–parcellation, – –skip_parcellation	Parcellation options	Skip parcellation and correlation	False	Yes
– –min–coverage, – –min_coverage	Parcellation options	Coverage threshold to apply to parcels in each atlas. Any parcels with lower coverage than the threshold will be replaced with NaNs. Must be a value between zero and one, indicating proportion of the parcel.	0.5	Yes
– –exact–time, – –exact_time	Parcellation options	If used, this parameter will produce correlation matrices limited to each requested amount of time. If there is more than the required amount of low-motion data, then volumes will be randomly selected to produce denoised outputs with the exact amounts of time requested. If there is less than the required amount of “good” data, then the corresponding correlation matrix will not be produced.	None	Yes
– –random–seed, – –random_seed	Other options	Initialize the random seed for the workflow.	None	Yes
–w, – –work_dir, – –work–dir	Other options	Path where intermediate results should be stored	working_dir	Yes
– –clean–workdir, – –clean_workdir	Other options	Clears working directory of contents. Use of this flag is not recommended when running concurrent processes of XCP-D.	False	Yes
– –resource–monitor, – –resource_monitor	Other options	Enable Nipype’s resource monitoring to keep track of memory and CPU usage	False	Yes
– –config–file, – –config_file	Other options	Use pre-generated configuration file. Values in file will be overridden by command-line arguments.	None	Yes
– –write–graph	Other options	Write workflow graph	False	Yes
– –stop–on–first–crash	Other options	Force stopping on first crash, even if a work directory was specified.	False	Yes
– –debug	Other options	Possible choices: pdb, allDebug mode(s) to enable. “all” is alias for all available modes.	None	Yes
– –md–only–boilerplate	Other options	Skip generation of HTML and LaTeX formatted citation with pandoc	False	Yes
– –boilerplate–only, – –boilerplate_only	Other options	Generate boilerplate only	False	Yes
– –reports–only	Other options	Only generate reports, do not run workflows. This will only rerun report aggregation, not reportlet generation for specific nodes.	False	Yes
– –notrack	Other options	Opt out of sending tracking information	False	Yes
– –fs–license–file	Other options	Path to FreeSurfer license key file. Get it (for free) by registering at https://surfer.nmr.mgh.harvard.edu/registration.html	None	Yes. Users can alternatively mount the license and set an environment variable.
– –warp–surfaces–native2std, – –warp_surfaces_native2std	Experimental options	If used, a workflow will be run to warp native-space (fsnative) reconstructed cortical surfaces (surf.gii files) produced by Freesurfer into standard (fsLR) space. These surface files are primarily used for visual quality assessment. By default, this workflow is disabled. IMPORTANT: This parameter can only be run if the – –cifti flag is also enabled.	False	Yes

### 2.5 Ingression of non-BIDS derivatives

When preprocessed data do not align with BIDS conventions (e.g., HCP), XCP-D indexes the outputs from the pre-processing pipeline and maps the relevant files into a BIDS derivatives-compliant structure in the working directory if the user specifies – –input–type hcp.

As part of this ingression procedure, XCP-D also extracts minimal confounds. However, this does not fully reproduce the confounds that fMRIPrep creates, which limits the denoising strategies available for these data (see*Confound selection*). Additionally, XCP-D’s anatomical workflow requires that CIFTI surfaces are in fsLR (Freesurfer templates;Van Essen et al., 2012) space at 32 k density.

### 2.6 Removal of non-steady state volumes

Some vendors acquire multiple additional volumes at the beginning of a scan to reduce transient T1 signals before a steady state is approached (Jenista et al., 2017). These volumes are often referred to as “dummy scans” or “non-steady state volumes.” This is the first post-processing step in XCP-D and occurs optionally. XCP-D allows the first*n*(as supplied by users) number of volumes to be deleted before processing. If set to auto, XCP-D will extract non-steady-state volume indices from the pre-processing derivatives confounds file (only included in fMRIPrep confounds files;Esteban et al., 2019). Removal of dummy volumes is enabled via the – –dummy–scans flag and feeds the truncated confounds and image files into the rest of the workflow.

### 2.7 Despiking

Despiking is a process in which large spikes in the BOLD timeseries are truncated on an adaptive, voxel-specific basis. Despiking limits the amplitude of the large spikes but preserves the data points with an imputed reduced amplitude to minimize the effect of outliers. Notably, despiking is different from temporal censoring (see*Temporal censoring*) as it modifies rather than deletes data—despiking is also performed individually for each voxel whereas temporal censoring removes an entire volume. XCP-D performs despiking via AFNI’s (Cox, 1996;Cox & Hyde, 1997) 3dDespike using default settings and the – –NEW flag, which uses a new fitting algorithm to despike the data rather than AFNI’s (Cox, 1996;Cox & Hyde, 1997) original L1 method, due to faster processing speed. For CIFTIs, which are first converted to NIfTIs and back during the despiking process via Connectome Workbench (Marcus et al., 2011), the – –nomask flag is used so that the entire volume is despiked. Despiking is performed when the – –despike flag is supplied. Despiking is executed before regression, censoring, and filtering to minimize the impact of spikes.

### 2.8 Filtering of realignment parameters

Recent work has established that respiration can systematically induce fluctuations in the main magnetic field (Fair et al., 2020), which can contaminate estimates of head motion. Such artifacts can be removed via filtering of the realignment parameters using a low-pass filter (Gratton et al., 2020) or a notch filter (Fair et al., 2020). If users specify a low-pass filter (as is recommended for single-band fMRI data), frequencies above band_stop_min (specified in breaths per minute) are removed from the motion parameters with a Butterworth filter. If users specify a notch filter (as is recommended for multi-band fMRI data, and described inFair et al., 2020), the frequencies between band_stop_min and band_stop_max are removed. The notch filter is applied using scipy’s (Virtanen et al., 2020) iirnotch function, and both filters are applied backwards and forwards using scipy’s (Virtanen et al., 2020) filtfilt function. Motion parameter filtering will only be enabled if – –motion–filter–type is provided.

### 2.9 Confound selection

Confound selection occurs when a confounds file is supplied from a pre-processing software. Regressors commonly present in these confounds files typically include realignment parameters, mean timeseries from anatomical compartments (GM or grey matter, WM or white matter, CSF or cerebrospinal fluid), the global signal (Fox et al., 2009), CompCor components (Behzadi et al., 2007), and/or independent components from ICA-AROMA (Pruim et al., 2015). Confound configurations can be extracted from these parameters and are then used to remove noise from the BOLD image file during confound regression. Confound configuration preferences may vary across use cases; thus, XCP-D allows users some flexibility in denoising options (Ciric et al., 2017;Satterthwaite et al., 2013).

The built-in nuisance strategies may be supplemented or replaced with a custom confounds file provided by the user. This functionality allows users to perform more advanced regression strategies. For example, users may convolve task regressors with a hemodynamic response function and provide these regressors in a custom confounds file to regress out task signals and treat the denoised data as pseudo-rest (Fair et al., 2007). If users wish to retain specific signals of interest in the data, they may include those signals in the custom confounds file, with the associated column headers prefixed with “signal__.” This scenario is described in*Confound regression*.

Confound selection is implemented via Nilearn’s ( Abraham et al., 2014 ) load_confound functionality. The selected confounds are fed into the beginning of the workflow in .tsv format where dummy time is removed—so it is appropriately truncated, and then passed on throughout the workflow. Pre-configured confound strategies include those described in a prior benchmarking study ( Ciric et al., 2018 ):

- 24P—six realignment parameters, their squares, derivatives, and squares of the derivatives
- 27P—the WM, CSF, and global signal parameters in addition to those included in the 24P model
- 36P (default)—the squares, derivatives, and squares of the derivatives of WM, CSF, and global signal parameters in addition to those included in the 27P model
- acompcor—the aCompCor parameters, the six realignment parameters, and their derivatives
- acompcor_gsr—the aCompCor parameters, the realignment parameters, their derivatives, and global signal
- aroma—the AROMA parameters, realignment parameters, their derivatives, WM, and CSF
- aroma_gsr—the AROMA parameters, realignment parameters, their derivatives, WM, CSF, and global signal
- gsr_only—global signal only
- Custom confounds—users provide their own confounds

Confound parameters can be selected by the user via the –p flag and corresponding configuration, or –c for custom confounds. Note that, at present, only 24P, 27P, 36P, and gsr_only are supported for HCP (Glasser et al., 2013) and ABCD-BIDS (Feczko et al., 2021) formats. Nuisance regressors can also be specified as none to skip this denoising step.

### 2.10 Interpolation

After optional despiking, the BOLD data and confounds are fed into the interpolation workflow, in which high-motion outlier volumes are replaced with interpolated data.

First, framewise displacement (FD) is calculated from the (optionally filtered) realignment parameters following the procedure described inPower et al. (2014). The head radius used to calculate FD may be supplied by the user via – –head–radius, set to auto (which estimates the brain radius based on the pre-processed brain mask), or by defaulting to 50 mm. The FD timeseries and FD threshold are then used to determine the number of high motion outlier volumes. A temporal mask is then generated in .tsv format, with 0 seconds corresponding to volumes that were not flagged for interpolation, and 1 second indicating high-motion outlier volumes. This mask is used to flag data that need to be interpolated in both the BOLD data and the confounds, producing a denoised timeseries which is the primary output of XCP-D.

The interpolation workflow uses cubic spline interpolation, as implemented in Nilearn (Abraham et al., 2014). The outlier volumes that are replaced are any volumes with FD greater than the value supplied by the user via – –fd–thresh (see*Temporal censoring*below). Any outlier volumes at the beginning or end of the run are replaced with the closest non-outlier volume’s data, to avoid inadvertent extrapolation by the interpolation function. The same interpolation is then applied to the confounds to ensure that all filtering processes applied to the fMRI data are reflected in the confounds, so as to avoid later reintroduction of variance removed by a previous step in the workflow (Lindquist et al., 2019).

Interpolation (and later censoring) can be disabled by setting – –fd–thresh to 0. Both the interpolated BOLD and confounds data are then fed into the filtering workflow.

### 2.11 Filtering

Temporal filtering is used in fMRI signal processing to reduce high-frequency and low-frequency artifacts in the timeseries. First, the interpolated BOLD data and confounds are detrended with a linear model. This step also mean-centers the BOLD data and confounds over time.

Then, XCP-D applies a Butterworth band-pass filter to the confounds and the BOLD signals. Functional connectivity between regions of interest is typically determined based on synchrony in low-frequency fluctuations (Biswal et al., 1995); therefore, removing higher frequencies using a low-pass filter may effectively remove noise from the timeseries while retaining signals of interest. High-pass filters can be used to remove very-low-frequency drift, which is a form of scanner noise, from an acquisition. Any frequencies below the low-pass cutoff and above the high-pass cutoff will be counted as pass-band frequencies as in the case of our Butterworth filter. These will be retained by the filter when it is applied. High-pass or low-pass only filtering is also supported.

The band-pass filter parameters are set from 0.01 to 0.08 Hz with a filter order of 2 by default, as used inPower et al. (2014). The filter is calculated using scipy’s (Virtanen et al., 2020) butter functionality to calculate filtering coefficients, and filtfilt to apply the filter to the data. The filter uses constant padding with the maximum allowed pad length of one less than the total number of volumes. Parameters can be modified in the command line, using the – –lower–bpf, – –upper–bpf, and – –bpf–order flags.

### 2.12 Confound regression

Confound regression is used to mitigate non-neural noise in fMRI scans, including but not limited to: motion-related artifacts, arterial carbon dioxide concentration, respiratory and cardiac cycles, and blood pressure (Caballero-Gaudes & Reynolds, 2017;Murphy et al., 2013;Pham et al., 2023). XCP-D implements denoising via linear least squares regression. Notably, regression is only performed on low-motion data; the linear model is fit to the low-motion volumes from both the BOLD data and the confounds, and the parameter estimates are retained. This procedure is used because the inclusion of high motion data that is removed via temporal censoring would reduce the effectiveness of confound regression (Power et al., 2014). The parameter estimates from the low-motion volumes are then used to denoise the interpolated data (Lemieux et al., 2007).

In some cases, the selected confounds may be correlated with signals of interest, as in AROMA, where ICA components are labeled as “noise” or “signal.” In these cases, including the “noise” regressors without modification can result in the removal of variance explained by “signal” regressors. To address this issue, XCP-D orthogonalizes all nuisance regressors with respect to any detected signal regressors. This is done automatically for nuisance regression strategies that include AROMA regressors. For custom confounds derived from spatial ICA components, such as multi-echo denoising with Tedana (DuPre et al., 2021;Kundu et al., 2011,^2013^), users may include “signal” components in their custom confounds file, named using the prefix “signal__.” When columns with this prefix are detected in the confounds file, XCP-D will automatically employ this orthogonalization procedure. When the confound regression step is performed, the modified nuisance regressors (i.e., without the signal regressors) will be mean-centered, censored to remove high-motion volumes, and finally regressed out of the fMRI data. For custom confounds, every column in the provided file that is not prefixed with “signal__” will be used in the regression.

The interpolated version of the post-processed data is written out, with “desc-interpolated” in the timeseries filename; this allows for backward compatibility with several existing tools in the community. These full-length timeseries can be leveraged for quality control to examine consistency in total run length across participants and to examine consistency of censored frames and artifacts in the Executive Summaries. They can also be leveraged to generate new correlation matrices that may adjust for differing censoring thresholds for a given use case without having to reprocess data through XCP-D (e.g., leveraginghttps://github.com/DCAN-Labs/biceps). The full-length regressed data are also fed into the temporal censoring workflow.

### 2.13 Temporal censoring

Temporal censoring (also known as motion scrubbing) is a process in which data points with excessive motion are removed from the fMRI timeseries (Power et al., 2012). It has been shown that temporal censoring can reduce spurious motion-related group differences to chance levels (Power et al., 2014). Additionally, regression-based denoising is less effective against transient/intense noise, referred to as “burst” noise. In such settings, the complementary approach of scrubbing can be beneficial (Pham et al., 2023). The FD threshold specified by the user (with a default value of 0.3) is used to identify volumes to be censored. Temporal censoring can be disabled by setting – –fd–thresh to 0.

Using the temporal mask generated earlier (see*Interpolation*), the interpolated data from both the BOLD data and the confounds are removed, producing a denoised timeseries with variable length timeseries outputs. Users who want to consider generating connectivity matrices across participants with consistent (i.e., same length) timeseries can use the – –exact–time flag in XCP-D (see*Parcellated timeseries extraction and calculation of connectivity matrices*).

For participants with high motion, it is possible that censoring results in a timeseries with few uncensored volumes. XCP-D allows the user to specify a minimum run duration (in seconds) of data that should remain after censoring. This minimum time, in seconds, can be specified by the user via – –min–time (with a default value of 240 seconds), which determines the minimum amount of time, in seconds, needed to process a given run once high-motion volumes are removed. These volumes may be non-contiguous. This feature can be disabled by providing a 0 or a negative value.

If censoring is applied, parcellated timeseries and connectivity matrices come from censored data.

### 2.14 Parcellated timeseries extraction and calculation of connectivity matrices

Functional connectivity matrices are a widely used approach to examine the coherence in activity between distant brain areas (Biswal et al., 1995;Hlinka et al., 2011). The generation of these matrices involves parcellating the brain into regions determined by atlases and then calculating correlations between regions.

XCP-D extracts voxel-wise timeseries from the censored, denoised BOLD timeseries and outputs parcellated timeseries and correlation matrices for a variety of atlases bundled in the software. The local mean timeseries within each brain atlas’s region of interest (ROI) is extracted via Nilearn’s (Abraham et al., 2014) NiftiLabelsMasker for NIfTIs, and ConnectomeWorkbench’s wb_command –cifti–parcellate function for CIFTIs. Functional connectivity matrices are estimated using the Pearson correlation between all parcels for a given atlas. Before functional connectivity is estimated, any timeseries of all zeros (indicating voxels/vertices in the atlas that are not covered in the BOLD data) are replaced with NaNs, so that the mean timeseries from each parcel reflects the average of only covered voxels/vertices. The coverage threshold (with a default value of 0.5 or 50% coverage) applies to the warped versions of the atlases—therefore, if the normalization step removes a parcel, that parcel will contain NaNs in the parcellated timeseries.

If the – –exact–time flag is used, this parameter will produce correlation matrices limited to each requested amount of time (specified in seconds). If there is more than the required amount of low-motion data, then volumes will be randomly selected to produce denoised outputs with the exact amounts of time requested. If there is less than the required amount of “good” data, then the corresponding exact-time correlation matrix will not be produced.

The atlases currently used in XCP-D can be separated into three groups: subcortical, cortical, and combined cortical/subcortical. The two subcortical atlases are the Tian atlas (Tian et al., 2020) and the CIFTI subcortical parcellation (Glasser et al., 2013). The cortical atlases are the Glasser (Glasser et al., 2016) and the Gordon (Gordon et al., 2016). The combined cortical/subcortical atlases include 10 different resolutions of the 4 seconds (Schaefer Supplemented with Subcortical Structures) atlas, and several thresholded probabilistic atlases from the MIDB Precision Brain Atlas (Hermosillo et al., 2024).

The 4 seconds atlas combines theSchaefer 2018cortical atlas (version v0143;Schaefer et al., 2018) at 10 different resolutions (100, 200, 300, 400, 500, 600, 700, 800, 900, and 1,000 parcels) with the CIT168 subcortical atlas (Pauli et al., 2018), the Diedrichson cerebellar atlas (King et al., 2019), the HCP thalamic atlas (Najdenovska et al., 2018), and the amygdala and hippocampus parcels from the HCP CIFTI subcortical parcellation (Glasser et al., 2013). Notably, all atlases have been harmonized with QSIPrep (Cieslak et al., 2021) and ASLPrep (Adebimpe et al., 2022) to facilitate multi-modal network analyses.

### 2.15 ReHo

Regional Homogeneity (ReHo) is a measure of local temporal uniformity in the BOLD signal computed at each voxel of the processed image. Greater ReHo values correspond to greater synchrony among BOLD activity patterns measured in a local neighborhood of voxels (Zang et al., 2004). ReHo is calculated as the coefficient of concordance among all voxels in a sphere centered on the target voxel (Zuo et al., 2013).

ReHo is performed on the denoised BOLD file, and the output is written out directly to the XCP-D derivatives folder. For NIfTIs, ReHo is always calculated via AFNI’s 3dReho with 27 voxels in each neighborhood, using Kendall’s coefficient of concordance (KCC) (Zhang et al., 2023). For CIFTIs, the left and right hemisphere are extracted into GIFTI format via Connectome Workbench’s CIFTISeparateMetric. Next, the mesh adjacency matrix is obtained, and KCC is calculated with each vertex having four neighbors. For subcortical voxels in the CIFTIs, 3dReho (Taylor & Saad, 2013) is used with the same parameters that are used for NIfTIs.

### 2.16 ALFF

The amplitude of low-frequency fluctuations (ALFF)—also called “fluctuation amplitude”—is a measure of regional intensity of BOLD signal fluctuation (Yu-Feng et al., 2007) calculated in each voxel of the processed image. Low-frequency fluctuations are of particular importance because functional connectivity is most typically computed based on synchronous, low-frequency fluctuations (Zou et al., 2008).

ALFF is calculated on the denoised BOLD file and its output can optionally be smoothed (see*Smoothing*). ALFF is only calculated if band-pass filtering is applied. ALFF is computed by transforming the processed BOLD timeseries to the frequency domain. When motion censoring is disabled, XCP-D uses scipy’s (Virtanen et al., 2020) periodogram function. When censoring is enabled, XCP-D uses scipy’s (Virtanen et al., 2020) LombScargle periodogram. The power spectrum is computed within the frequency band defined by the band-pass values used for temporal filtering, and the mean square root of the power spectrum is calculated at each voxel to yield voxel-wise ALFF measures.

### 2.17 Spatial smoothing

Noise in the BOLD signal—due to physiological signals or scanner noise—can introduce spurious artifacts in individual voxels (Mikl et al., 2008). The effects of noise-related artifacts can be mitigated by spatial smoothing of the data, which can dramatically increase the signal-to-noise ratio (Mikl et al., 2008). However, spatial smoothing is not without its costs: it effectively reduces volumetric resolution by blurring signals from adjacent voxels (Mikl et al., 2008).

Spatial smoothing optionally occurs after temporal filtering; smoothing is applied to the denoised BOLD file and ALFF outputs. Note that ReHo outputs are not smoothed. FWHM smoothing is implemented in XCP-D with a default value of 6.0 mm (2.55σ) in both volumes and surfaces. Additionally, ALFF maps are also smoothed if the – –smoothing flag is specified by the user. Smoothing for NIfTIs is performed via Nilearn’s (Abraham et al., 2014) smooth_img using a Gaussian filter. For CIFTIs, the specified FWHM kernel (specified in mm) is converted to sigma (standard deviation). Connectome Workbench’s (Marcus et al., 2011) wb_command –cifti–smoothing is used to smooth each hemisphere and the subcortical volumetric data.

### 2.18 Quality control

XCP-D calculates multiple quality control measures. These include estimates of fMRI data quality before and after regression, as well as indices of co-registration and normalization quality. Selected metrics include the following:

- Summary measures of realignment parameters: mean FD, mean and maximum root-mean-square displacement (RMS). FD and RMS measure relative contributions of angular rotation and uniformity of motion effects across the brain (Yan et al., 2013).
- DVARS: DVARS is a whole-brain measure of the temporal derivative (D) of image intensity computed by obtaining the root mean square variance across voxels (VARS; Goto et al., 2016.) As such, it reflects time-varying signals and large values are often attributable to artifacts such as in-scanner motion.
- fMRI-T1/T2 co-registration quality: Because of the limited spatial resolution and reduced anatomic contrast of fMRI images compared to structural images, fMRI images are co-registered to the structural image prior to normalization to template space. Poor co-registration can thus impact normalization. XCP-D calculates the Dice similarity index (Dice, 1945), overlap coefficient (Vijaymeena & Kavitha, 2016), and Pearson correlation between the fMRI image and the T1 image (or T2 image) to determine the quality of the registration. The Dice index equals twice the number of voxels common to both images divided by the sum of the number of voxels in each image. The overlap coefficient (Vijaymeena & Kavitha, 2016) calculates the relative number of non-zero voxels in both images. The Pearson’s correlation measures the correlations between the voxels in both images.
- fMRI-Template normalization quality: Following co-registration, the fMRI image is normalized to template space by applying the warp calculated in registration of the structural image to the template (Jahn et al., 2022). XCP-D calculates the Dice similarity index (Dice, 1945), overlap coefficient, and Pearson correlation coefficient to quantify the alignment of the fMRI image to the template.

### 2.19 Visual reports

**Fig. 2.**
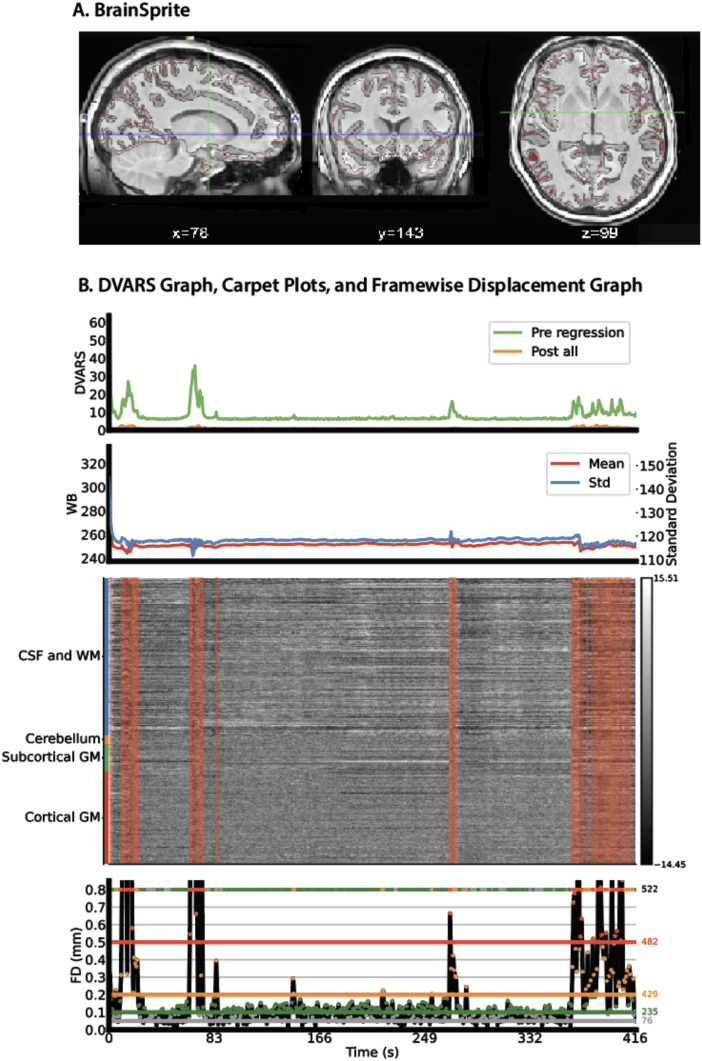
Selected elements of the XCP-D Executive Summary. Panel A depicts the BrainSprite viewer that overlays white and pial matter on the template, followed by (Panel B) a carpet plot and graphs depicting FD and DVARS. FD: Framewise displacement; DVARS: temporal derivative (D) of image intensity computed by obtaining the root mean square variance across voxels (VARS).

XCP-D produces two different user-friendly, interactive HTML reports. The first output is called the “Executive Summary.” The Executive Summary is an interactive web page for quick visual inspection of structural and functional registration, surface quality, physiological and non-physiological artifacts, and post-processing success (see select elements inFig. 2; a full example of a subject from OpenNeuro is provided inSupplemental Fig. 1; subject downloaded fromhttps://github.com/OpenNeuroDatasets/ds004078). It is particularly useful in surface-based post-processing for assessing co-registration, normalization, and surface alignment and quality. For example, it includes an interactive BrainSprite (https://GitHub.com/brainsprite/brainsprite) viewer that overlays pial and white matter surfaces on the template image. This allows users to quickly assess the quality of the surface registration. Further information regarding co-registration and normalization quality is depicted in contour plots. The Executive Summary also includes a carpet plot for all runs, which depicts the fMRI timeseries before and after confound regression. These carpet plots are displayed alongside the FD plots and DVARS timeseries to allow users to rapidly assess denoising success. Additionally, XCP-D also provides a NiPreps style report that depicts similar information in a different layout (seeSupplemental Fig. 2for an example; subject downloaded fromhttps://github.com/OpenNeuroDatasets/ds004078). In both reports, XCP-D also produces a “methods boilerplate” that details the methods applied along with citations as relevant for users. This automatically generated description of the methods ensures fidelity of reporting and can be directly copied into publications’ methods sections.

### 2.20 Anatomical workflow

The workflow can be enabled via the – –warp–surfaces–native2std flag. It serves two main purposes. First, it is used to warp several surfaces derived from the structural images from fsnative to fsLR (Freesurfer templates;Van Essen et al., 2012) space, which is useful as part of the visual reports for assessing normalization to the fsLR template. To this end, the workflow generates surf.gii files in fsLR space for the gray matter/pial matter border and the white matter/gray matter border. It also generates HCP-style inflated surfaces for visualization purposes.

Second, XCP-D will parcellate morphometric surface files—including cortical thickness, depth, and curvature—generated in pre-processing by Freesurfer (Fischl, 2012), sMRIPrep (Esteban et al., 2020), and/or HCP (Glasser et al., 2013) pipelines. XCP-D parcellates these morphometric files using the same atlases that are used for creating functional connectivity matrices as well as other surface features like ALFF and ReHo. This functionality facilitates analyses of both fMRI and structural imaging features when data are processed using XCP-D.

### 2.21 Concatenation

XCP-D also offers users the option of concatenating fully denoised timeseries across fMRI runs based on the run and dir entities (notably, different tasks are not concatenated); this also yields QC metrics that are concatenated. Notably, this option should be used with some caution as it will double the size of output data in the derivatives folder. Users can concatenate runs by specifying the – –combineruns flag.

## 3 Results

Below, we demonstrate the utility of XCP-D in two ways. First, we provide a detailed walkthrough with bundled example data. Second, we apply it to data from three large-scale datasets.

### 3.1 Walkthrough: The XCP-D workflow for processing an fMRIPrep dataset

The following walkthrough details the workflow for post-processing a dataset using XCP-D on an HPC—specifically, a RedHat Enterprise Linux-based system, using Apptainer (Kurtzer et al., 2017). To do so, we use an example dataset that is bundled with the software within the container. This container contains three example subjects from a study on executive function, which is available on OpenNeuro athttps://openneuro.org/datasets/ds004450. These subjects are organized in a BIDS-compatible manner (K. J. Gorgolewski et al., 2016) with T1s, two resting-state runs, and corresponding field maps for the three subjects. Both .nii.gz and .json files are available for each of these scans, along with a dataset_description.json, and fMRIPrep (Esteban et al., 2019) derivatives. For the purposes of this walkthrough, commands for a minimal XCP-D run will be demonstrated.

All commands are run in a directory named XCPD_test. The XCP-D walkthrough container with the bundled subjects can be downloaded via Apptainer (Kurtzer et al., 2017), by running the following bash script:

singularity build xcp_walkthrough.sif docker://pennlinc/xcp_walkthrough:main

XCP-D can then be run on example subjects via Apptainer (Kurtzer et al., 2017), by running the following bash script:

#$ –l h_vmem = 40G

singularity run –cleanenv –B ~/XCPD_test ~/XCPD_test/xcp_walkthrough.sif /data/EF/derivatives/fmriprep ~/XCPD_test/output –fs–license–file <path_to_Freesurfer_license_file> participant –vv

This script runs XCP-D using all the default options. The – –cleanenv flag ensures that environment variables from local machines are ignored so that appropriate packages from within the container are used, and –B mounts the input files on local devices to the image. The three arguments here correspond to the mandatory arguments for the pre-processing derivatives directory (/data/EF/derivatives/fmriprep), the output directory (~/XCPD_test/output), and the analysis level (participant).

**Fig. 3.**
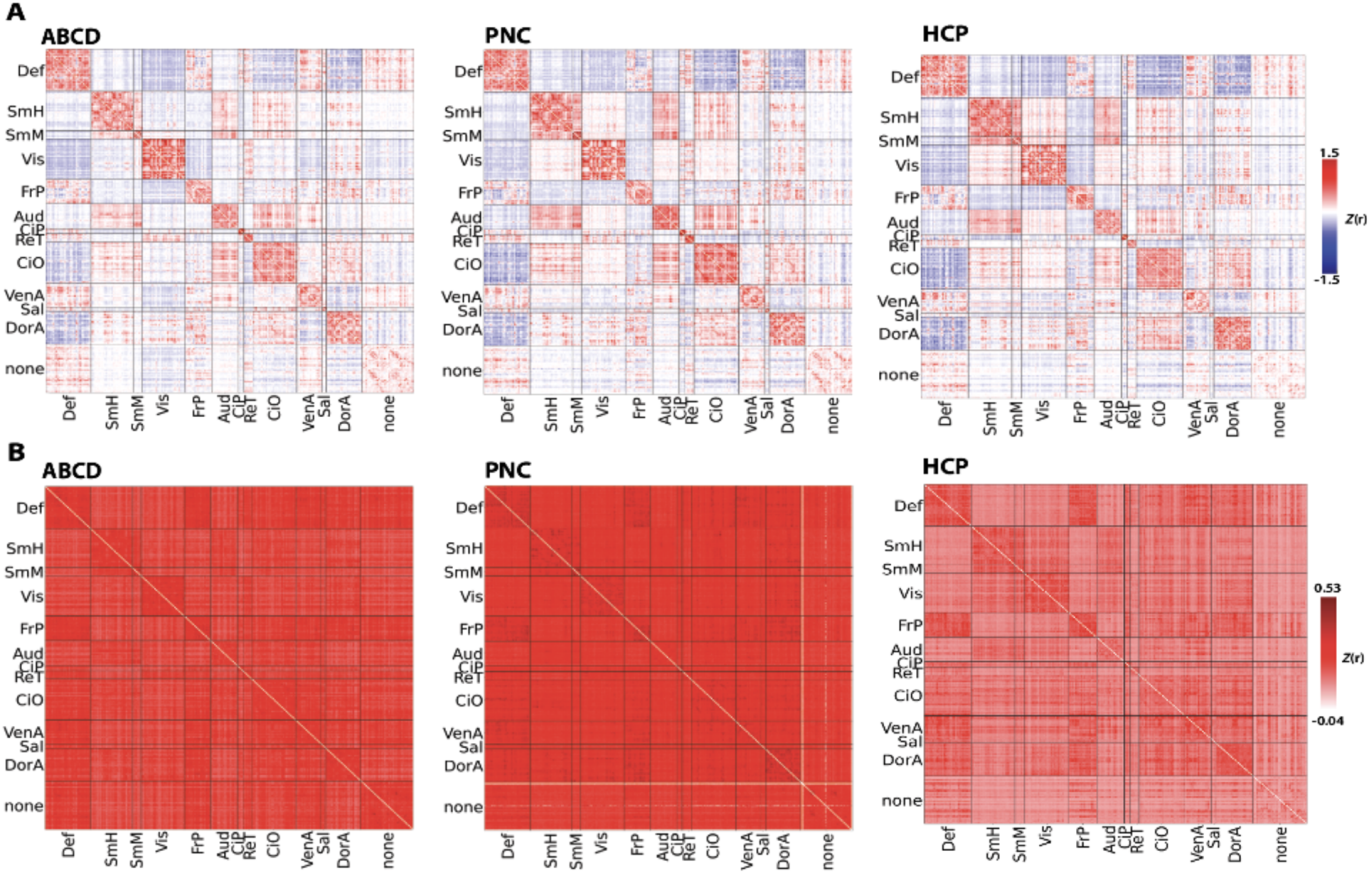
Mean (Panel A) and standard deviation (Panel B) of functional connectivity generated by XCP-D for each dataset in our large-scale application, displayed after Fisher’s*Z*transformation. Data are displayed using the Gordon atlas (Gordon et al., 2016). Def: default mode network; SmH: somatomotor hands network; SmM: somatomotor mouth network; Vis: visual network; FrP: Frontoparietal network; Aud: auditory network; CiP: cinguloparietal network; CiO: cingulo-opercular network; VenA: ventral attention network; Sal: salience network; DorA: dorsal attention network.

This will produce XCP-D derivatives under the folder XCPD_test/output. The outputs will include a dataset description, logs, citation information, processed anatomical and functional derivatives, as well as .svg figures. SeeSupplemental Figure 3for the expected directory structure of output from one example run.

### 3.2 Application of XCP-D to three example datasets

To illustrate the utility of XCP-D to diverse data, we processed a total of 600 subjects from three datasets. Specifically, we processed n = 200 participants each from the Philadelphia Neurodevelopmental Cohort (PNC;Satterthwaite et al., 2014,^2016^; mean age = 16.477; standard deviation of age = 3.029; 56% female), the Human Connectome Project - Young Adults (HCP-YA;Glasser et al., 2013; mean age = 28.595; standard deviation of age = 3.732; 54.5% female) sample, and the Adolescent Brain Cognitive Development study^®^(ABCD;Volkow et al., 2018; mean age = 12.011; standard deviation of age = 0.611; 55.3% female). Note that the ABCD data repository grows and changes over time. The ABCD data used in this report came fromhttps://doi.org/10.17605/OSF.IO/PSV5M. All component studies were approved by local institutional review boards.

Critically, prior to post-processing with XCP-D, each of these datasets were pre-processed using different tools. The PNC sample was pre-processed using fMRIPrep (Esteban et al., 2019), the ABCD sample was pre-processed using ABCD-BIDS (Feczko et al., 2021), and the HCP-YA sample was pre-processed via the HCP minimal processing pipeline (Glasser et al., 2013). All testing data had high-quality structural images (Rosen et al., 2018) and greater than 5 minutes of high-quality resting-state fMRI data.

Note that the ABCD-BIDS (Feczko et al., 2021) and PNC (Satterthwaite et al., 2014,^2016^) datasets were processed with version 0.5.1 of XCP-D, while HCP-YA (Glasser et al., 2013) was processed with version 0.6.0rc4. The following command was used to process the data (via the CIFTI surface-based workflow, with the anatomical workflow enabled):

PNC:

singularity run –cleanenv –B ${PWD} –env FS_LICENSE=${PWD}/code/license.txt pennlinc–containers/.datalad/environments/xcp/image ${PWD}/inputs/data/fmriprep xcp participant– combineruns –nthreads 1 –omp–nthreads 1 –mem_gb 10 –smoothing 2 –min_coverage 0.5 –min_time 100 –dummy–scans auto–random –seed 0 –bpf–order 2 –lower–bpf 0.01 –upper–bpf 0.08 –motion–filter–type lp –band–stop–min 6 –motion–filter–order 4 –head–radius auto –exact–time 300 480 600 –despike–participant_label $subid –p 36P –f 0.3 –cifti –warp–surfaces–native2std –dcan–qc –w ${PWD}/.git/tmp/wkdir –vvv –input–type fmriprep

ABCD:

singularity run –cleanenv –B ${PWD} pennlinc–containers/.datalad/environments/xcp/image inputs/data xcp participant –combineruns –nthreads 1 –omp–nthreads 1 –mem_gb 10 –smoothing 2 –min_coverage 0.5 –min_time 100 –dummy–scans 6 –random–seed 0 –bpf–order 2 –lower–bpf 0.01 –upper–bpf 0.08 –motion–filter–type notch –band–stop–min 15 –band–stop–max 25 –motion–filter–order 4 –head–radius auto —exact–time 300 480 600 –despike –participant_label $subid –p 36P –f 0.3 –cifti –warp–surfaces–native2std –dcan–qc –w ${PWD}/.git/tmp/wkdir –v –input–type dcan

HCP-YA:

singularity run –cleanenv –B ${PWD} pennlinc–containers/.datalad/environments/xcp/image inputs/data xcp participant –combineruns –nthreads 1 –omp–nthreads 1 –mem_gb 10 –smoothing 2 –min_coverage 0.5 –min_time 100 –dummy–scans 7 –random–seed 0 –bpf–order 2 –lower–bpf 0.01 –upper–bpf 0.08 –motion–filter–type notch –band–stop–min 12 –band–stop–max 18 –motion–filter–order 4 –head–radius auto –exact–time 300 480 600 –despike –participant_label $subid –p 36P –f 0.3 –cifti –warp–surfaces–native2std –dcan–qc –w ${PWD}/.git/tmp/wkdir –v –input–type hcp

**Fig. 4.**
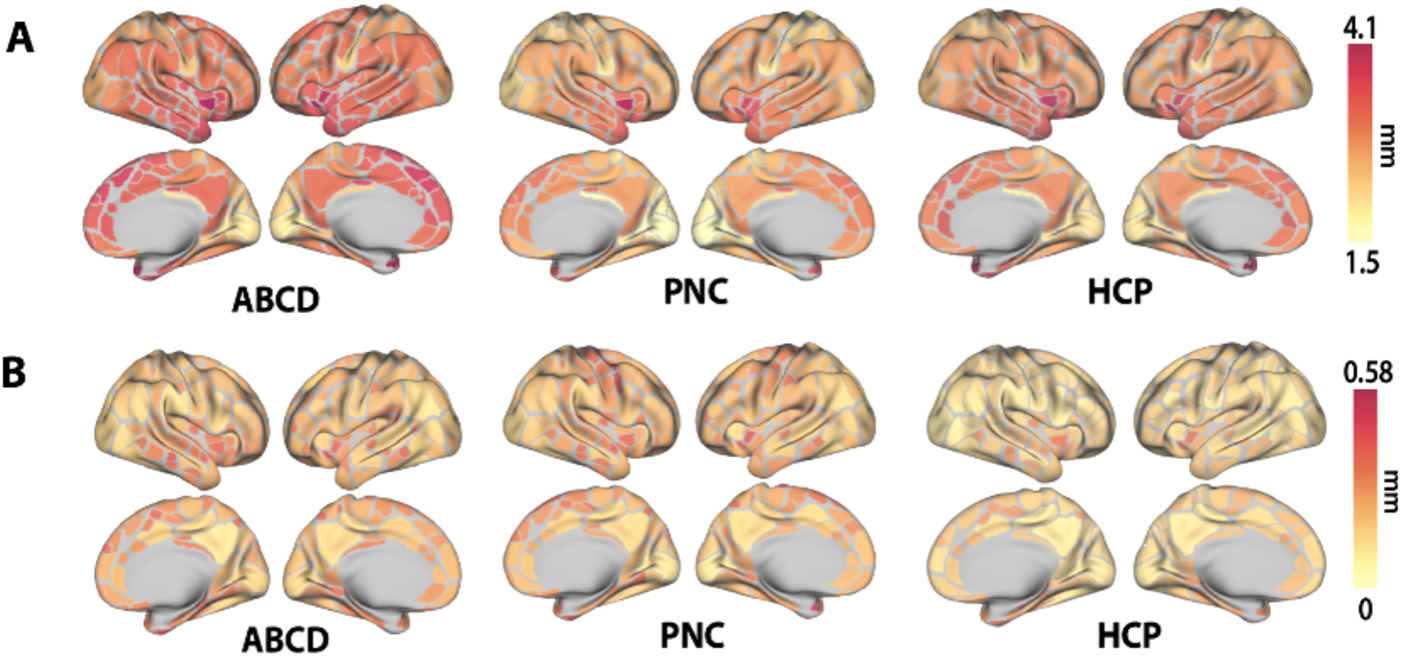
The mean (Panel A) and standard deviation (Panel B) of cortical thickness calculated by XCP-D for each dataset in our large-scale application. Data are displayed using the Gordon atlas (Gordon et al., 2016).

XCP-D completed successfully for all participants in all datasets. Among other outputs, XCP-D generated functional connectivity matrices (Fig. 3) and parcellated cortical thickness information for each participant (Fig. 4). Two small parcels in the medial temporal lobe cortex lacked coverage in the PNC. Notably, the correlation between the mean connectivity matrices was 0.93 for ABCD and PNC, 0.90 for ABCD and HCP-YA, and 0.92 for PNC and HCP-YA. The correlation between cortical thickness measures was 0.90 for ABCD and PNC, 0.95 for ABCD and HCP-YA, and 0.85 for PNC and HCP-YA.

## 4 Discussion

Functional imaging is an essential tool for human neuroscience research. In contrast to pre-processing, where the field has gravitated towards use of standardized pipelines such as fMRIPrep (Esteban et al., 2019), there has been a relative lack of standardization in fMRI post-processing (Botvinik-Nezer et al., 2020). While several options for post-processing exist, they are often incompatible with common pre-processing methods, lack standardized output, and may not include software engineering best practices such as CI testing. While the steps used to generate the minimally pre-processed data are often quite similar, post-processing strategies used and derived measures often diverge substantially across data resources (Botvinik-Nezer et al., 2020). XCP-D seeks to fill this gap and provide a post-processing workflow that is compatible with data pre-processed with several widely used strategies. XCP-D’s open and modular codebase in Nipype (K. Gorgolewski et al., 2011) includes extensive CI testing, produces many measures of quality control, and yields analysis-ready derived measures that are named according to the BIDS standard. Together, XCP-D provides rigorous, accessible, and generalizable fMRI post-processing.

The derived measures generated by XCP-D include many of the most broadly used features of brain function and structure. Functional measures include connectivity matrices from multiple atlases as well as voxel- and vertex-wise maps of fluctuation amplitude (ALFF) and ReHo. While XCP-D does not include extensive structural image processing or image registration, it does consume the structural features generated by pre-processing pipelines, rename them according to current BIDS standards (K. J. Gorgolewski et al., 2016), and apply the same parcellations used for the functional images. Summarizing functional and structural features in the many contemporary atlases included in XCP—including multi-scale atlases like the Schaefer parcellation (Schaefer et al., 2018)—facilitates multi-modal data integration and analysis. Multi-modal analyses are further accelerated by the recent integration of this same atlas bundle into our existing pipelines for diffusion MRI processing (QSIPrep;Cieslak et al., 2021) and processing arterial spin-labeled MRI (ASLPrep;Adebimpe et al., 2022) for calculation of cerebral blood flow.

Beyond such analysis-ready derived features, XCP-D produces an extensive set of quality control measures. These measures include indices of both image registration (e.g., Dice coefficient;Dice, 1945) and denoising performance (e.g., the correlation of DVARS and motion before and after denoising). Together, such measures facilitate scalable quality assurance for large datasets and allow users to identify problematic datasets that can be further evaluated using the detailed reports generated for each participant. As part of our “glass-box” design philosophy, these single-participant reports allow users to examine key intermediate steps in the processing workflow. One particularly useful feature is the interactive BrainSprite (https://github.com/brainsprite/brainsprite) that depicts the fully processed structural images along with overlays of the functional images. This visualization allows users to rapidly assess the success of image co-registration and atlas normalization. Additionally, the report includes tailored carpet plots that display the functional timeseries before and after post-processing, facilitating rapid visualization of artifacts related to in-scanner motion. Each participant’s report closes with an automatically generated boilerplate summary of the methods used by XCP-D for the configuration specified, along with relevant citations and references. This text enables users to determine if the desired processing occurred as expected and ensures accurate methods reporting.

In the design of XCP-D, we integrated multiple software engineering features to ensure stability and rigor. First, all XCP-D development is open, version-controlled, and clearly documented via detailed pull requests on GitHub. XCP-D implements branch protection rules that require reviews from at least one XCP-D developer before pull requests can be merged or changes can be released. We have benefited from substantial community input and strive to quickly respond to bug reports from users. Second, XCP-D has a highly modular design in Nipype (K. Gorgolewski et al., 2011) to reduce code duplication, enforce standardized workflows, facilitate integration testing, and allow for extensibility over time. Third, XCP-D is a BIDS-App, and we have made every effort to adhere to the standards described by BIDS (K. J. Gorgolewski et al., 2016), including the BIDS extension proposals (BEPs) related to derived data and functional networks. Fourth, XCP-D modules are subjected to extensive CI testing using CircleCI. These tests do not simply check that a file was produced but draw upon diverse example data and knowledge of each module’s operation to ensure that processing was executed correctly (e.g., checking that a spike in the data is no longer present after despiking). These tests make the software more sustainable over time and mitigate risk of updates introducing occult errors. We track CI coverage using CodeCov; at present, 83% of the XCP-D codebase is covered by CI tests. Fifth and finally, XCP-D is containerized and distributed via Docker (Rad et al., 2017) and Apptainer (Kurtzer et al., 2017), which wrap all dependencies to allow the software to be easily deployed in most computing environments. Notably, XCP-D has achieved Gold tier for documentation, Silver tier for infrastructure, and Silver tier for testing according to NMIND standards (Kiar et al., 2023; seeSupplemental Fig. 4).

There are many tools that denoise fMRI data, produce resting-state derivatives, and/or produce structural derivatives, including C-PAC (Configurable Pipeline for the Analysis of Connectomes;Craddock et al., 2013), Conn (Whitfield-Gabrieli & Nieto-Castanon, 2012), connectomemapper3 (Tourbier et al., 2022), CCS (Connectome Computation System;Xu et al., 2015), and DPARSF (Data Processing Assistant for Resting-State fMRI;Chao-Gan & Yu-Feng, 2010). One major difference between these tools and XCP-D is our dedicated focus on consuming data pre-processed by other widely used tools such as fMRIPrep. As such, XCP-D fills an important niche in the neuroimaging software ecosystem. Much of the post-processing that XCP-D provides can be performed using tools included in Nilearn (Abraham et al., 2014), FSL (Woolrich et al., 2009), AFNI (Cox, 1996;Cox & Hyde, 1997), and other software libraries. However, this would require users to assemble a pipeline themselves from component tools, and as such necessitate a higher degree of methodological proficiency. Furthermore, such user-assembled custom pipelines inevitably result in greater heterogeneity of methods used and usually reduce generalizability across efforts.

XCP-D has several limitations. Although XCP-D currently offers multiple denoising options, the range of denoising options described in the literature is vast and many are not currently supported. For example, XCP-D does not provide dedicated support for physiological confounds such as respiration or heart rate measures (Frederick et al., 2012), although these signals can be modeled as a “custom confound” supplied by the user. Similarly, we do not currently support denoising methods such as phase regression, which suppresses signal from large veins by removing the linear fit between magnitude and phase timeseries from the magnitude timeseries (Knudsen et al., 2023). Also, XCP-D cannot be used to analyze task data. Such functionality is provided by FitLins (Markiewicz et al., 2022), NiBetaSeries (Kent & Herholz, 2019), and other packages. XCP-D also does not support group-level analyses. Furthermore, variance may also spread from interpolated to non-interpolated volumes. We are not aware of any methods that fully account for this. At the moment, XCP-D also does not support some pre-processing pipelines such as AFNI’s afni_proc.py (Taylor et al., 2018) and FSL (Woolrich et al., 2009), but these could potentially be added in the future. We also do not support a custom order of denoising operations. Finally, XCP-D does not provide mappings for users that utilize different pre-processing pipelines.

These limitations notwithstanding, XCP-D provides generalizable, accessible, and robust post-processing for fMRI data. XCP-D’s ability to post-process data from ABCD-BIDS (Feczko et al., 2021), HCP (Glasser et al., 2013), and fMRIPrep (Esteban et al., 2019) allows the same denoising, confound regression, and generation of derivatives for large-scale data resources that provide minimally pre-processed data; this could be invaluable for combining data across lifespan data resources. Moving forward, we plan to integrate additional advanced denoising methods, and provide dedicated methods for handling physiological data. In the future, we may also allow users to select derived output on an à la carte basis. As an open-source, collaborative software package, we welcome bug reports, feature suggestions, pull requests, and contributions from the community.

## Data and Code Availability

The Philadelphia Neurodevelopmental Cohort is a publicly available dataset athttps://www.ncbi.nlm.nih.gov/projects/gap/cgi-bin/study.cgi?study_id=phs000607.v3.p2. The Human Connectome Project dataset is also available athttps://www.humanconnectome.org/study/hcp-young-adult/data-releases, and the Adolescent Brain Cognitive Development Study can be obtained viahttps://nda.nih.gov/.

The codebase for XCP-D is on GitHub athttps://github.com/PennLINC/xcp_d.

## Author Contributions

K.M.—Conceptualization, Methodology, Software, Validation, Formal analysis, Investigation, Writing—original draft, Writing—review & editing, and Visualization. T.S.—Conceptualization, Methodology, Software, Validation, Supervision, Formal analysis, Investigation, Writing—original draft, Writing—review & editing, and Visualization. T.J.M.—Conceptualization, Software, and Writing—review & editing. A.A.—Conceptualization, Methodology, Software, and Writing—review & editing. D.S.B.—Methodology, Writing—review & editing, and Funding acquisition. M.B.—Conceptualization, Methodology, Software, Writing—original draft, and Writing—review & editing. M.C.—Conceptualization, Methodology, Software, Supervision, and Writing—review & editing. S.C.—Conceptualization, Methodology, Software, and Writing—review & editing. A.H.—Conceptualization, Writing—review & editing. A.S.K.—Writing—review & editing, Funding acquisition. J.T.L.—Conceptualization, Software, and Writing—review & editing. A.L.—Writing—review & editing. O.M.-D.—Conceptualization, Software, Methodology, and Writing—review & editing. S.M.N.—Conceptualization, Software, Methodology, and Writing—review & editing. G.S.—Writing—original draft, Writing—review & editing. S.S.—Writing—review & editing & funding. R.T.S.—Writing—review & editing, Funding acquisition. C.D.S.—Writing—review & editing. V.J.S.—Writing—review & editing. K.B.W.—Conceptualization, Writing—review & editing. E.F.—Conceptualization, Methodology, Software, and Writing—review & editing. D.A.F.—Conceptualization, Methodology, Formal analysis, Investigation, Writing—original draft, Writing—review & editing, Visualization, Supervision, and Funding acquisition. T.D.S.—Conceptualization, Methodology, Formal analysis, Investigation, Writing—original draft, Writing—review & editing, Visualization, Supervision, and Funding acquisition.

## Funding

This study was supported by grants from the National Institutes of Health: R37MH125829 (T.D.S. & D.A.F.), R01MH113550 (T.D.S. & D.S.B.), R01MH120482 (T.D.S.), R01MH112847 (T.D.S. & R.T.S.), R01EB022573 (T.D.S.), DA041148 (D.A.F.), DA04112 (D.A.F.), MH115357 (D.A.F.), MH096773 (D.A.F.), MH122066 (D.A.F.), MH121276 (D.A.F.), MH124567 (D.A.F.), NS129521 (D.A.F.), DP5OD036142 (S.S), T32NS091008 (S.S), the Brain & Behavior Research Foundation: 2022 Young Investigator Grant (S.S), the Burroughs Wellcome Fund: 2023 Career Award for Medical Scientists (S.S.), and the National Institute of Mental Health: 5T32MH019112-32 (A.S.K.). Additional support was provided by the Lynne and Andrew Redleaf Foundation, AE Foundation and the Penn/CHOP Lifespan Brain Institute. Data used in the preparation of this article were obtained from the Adolescent Brain Cognitive Development (ABCD) Study (https://abcdstudy.org), held in the NIMH Data Archive (NDA). This is a multisite, longitudinal study designed to recruit more than 10,000 children aged 9–10 and followed them over 10 years into early adulthood. The ABCD Study is supported by the National Institutes of Health and additional federal partners under award numbers U01DA041048, U01DA050989, U01DA051016, U01DA041022, U01DA051018, U01DA051037, U01DA050987, U01DA041174, U01DA041106, U01DA041117, U01DA041028, U01DA041134, U01DA050988, U01DA051039, U01DA041156, U01DA041025, U01DA041120, U01DA051038, U01DA041148, U01DA041093, U01DA041089, U24DA041123, and U24DA041147. A full list of supporters is available athttps://abcdstudy.org/federal-partners.html. A listing of participating sites and a complete listing of the study investigators can be found athttps://abcdstudy.org/consortium_members/. ABCD consortium investigators designed and implemented the study and/or provided data but did not necessarily participate in the analysis or writing of this report. This manuscript reflects the views of the authors and may not reflect the opinions or views of the NIH or ABCD consortium investigators.

## Declaration of Competing Interest

D.A.F. is a co-founder, director, and equity holder of Turing Medical, a medical imaging software company. M.B. is an employee of Turing Medical.

## Supplementary Materials

Supplementary material for this article is available with the online version here:https://doi.org/10.1162/imag_a_00257

## Supplementary Material

Supplementary Material

Supplemental_Table_1
