# Supplementary material for: XCP-D: A robust pipeline for the post-processing of fMRI data
Source: Imaging Neurosci (Camb). 2024 Aug 13;2:imag-2-00257. doi: 10.1162/imag_a_00257 (PMC12288603; doi:10.1162/imag_a_00257)
Supplement: Supplementary Material [file imag_a_00257-supp.pdf]

sub-01/None

BrainSprite Viewer: T1w

View T1w pngs

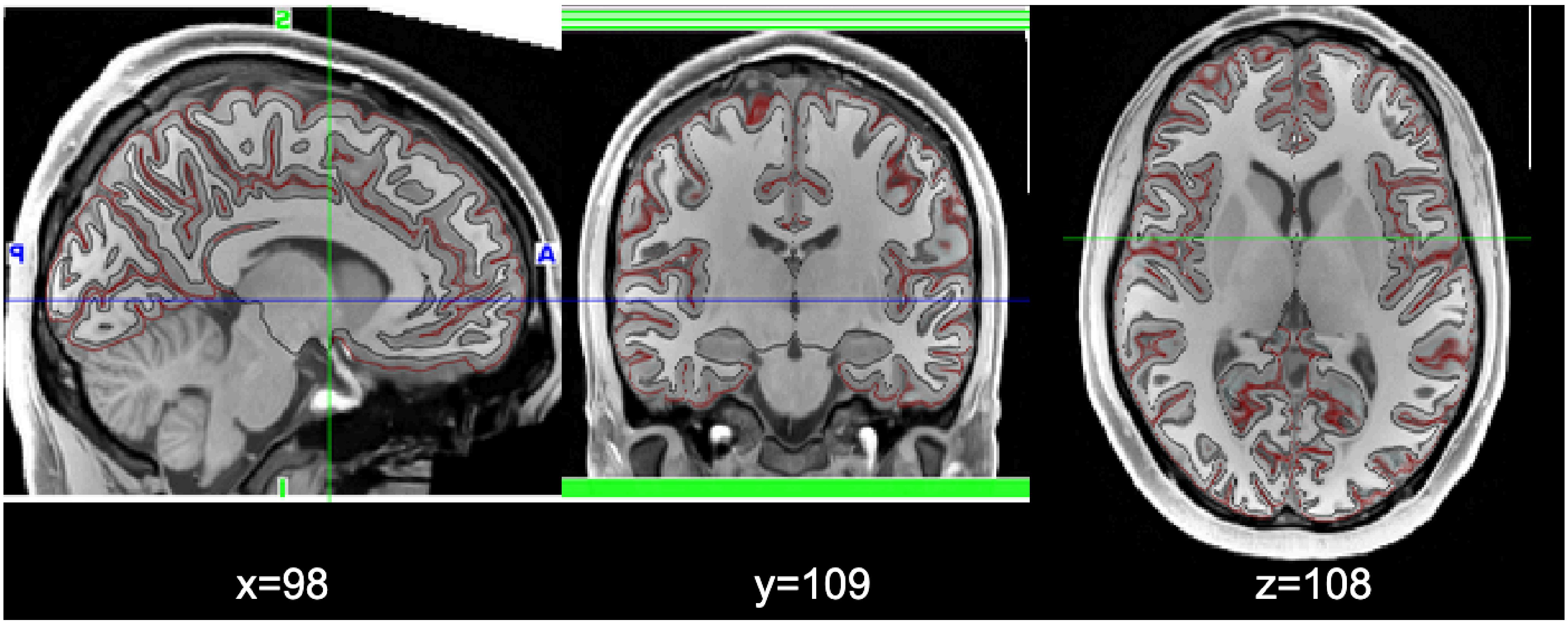

BrainSprite Viewer: T2w

View T2w pngs

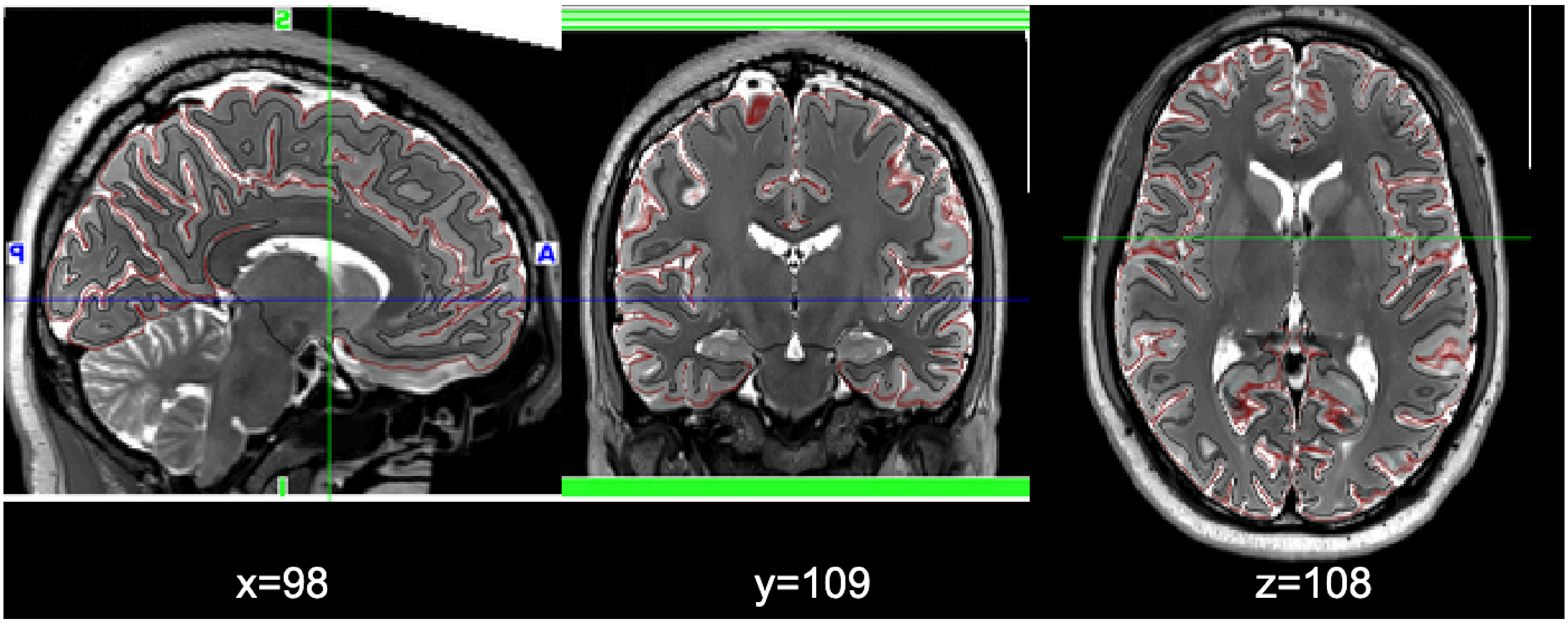

Anatomical Data

Atlas On T1w

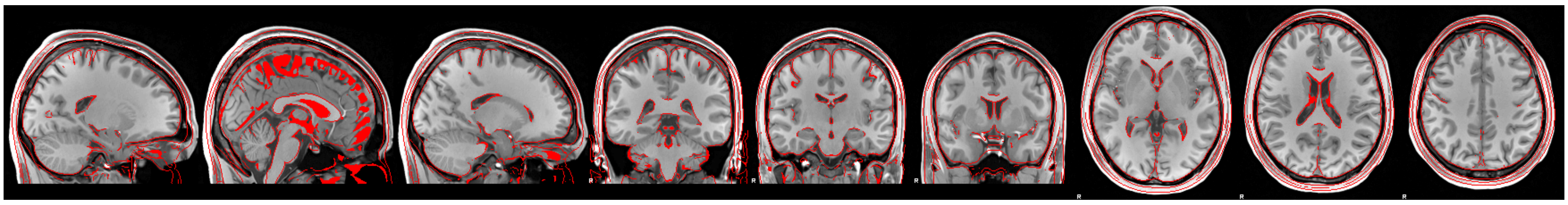

T1w On Atlas

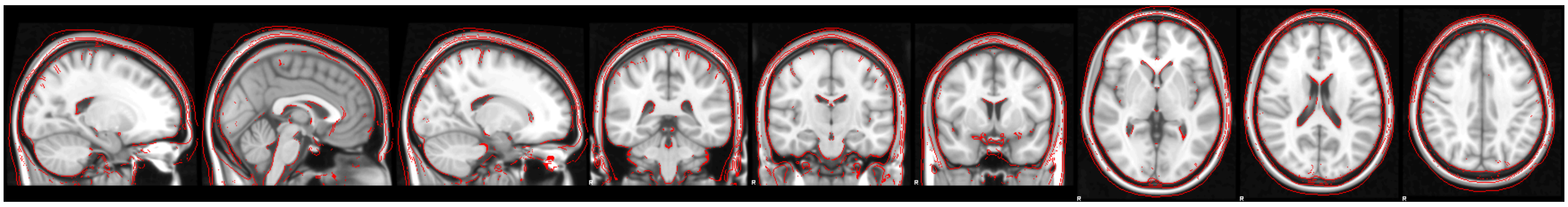

Atlas On T2w

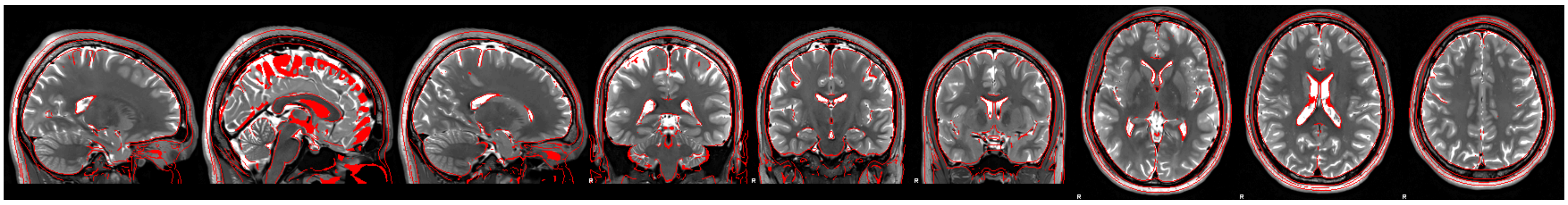

T2w On Atlas

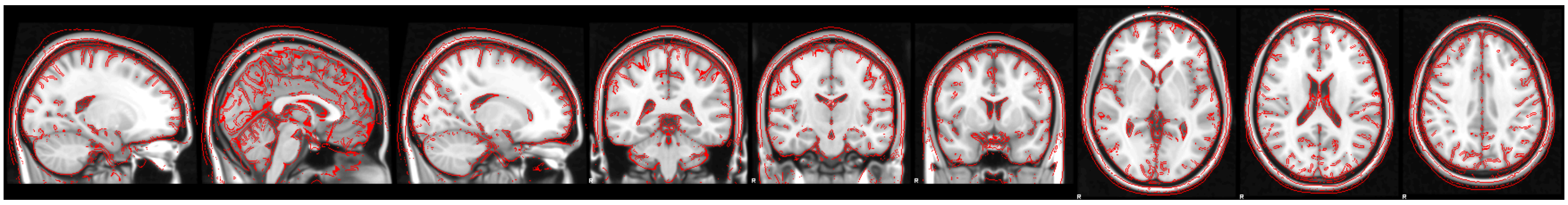

Functional Data

Combined Resting State Data

Pre-Regression

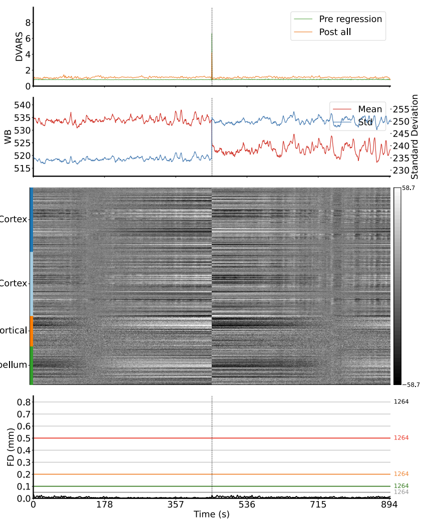

Post-Regression

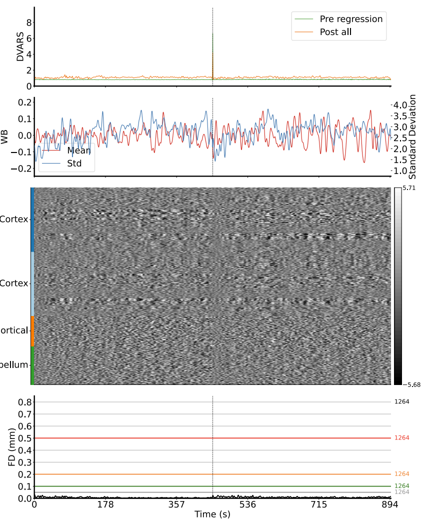

task-rest run-1:

Task On T1w

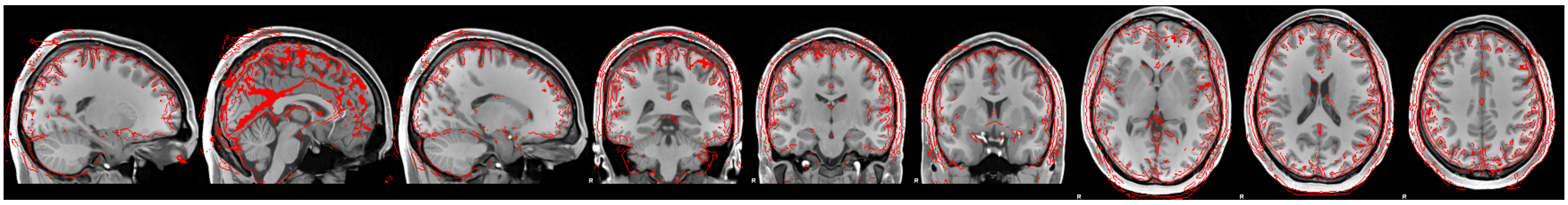

T1w On Task

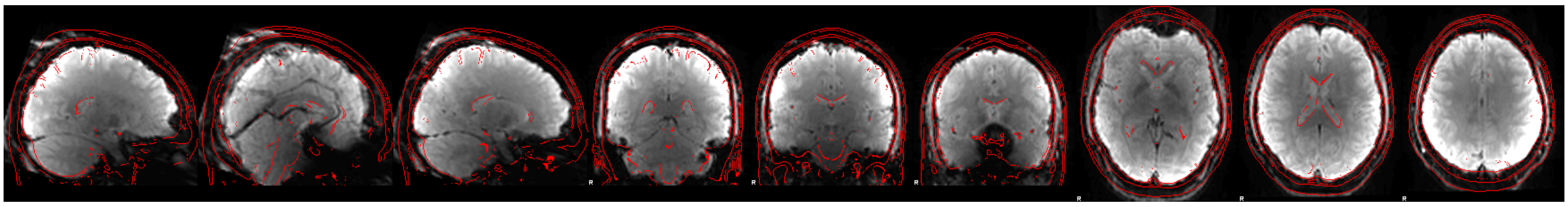

Task On T2w

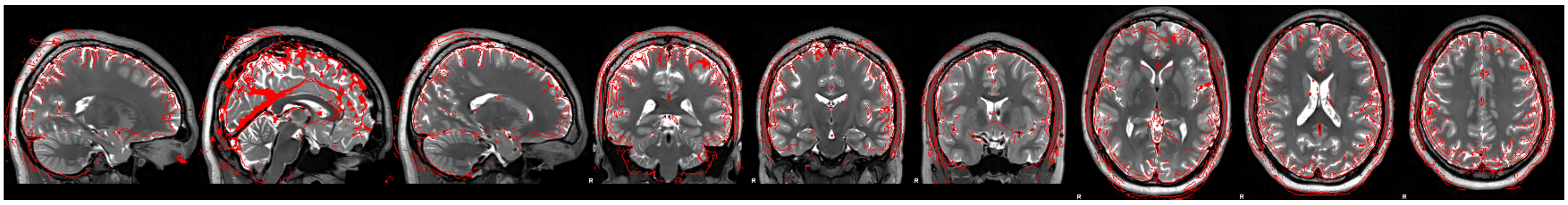

T2w On Task

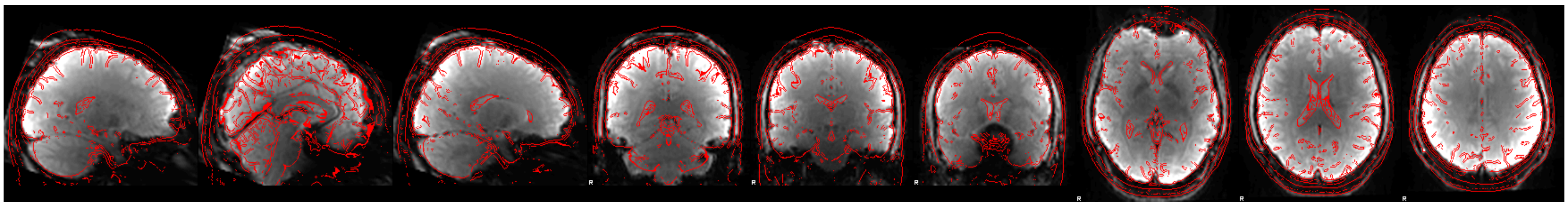

BOLD

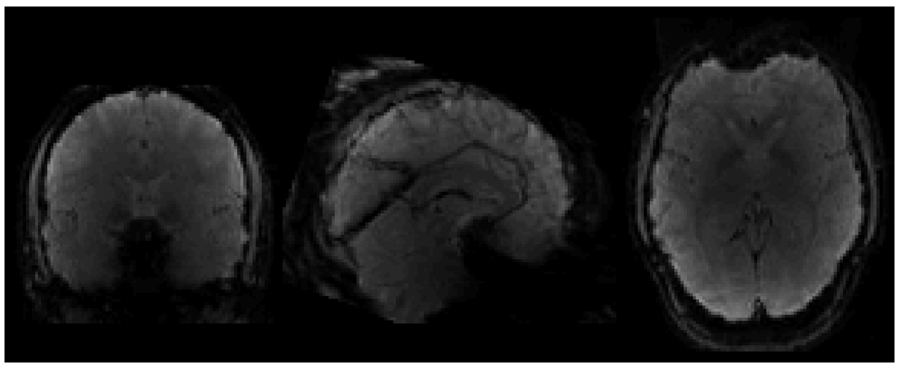

Reference

Pre-Regression

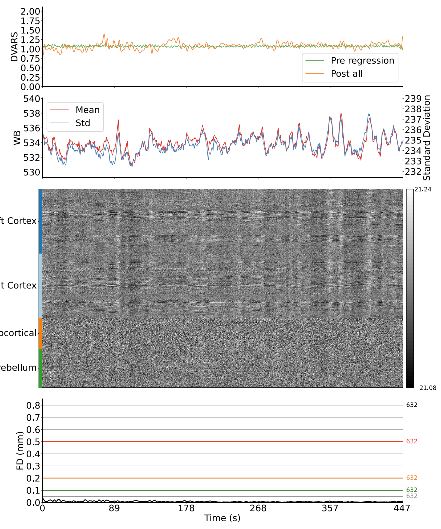

Post-Regression

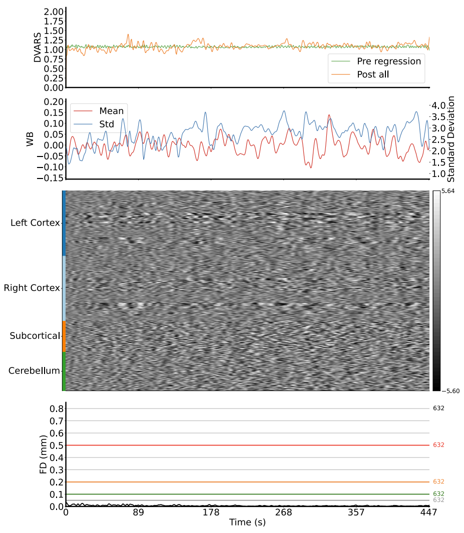

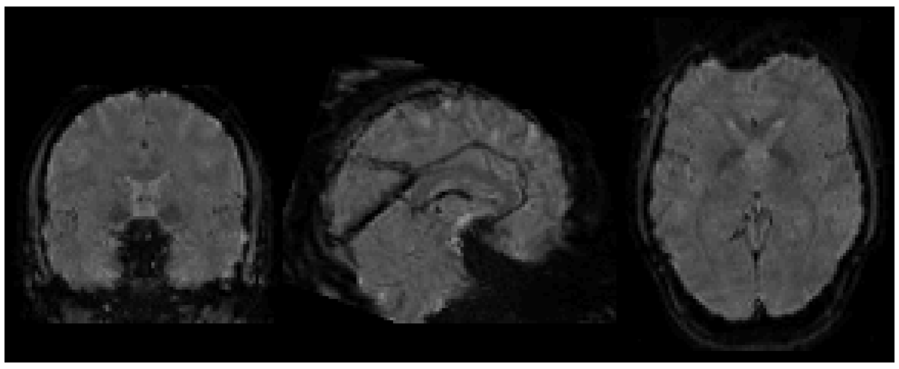

task-rest run-2:

Task On T1w

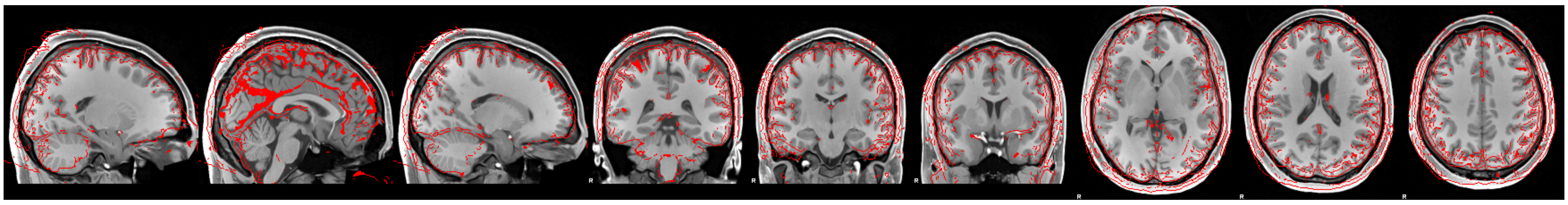

T1w On Task

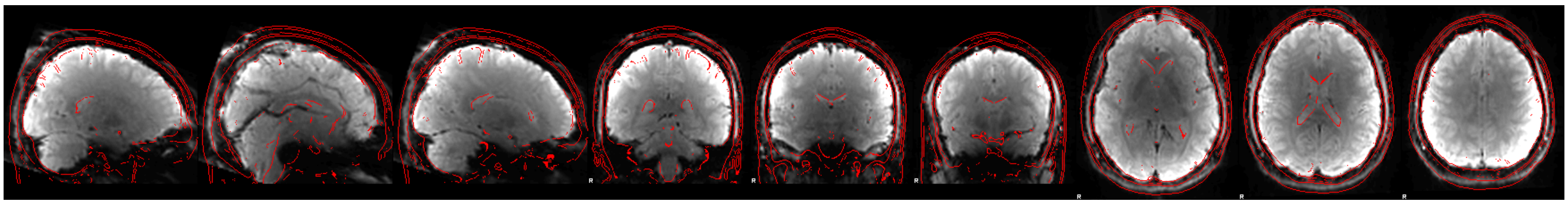

Task On T2w

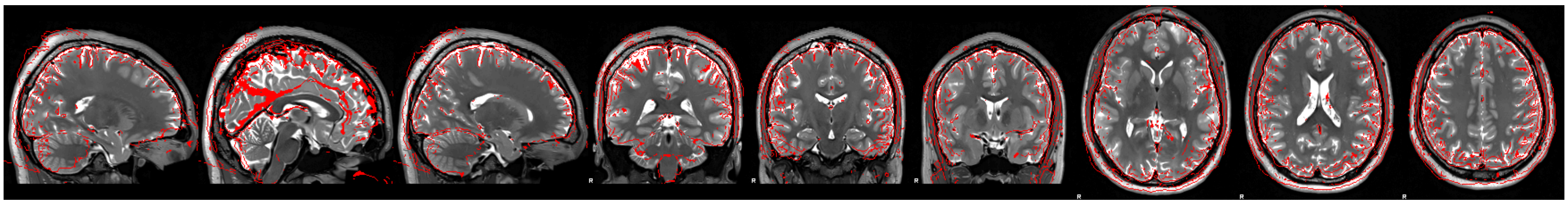

T2w On Task

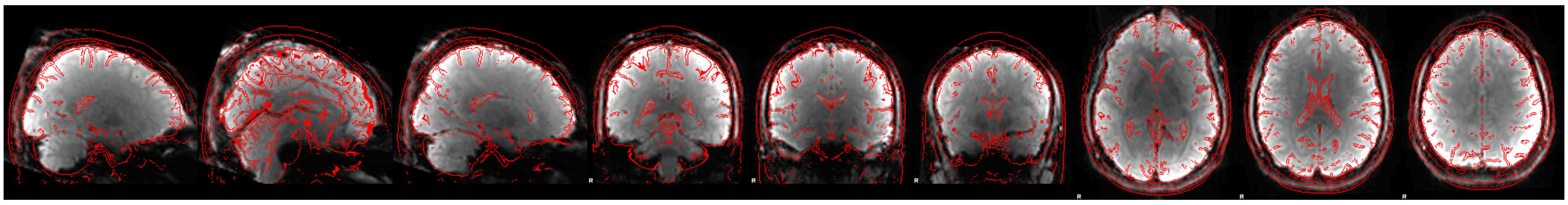

BOLD

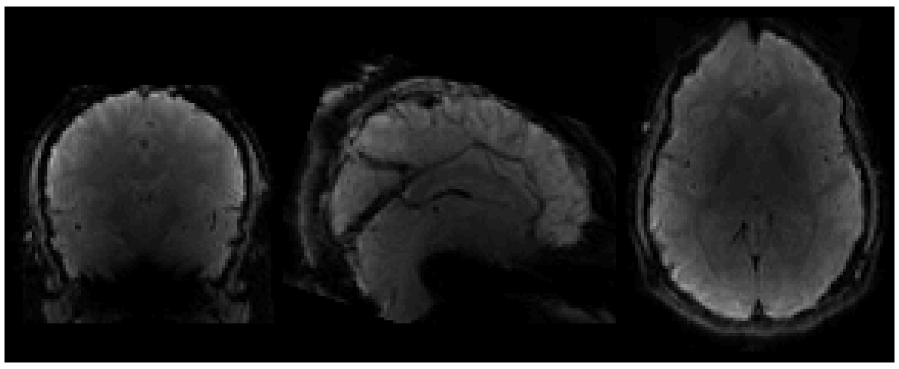

Reference

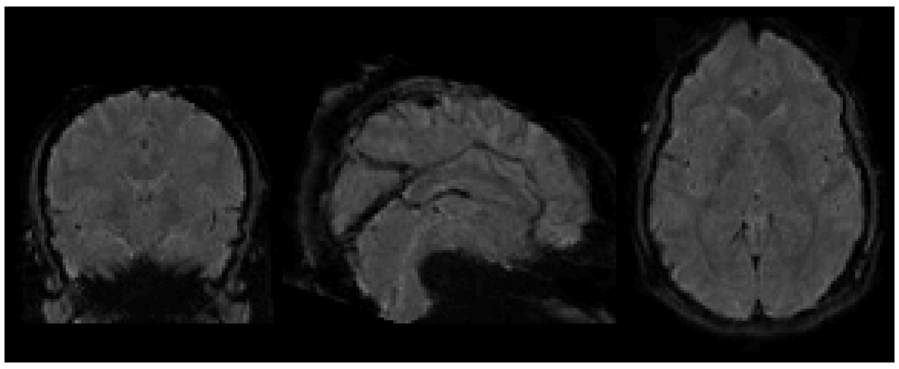

Pre-Regression

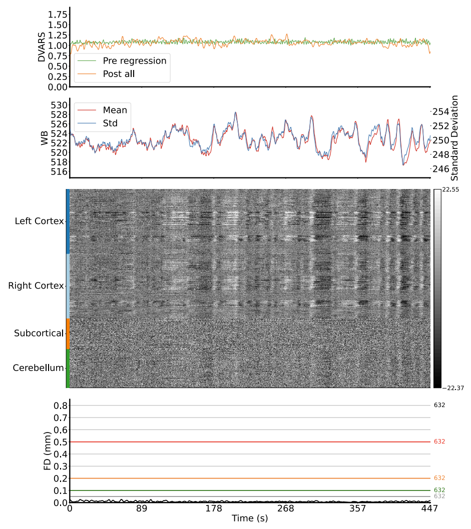

Post-Regression

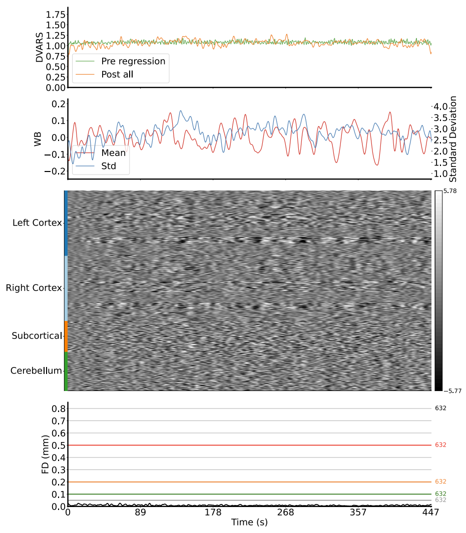

Methods

We kindly ask to report results preprocessed with this tool using the following boilerplate.

HTML Markdown LaTeX

Post-processing of fmriprep outputs

The eXtensible Connectivity Pipeline- DCAN (XCP-D) (Ciric et al. 2018; Satterthwaite et al. 2013) was used to post-process the outputs of *fMRIPrep* version 23.1.3 (Esteban et al. 2019, 2020, RRID:SCR_016216) . XCP-D was built with *Nipype* version 1.8.6 (Gorgolewski et al. 2011, RRID:SCR_002502) . Native-space T1w images were transformed to MNI152NLin6Asym space at 1 mm3 resolution. fsLR-space morphometry surfaces were copied from the preprocessing derivatives to the XCP-D derivatives. HCP-style midthickness, inflated, and very-inflated surfaces were generated from the white-matter and pial surface meshes. fsnative-space surfaces were then warped to fsLR space. For each of the two BOLD runs found per subject (across all tasks and sessions), the following post-processing was performed.

Non-steady-state volumes were extracted from the preprocessed confounds and were discarded from both the BOLD data and nuisance regressors. The six translation and rotation head motion traces were low-pass filtered below 6.0 breaths-per-minute using a(n) fourth-order Butterworth filter, based on Gratton et al. (2020) . The Volterra expansion of these filtered motion parameters was then calculated. Framewise displacement was calculated from the filtered motion parameters using the formula from Power et al. (2014) , with a head radius of 67.98579611765847 mm. Volumes with filtered framewise displacement greater than 0.3 mm were flagged as high-motion outliers for the sake of later censoring (Power et al. 2014) . Additional sets of censoring volumes were randomly selected to produce additional correlation matrices limited to 422 volumes. In total, 36 nuisance regressors were selected from the preprocessing confounds, according to the '36P' strategy. These nuisance regressors included six filtered motion parameters, mean global signal, mean white matter signal, mean cerebrospinal fluid signal with their temporal derivatives, and quadratic expansion of six motion parameters, tissue signals and their temporal derivatives (Ciric et al. 2017; Satterthwaite et al. 2013) . The BOLD data were converted to NIfTI format, despiked with *AFNI* 's *3dDespike* , and converted back to CIFTI format.

Nuisance regressors were regressed from the BOLD data using a denoising method based on *Nilearn* 's approach. Any volumes censored earlier in the workflow were first cubic spline interpolated in the BOLD data. Outlier volumes at the beginning or end of the time series were replaced with the closest low-motion volume's values, as cubic spline interpolation can produce extreme extrapolations. The timeseries were band-pass filtered using a(n) second-order Butterworth filter, in order to retain signals between 0.01-0.1 Hz. The same filter was applied to the confounds. The resulting time series were then denoised via linear regression, in which the low-motion volumes from the BOLD time series and confounds were used to calculate parameter estimates, and then the interpolated time series were denoised using the low-motion parameter estimates. The interpolated time series were then censored using the temporal mask. The denoised BOLD was then smoothed using *Connectome Workbench* with a Gaussian kernel (FWHM=2.0 mm).

The amplitude of low-frequency fluctuation (ALFF) (Zou et al. 2008) was computed by transforming the mean-centered, standard deviation-normalized, denoised BOLD time series to the frequency domain using the Lomb-Scargle periodogram (Lomb 1976; Scargle 1982; Townsend 2010; Taylor et al. 2018) . The power spectrum was computed within the 0.01-0.1 Hz frequency band and the mean square root of the power spectrum was calculated at each voxel to yield voxel-wise ALFF measures. The resulting ALFF values were then multiplied by the standard deviation of the denoised BOLD time series to retain the original scaling. The ALFF maps were smoothed with the Connectome Workbench using a Gaussian kernel (FWHM=2.0 mm).

For each hemisphere, regional homogeneity (ReHo) (Jiang and Zuo 2016) was computed using surface-based *2dReHo* (Zhang et al. 2019) . Specifically, for each vertex on the surface, the Kendall's coefficient of concordance (KCC) was computed with nearest-neighbor vertices to yield ReHo. For the subcortical, volumetric data, ReHo was computed with neighborhood voxels using *AFNI* 's *3dReHo* (Taylor and Saad 2013) .

Processed functional timeseries were extracted from residual BOLD using Connectome Workbench (Marcus et al. 2011) for the following atlases: the Schaefer Supplemented with Subcortical Structures (4S) atlas (Schaefer et al. 2018; Pauli, Nili, and Tyszka 2018; King et al. 2019; Najdenovska et al. 2018; Glasser et al. 2013) at 10 different resolutions (1056, 156, 256, 356, 456, 556, 656, 756, 856, and 956 parcels), the Glasser atlas (Glasser et al. 2016) , the Gordon atlas (Gordon et al. 2016) , the Tian subcortical atlas (Tian et al. 2020) , and the HCP CIFTI subcortical atlas (Glasser et al. 2013) . Corresponding pair-wise functional connectivity between all regions was computed for each atlas, which was operationalized as the Pearson's correlation of each parcel's unsmoothed timeseries with the Connectome Workbench. In cases of partial coverage, uncovered vertices (values of all zeros or NaNs) were either ignored (when the parcel had >50.0% coverage) or were set to zero (when the parcel had <50.0% coverage).

Postprocessing derivatives from multi-run tasks were then concatenated across runs and directions.

Many internal operations of *XCP-D* use *AFNI* (Cox 1996; Cox and Hyde 1997) , *Connectome Workbench* (Marcus et al. 2011) , *ANTS* (Avants et al. 2009) , *TemplateFlow* version 24.2.0 (Ciric et al. 2022) , *matplotlib* version 3.8.4 (Hunter 2007) , *Nibabel* version 5.2.1 (Brett et al. 2022) , *Nilearn* version 0.10.4 (Abraham et al. 2014) , *numpy* version 1.26.4 (Harris et al. 2020) , *pybids* version 0.16.4 (Yarkoni et al. 2019) , and *scipy* version 1.13.0 (Virtanen et al. 2020) . For more details, see the *XCP-D* website (https://xcp-d.readthedocs.io).

Copyright Waiver

The above methods description text was automatically generated by *XCP-D* with the express intention that users should copy and paste this text into their manuscripts *unchanged* . It is released under the CC0 license.

References

Abraham, Alexandre, Fabian Pedregosa, Michael Eickenberg, Philippe Gervais, Andreas Mueller, Jean Kossaifi, Alexandre Gramfort, Bertrand Thirion, and Gaël Varoquaux. 2014. "Machine Learning for Neuroimaging with Scikit-Learn." *Frontiers in Neuroinformatics* . Frontiers, 14.

Avants, Brian B, Nick Tustison, Gang Song, and others. 2009. "Advanced Normalization Tools (Ants)." *Insight J* 2 (365): 1–35.

Brett, Matthew, Christopher J. Markiewicz, Michael Hanke, Marc-Alexandre Côté, Ben Cipollini, Paul McCarthy, Dorota Jarecka, et al. 2022. *Nipy/Nibabel:* (version 4.0.0). Zenodo. https://doi.org/10.5281/zenodo.591597 .

Ciric, Rastko, Adon F. G. Rosen, Guray Erus, Matthew Cieslak, Azeez Adebimpe, Philip A. Cook, Danielle S. Bassett, Christos Davatzikos, Daniel H. Wolf, and Theodore D. Satterthwaite. 2018. "Mitigating Head Motion Artifact in Functional Connectivity MRI." *Nature Protocols* 13 (12): 2801–26. https://doi.org/10.1038/s41596-018-0065-y .

Ciric, Rastko, William H Thompson, Romy Lorenz, Mathias Goncalves, Eilidh MacNicol, Christopher J Markiewicz, Yaroslav O Halchenko, et al. 2022. "TemplateFlow: FAIR-Sharing of Multi-Scale, Multi-Species Brain Models." *bioRxiv* . Cold Spring Harbor Laboratory, 2021–02. https://doi.org/10.1101/2021.02.10.430678 .

Ciric, Rastko, Daniel H. Wolf, Jonathan D. Power, David R. Roalf, Graham Baum, Kosha Ruparel, Russell T. Shinohara, et al. 2017. "Benchmarking of Participant-Level Confound Regression Strategies for the Control of Motion Artifact in Studies of Functional Connectivity." *NeuroImage* 154 (July): 174–87. https://doi.org/10.1016/j.neuroimage.2017.03.020 .

Cox, Robert W. 1996. "AFNI: Software for Analysis and Visualization of Functional Magnetic Resonance Neuroimages." *Computers and Biomedical Research* 29 (3). Elsevier: 162–73.

Cox, Robert W, and James S Hyde. 1997. "Software Tools for Analysis and Visualization of fMRI Data." *NMR in Biomedicine: An International Journal Devoted to the Development and Application of Magnetic Resonance in Vivo* 10 (4-5). Wiley Online Library: 171–78.

Esteban, Oscar, Rastko Ciric, Karolina Finc, Ross W Blair, Christopher J Markiewicz, Craig A Moodie, James D Kent, et al. 2020. "Analysis of Task-Based Functional Mri Data Preprocessed with fMRIPrep." *Nature Protocols* 15 (7). Nature Publishing Group: 2186–2202. https://doi.org/10.1038/s41596-020-0327-3 .

Esteban, Oscar, Christopher J Markiewicz, Ross W Blair, Craig A Moodie, A Ilkay Isik, Asier Erramuzpe, James D Kent, et al. 2019. "FMRIPrep: A Robust Preprocessing Pipeline for Functional Mri." *Nature Methods* 16 (1). Nature Publishing Group: 111–16. https://doi.org/10.1038/s41592-018-0235-4 .

Glasser, Matthew F., Timothy S. Coalson, Emma C. Robinson, Carl D. Hacker, John Harwell, Essa Yacoub, Kamil Ugurbil, et al. 2016. "A Multi-Modal Parcellation of Human Cerebral Cortex." *Nature* 536 (7615): 171–78. https://doi.org/10.1038/nature18933 .

Glasser, Matthew F., Stamatios N. Sotiropoulos, J. Anthony Wilson, Timothy S. Coalson, Bruce Fischl, Jesper L. Andersson, Junqian Xu, et al. 2013. "The Minimal Preprocessing Pipelines for the Human Connectome Project." *NeuroImage* 80 (October): 105–24. https://doi.org/10.1016/j.neuroimage.2013.04.127 .

Gordon, Evan M., Timothy O. Laumann, Babatunde Adeyemo, Jeremy F. Huckins, William M. Kelley, and Steven E. Petersen. 2016. "Generation and Evaluation of a Cortical Area Parcellation from Resting-State Correlations." *Cerebral Cortex* 26 (1): 288–303. https://doi.org/10.1093/cercor/bhu239 .

Gorgolewski, Krzysztof, Christopher D. Burns, Cindee Madison, Dav Clark, Yaroslav O. Halchenko, Michael L. Waskom, and Satrajit S. Ghosh. 2011. "Nipype: A Flexible, Lightweight and Extensible Neuroimaging Data Processing Framework in Python." *Frontiers in Neuroinformatics* 5. https://doi.org/10.3389/fninf.2011.00013 .

Gratton, Caterina, Ally Dworetsky, Rebecca S Coalson, Babatunde Adeyemo, Timothy O Laumann, Gagan S Wig, Tania S Kong, et al. 2020. "Removal of High Frequency Contamination from Motion Estimates in Single-Band fMRI Saves Data Without Biasing Functional Connectivity." *Neuroimage* 217. Elsevier: 116866. https://doi.org/10.1016/j.neuroimage.2020.116866 .

Harris, Charles R., Jarrod K. Millman, Stéfan J. van der Walt, Ralf Gommers, Pauli Virtanen, David Cournapeau, Eric Wieser, et al. 2020. "Array Programming with NumPy." *Nature* 585 (7825): 357–62. https://doi.org/10.1038/s41586-020-2649-2 .

Hunter, John D. 2007. "Matplotlib: A 2D Graphics Environment." *Computing in Science & Engineering* 9 (03). IEEE Computer Society: 90–95.

Jiang, Lili, and Xi-Nian Zuo. 2016. "Regional Homogeneity: A Multimodal, Multiscale Neuroimaging Marker of the Human Connectome." *The Neuroscientist* 22 (5). Sage Publications Sage CA: Los Angeles, CA: 486–505.

King, Maedbh, Carlos R Hernandez-Castillo, Russell A Poldrack, Richard B Ivry, and Jörn Diedrichsen. 2019. "Functional Boundaries in the Human Cerebellum Revealed by a Multi-Domain Task Battery." *Nature Neuroscience* 22 (8). Nature Publishing Group US New York: 1371–8. https://doi.org/10.1038/s41593-019-0436-x .

Lomb, Nicholas R. 1976. "Least-Squares Frequency Analysis of Unequally Spaced Data." *Astrophysics and Space Science* 39. Springer: 447–62.

Marcus, Daniel S, John Harwell, Timothy Olsen, Michael Hodge, Matthew F Glasser, Fred Prior, Mark Jenkinson, Timothy Laumann, Sandra W Curtiss, and David C Van Essen. 2011. “Informatics and Data Mining Tools and Strategies for the Human Connectome Project.” *Frontiers in Neuroinformatics* 5. Frontiers Research Foundation: 4.

Najdenovska, Elena, Yasser Alemán-Gómez, Giovanni Battistella, Maxime Descoteaux, Patric Hagmann, Sebastien Jacquemont, Philippe Maeder, Jean-Philippe Thiran, Eleonora Fornari, and Meritxell Bach Cuadra. 2018. “In-Vivo Probabilistic Atlas of Human Thalamic Nuclei Based on Diffusion-Weighted Magnetic Resonance Imaging.” *Scientific Data* 5 (1). Nature Publishing Group: 1–11. https://doi.org/10.1038/sdata.2018.270 .

Pauli, Wolfgang M, Amanda N Nili, and J Michael Tyszka. 2018. “A High-Resolution Probabilistic in Vivo Atlas of Human Subcortical Brain Nuclei.” *Scientific Data* 5 (1). Nature Publishing Group: 1–13. https://doi.org/10.1038/sdata.2018.63 .

Power, Jonathan D., Anish Mitra, Timothy O. Laumann, Abraham Z. Snyder, Bradley L. Schlaggar, and Steven E. Petersen. 2014. “Methods to Detect, Characterize, and Remove Motion Artifact in Resting State fMRI.” *NeuroImage* 84 (January): 320–41. https://doi.org/10.1016/j.neuroimage.2013.08.048 .

Satterthwaite, Theodore D., Mark A. Elliott, Raphael T. Gerraty, Kosha Ruparel, James Loughead, Monica E. Calkins, Simon B. Eickhoff, et al. 2013. “An Improved Framework for Confound Regression and Filtering for Control of Motion Artifact in the Preprocessing of Resting-State Functional Connectivity Data.” *NeuroImage* 64 (January): 240–56. https://doi.org/10.1016/j.neuroimage.2012.08.052 .

Scargle, Jeffrey D. 1982. “Studies in Astronomical Time Series Analysis. II-Statistical Aspects of Spectral Analysis of Unevenly Spaced Data.” *Astrophysical Journal, Part 1, Vol. 263, Dec. 15, 1982, P. 835-853.* 263: 835–53.

Schaefer, Alexander, Ru Kong, Evan M. Gordon, Timothy O. Laumann, Xi-Nian Zuo, Avram J. Holmes, Simon B. Eickhoff, and B. T. Thomas Yeo. 2018. “Local-Global Parcellation of the Human Cerebral Cortex from Intrinsic Functional Connectivity MRI.” *Cerebral Cortex (New York, N.Y.: 1991)* 28 (9): 3095–3114. https://doi.org/10.1093/cercor/bhx179 .

Taylor, Paul A, Gang Chen, Daniel R Glen, Richard C Reynolds, and Robert W Cox. 2018. “Lomb-Scargle Your Way to Rsfc Parameter Estimation in Afni-Fatcat.” *International Society for Magnetic Resonance in Medicine* . International Society for Magnetic Resonance in Medicine.

Taylor, Paul A, and Ziad S Saad. 2013. “FATCAT:(An Efficient) Functional and Tractographic Connectivity Analysis Toolbox.” *Brain Connectivity* 3 (5). Mary Ann Liebert, Inc. 140 Huguenot Street, 3rd Floor New Rochelle, NY 10801 USA: 523–35.

Tian, Ye, Daniel S Margulies, Michael Breakspear, and Andrew Zalesky. 2020. “Topographic Organization of the Human Subcortex Unveiled with Functional Connectivity Gradients.” *Nature Neuroscience* 23 (11). Nature Publishing Group: 1421–32. https://doi.org/10.1038/s41593-020-00711-6 .

Townsend, RHD. 2010. “Fast Calculation of the Lomb–Scargle Periodogram Using Graphics Processing Units.” *The Astrophysical Journal Supplement Series* 191 (2). IOP Publishing: 247.

Virtanen, Pauli, Ralf Gommers, Travis E. Oliphant, Matt Haberland, Tyler Reddy, David Cournapeau, Evgeni Burovski, et al. 2020. “SciPy 1.0: Fundamental Algorithms for Scientific Computing in Python.” *Nature Methods* 17 (3): 261–72. https://doi.org/10.1038/s41592-019-0686-2 .

Yarkoni, Tal, Christopher J Markiewicz, Alejandro de la Vega, Krzysztof J Gorgolewski, Taylor Salo, Yaroslav O Halchenko, Quinten McNamara, et al. 2019. “PyBIDS: Python Tools for Bids Datasets.” *Journal of Open Source Software* 4 (40). NIH Public Access.

Zhang, Bo, Fei Wang, Hao-Ming Dong, Xiao-Wei Jiang, Sheng-Nan Wei, Miao Chang, Zhi-Yang Yin, et al. 2019. “Surface-Based Regional Homogeneity in Bipolar Disorder: A Resting-State fMRI Study.” *Psychiatry Research* 278 (August): 199–204. https://doi.org/10.1016/j.psychres.2019.05.045 .

Zou, Qi-Hong, Chao-Zhe Zhu, Yihong Yang, Xi-Nian Zuo, Xiang-Yu Long, Qing-Jiu Cao, Yu-Feng Wang, and Yu-Feng Zang. 2008. “An Improved Approach to Detection of Amplitude of Low-Frequency Fluctuation (ALFF) for Resting-State fMRI: Fractional ALFF.” *Journal of Neuroscience Methods* 172 (1): 137–41. https://doi.org/10.1016/j.jneumeth.2008.04.012 .

Summary

- Subject ID: 01
- BOLD series: 2

Processing Summary

Reports for: task rest.

Summary

- BOLD volume space: fsLR
- Repetition Time (TR): 0.71
- Mean Framewise Displacement: 0.0085
- Mean Relative RMS Motion: 0.0797
- Max Relative RMS Motion: 0.2486
- DVARS Before and After Processing : 0.8269, 1.0908
- Correlation between DVARS and FD Before and After Processing : 0.957, 0.6575
- Number of Volumes Censored : 0

Carpet Plot Before Postprocessing

FD and DVARS are two measures of in-scanner motion. This plot shows standardized FD, DVARS, and then a carpet plot for the time series of each voxel/vertex's time series of activity.

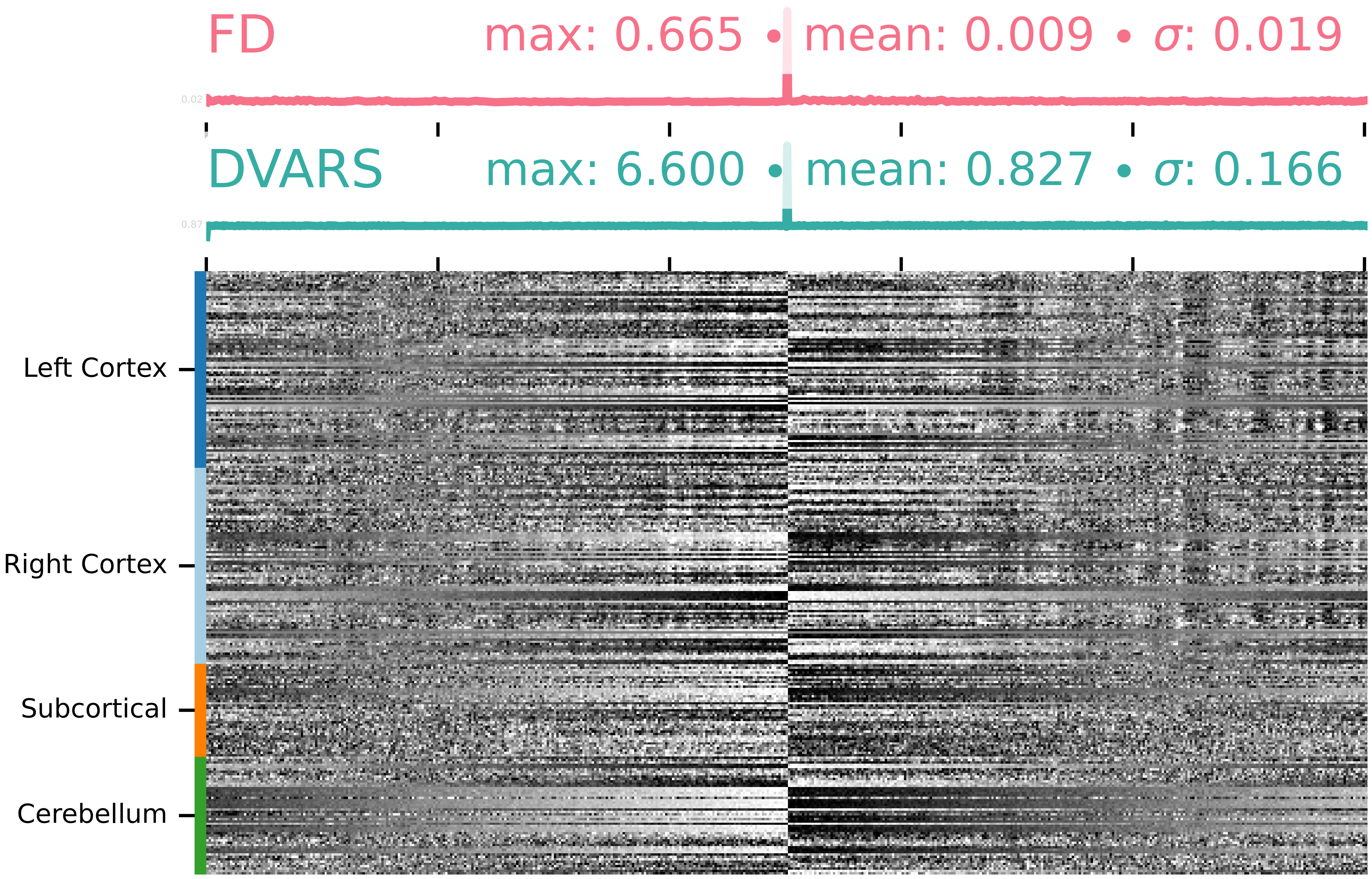

Get figure file: sub-01/figures/sub-01_task-rest_space-fsLR_desc-preprocessing_bold.svg

Carpet Plot After Postprocessing

FD and DVARS are two measures of in-scanner motion. This plot shows standardized FD, DVARS, and then a carpet plot for the time series of each voxel/vertex's time series of activity.

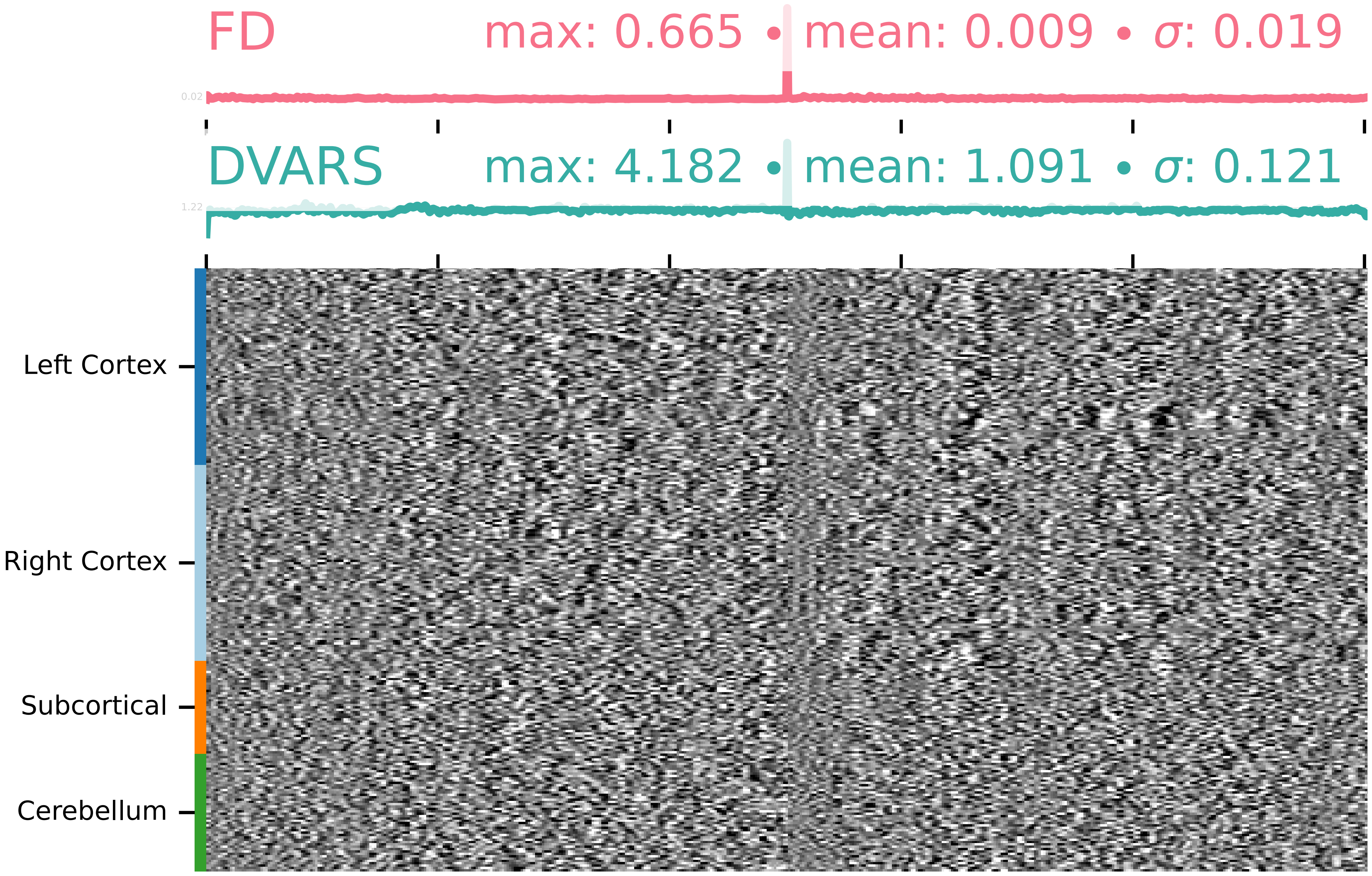

Get figure file: sub-01/figures/sub-01_task-rest_space-fsLR_desc-postprocessing_bold.svg

Reports for: task rest, run 01.

Summary

- BOLD volume space: fsLR
- Repetition Time (TR): 0.71
- Mean Framewise Displacement: 0.0067
- Mean Relative RMS Motion: 0.0765
- Max Relative RMS Motion: 0.2486
- DVARS Before and After Processing : 1.0694, 1.0797
- Correlation between DVARS and FD Before and After Processing : 0.0898, -0.2367
- Number of Volumes Censored : 0

Alignment of functional and anatomical MRI data (surface driven)

bbregister was used to coregister functional and anatomical MRI data.

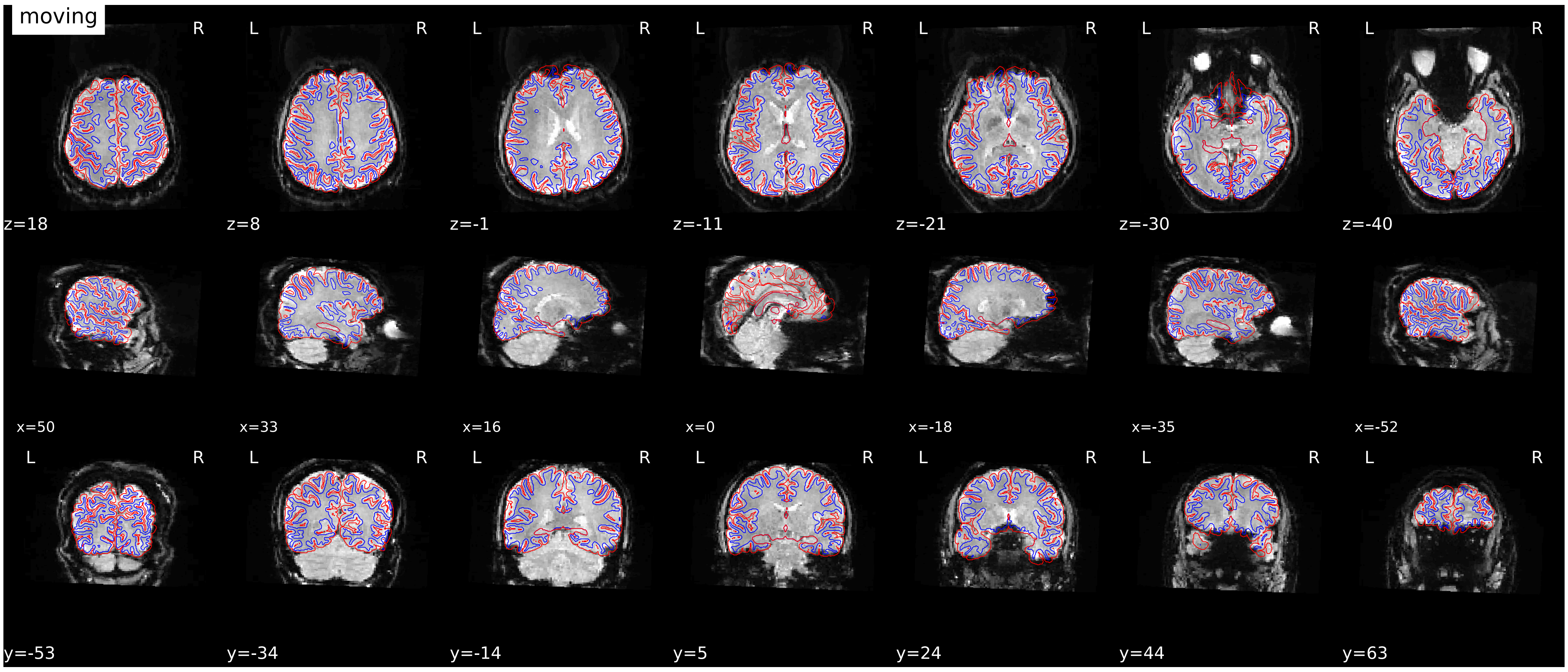

Get figure file: sub-01/figures/sub-01_task-rest_run-01_space-MNI152NLin6Asym_desc-bbregister_bold.svg

Carpet Plot Before Postprocessing

FD and DVARS are two measures of in-scanner motion. This plot shows standardized FD, DVARS, and then a carpet plot for the time series of each voxel/vertex's time series of activity.

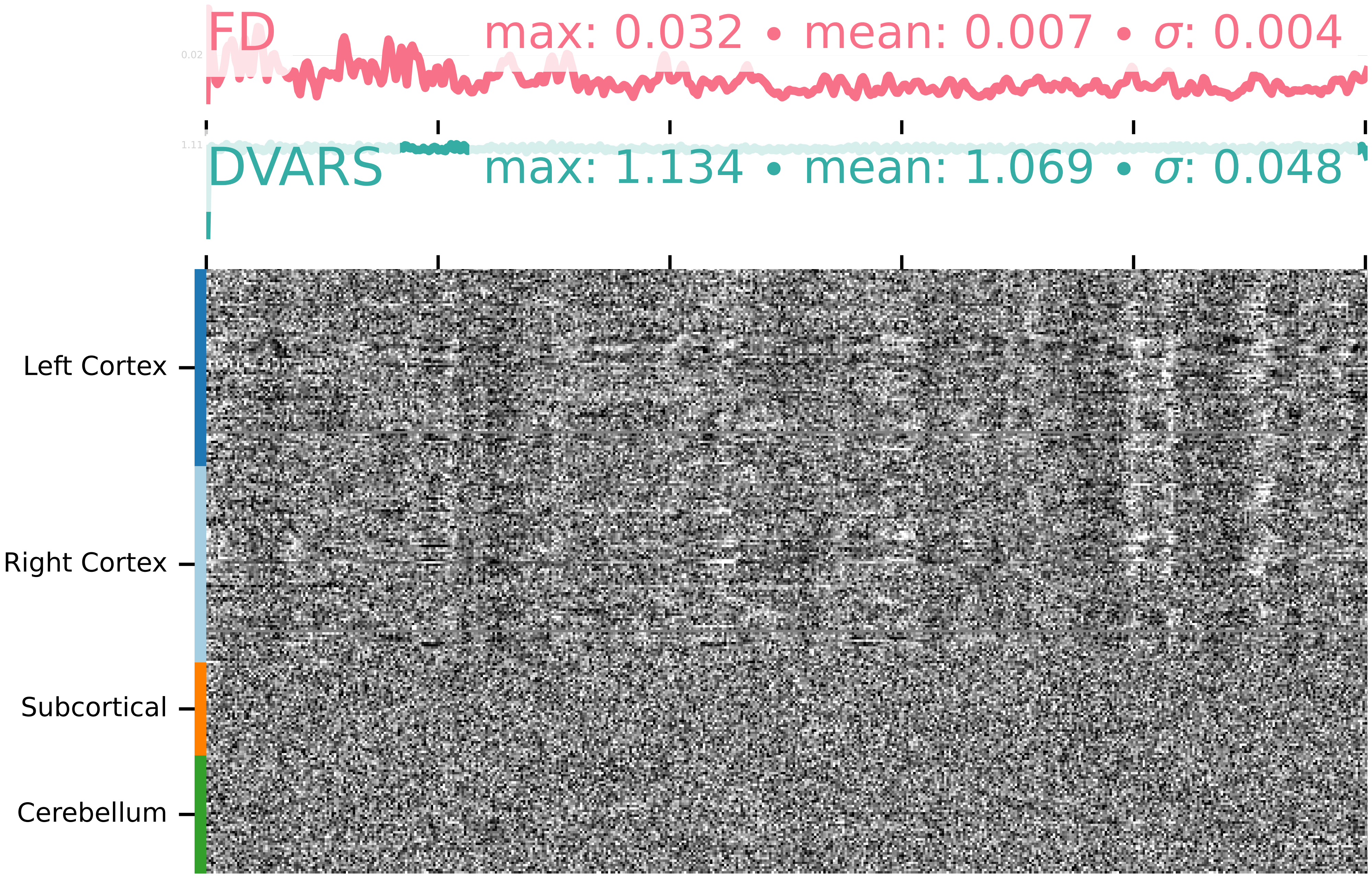

Get figure file: sub-01/figures/sub-01_task-rest_run-01_space-fsLR_desc-preprocessing_bold.svg

Framewise Displacement and Censored Volumes

Framewise displacement (FD) is used to flag high-motion volumes, which are then censored as part of the denoising procedure. If motion filtering is requested, then the six translation and rotation motion parameters are filtered to remove respiratory effects before FD is calculated and outlier volumes are identified.

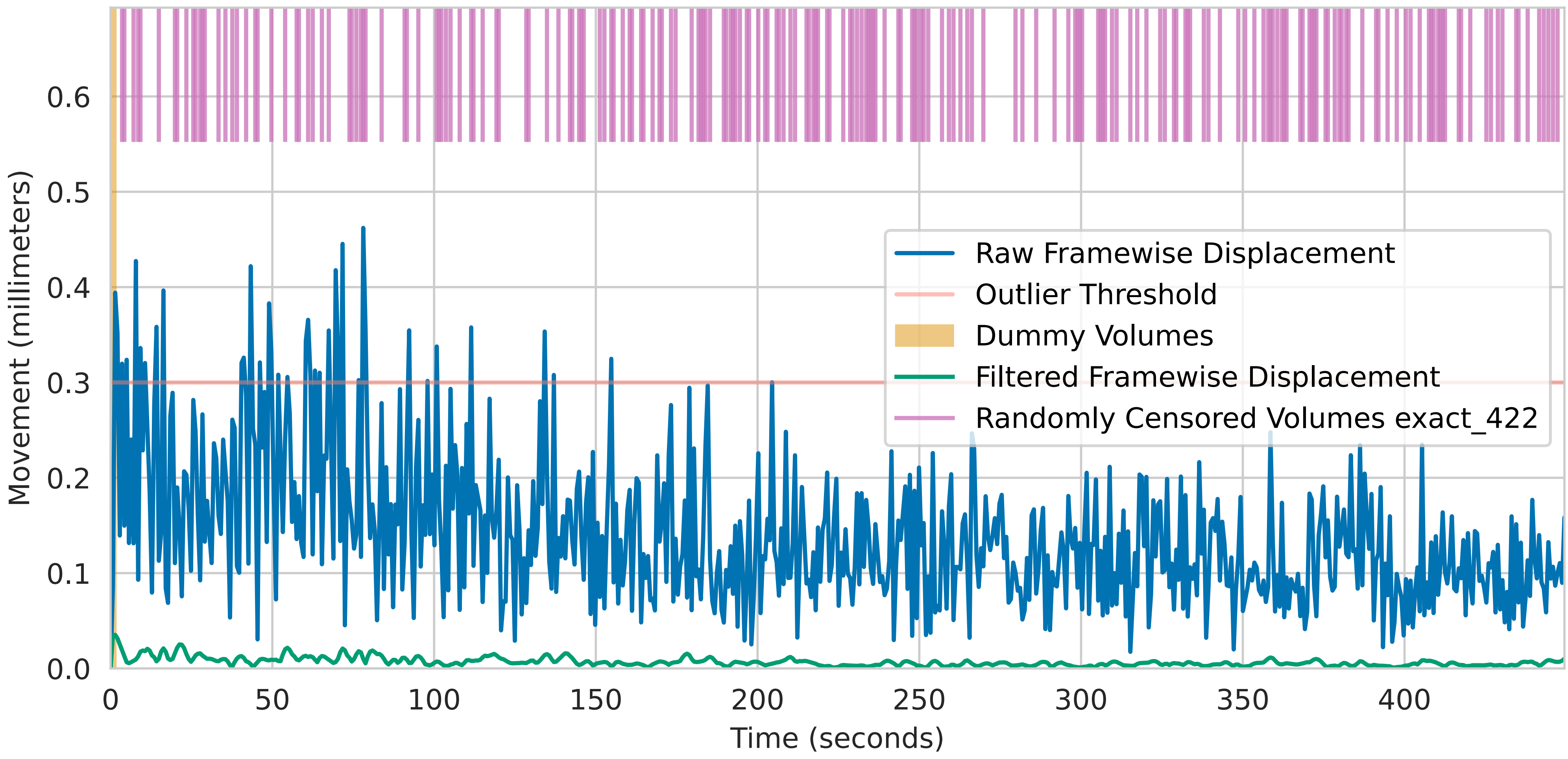

Get figure file: sub-01/figures/sub-01_task-rest_run-01_space-fsLR_desc-censoring_motion.svg

Design Matrix for Confound Regression

The "design matrix" represents the confounds that are used to denoise the BOLD data.

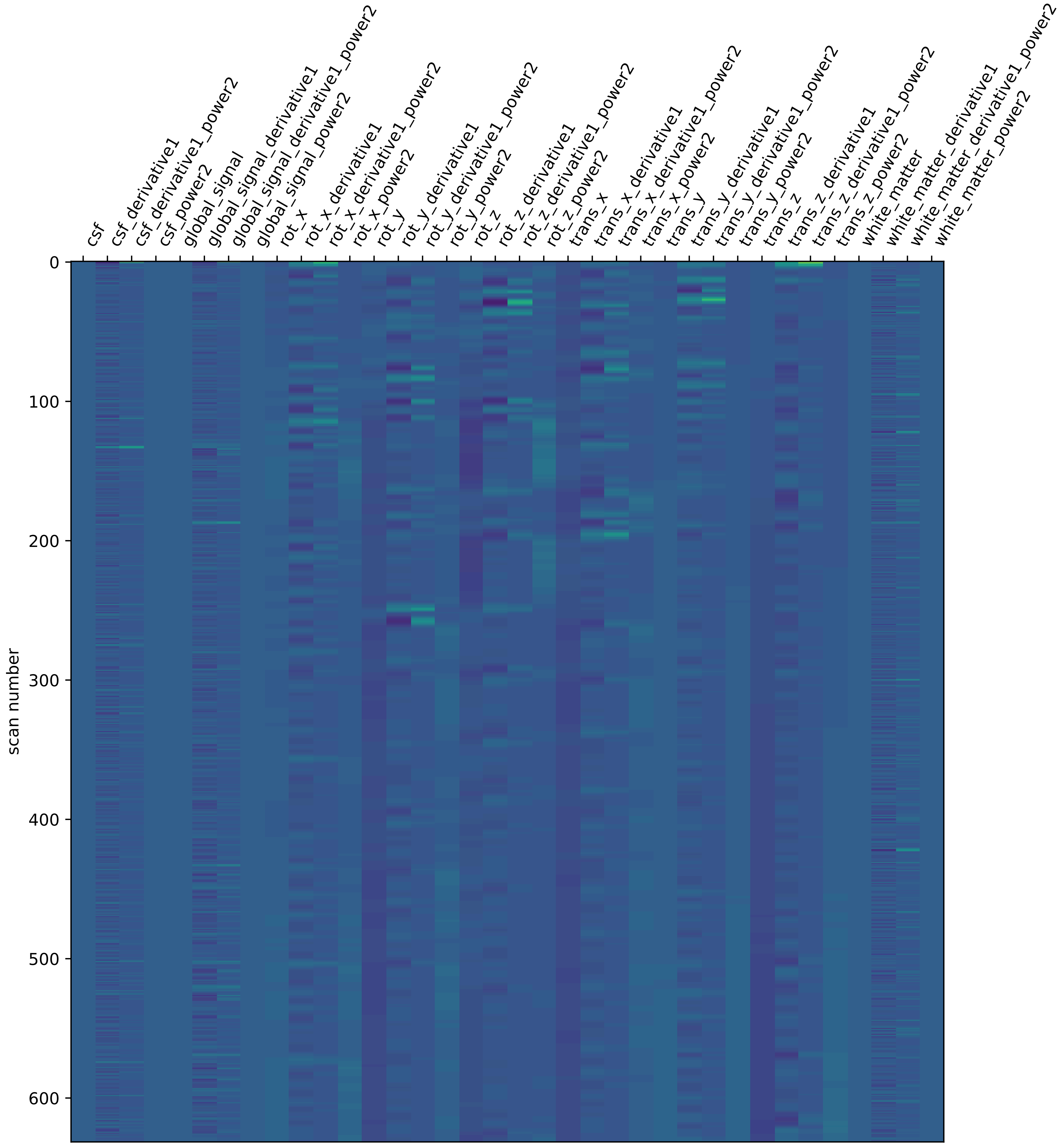

Get figure file: sub-01/figures/sub-01_task-rest_run-01_design.svg

Carpet Plot After Postprocessing

FD and DVARS are two measures of in-scanner motion. This plot shows standardized FD, DVARS, and then a carpet plot for the time series of each voxel/vertex's time series of activity.

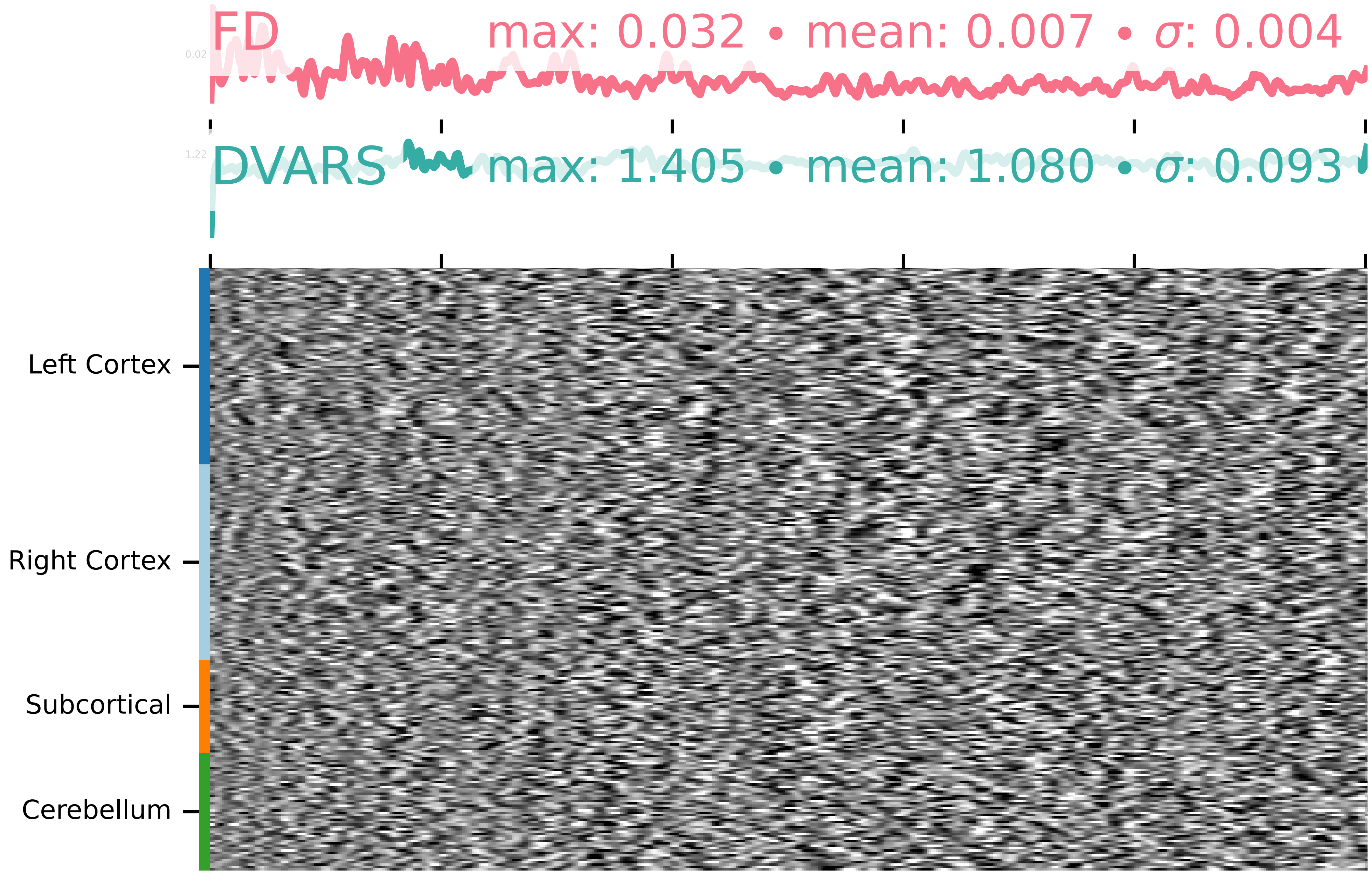

Get figure file: sub-01/figures/sub-01_task-rest_run-01_space-fsLR_desc-postprocessing_bold.svg

Correlation Heatmaps from Four Atlases

This plot shows heatmaps from ROI-to-ROI correlations from four atlases.

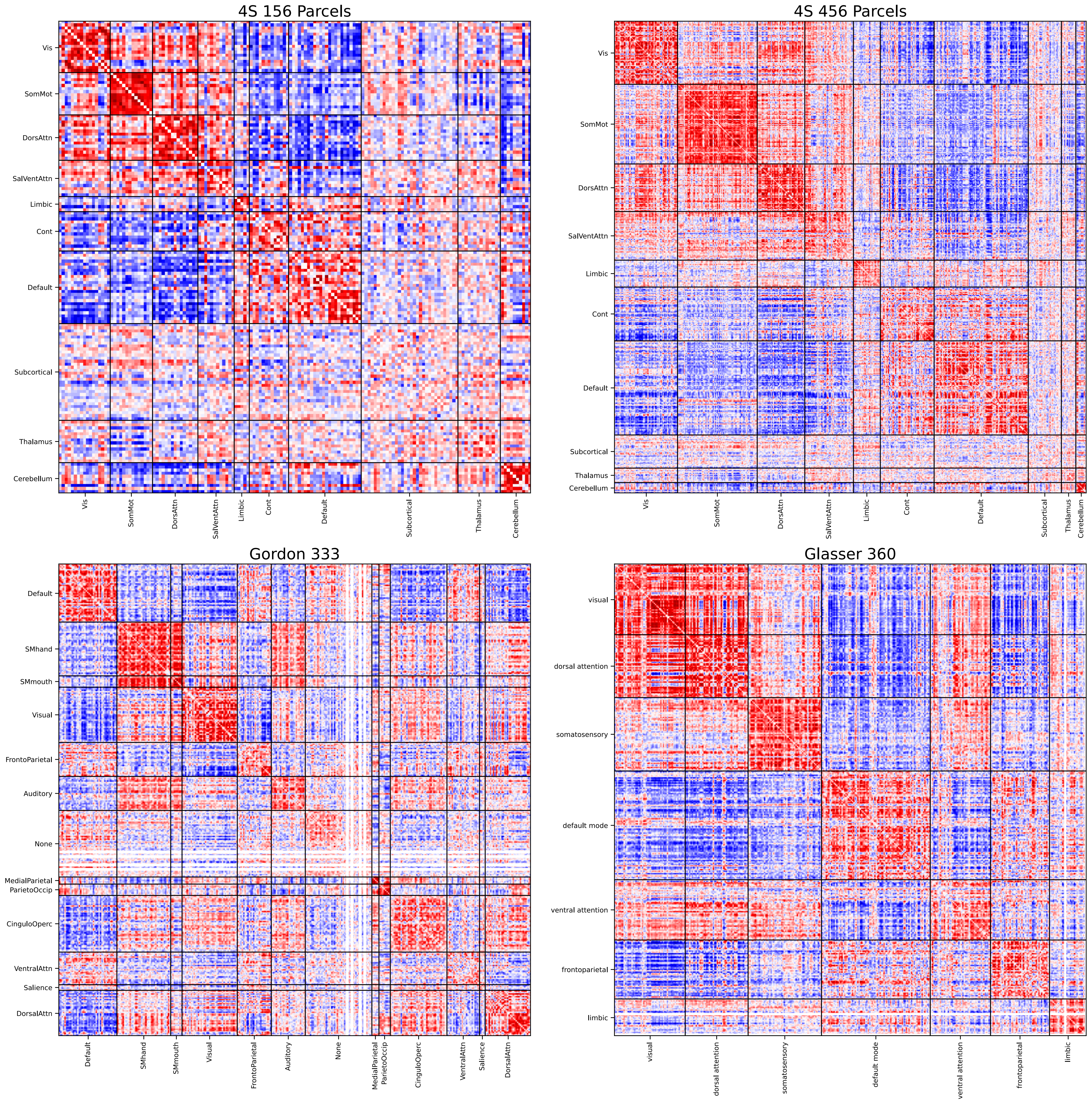

Get figure file: sub-01/figures/sub-01_task-rest_run-01_space-fsLR_desc-connectivityplot_bold.svg

Reports for: task rest, run 02.

Summary

- BOLD volume space: fsLR
- Repetition Time (TR): 0.71
- Mean Framewise Displacement: 0.0093
- Mean Relative RMS Motion: 0.0828
- Max Relative RMS Motion: 0.2131
- DVARS Before and After Processing : 1.0808, 1.0603
- Correlation between DVARS and FD Before and After Processing : 0.0148, -0.179
- Number of Volumes Censored : 0

Alignment of functional and anatomical MRI data (surface driven)

bbregister was used to coregister functional and anatomical MRI data.

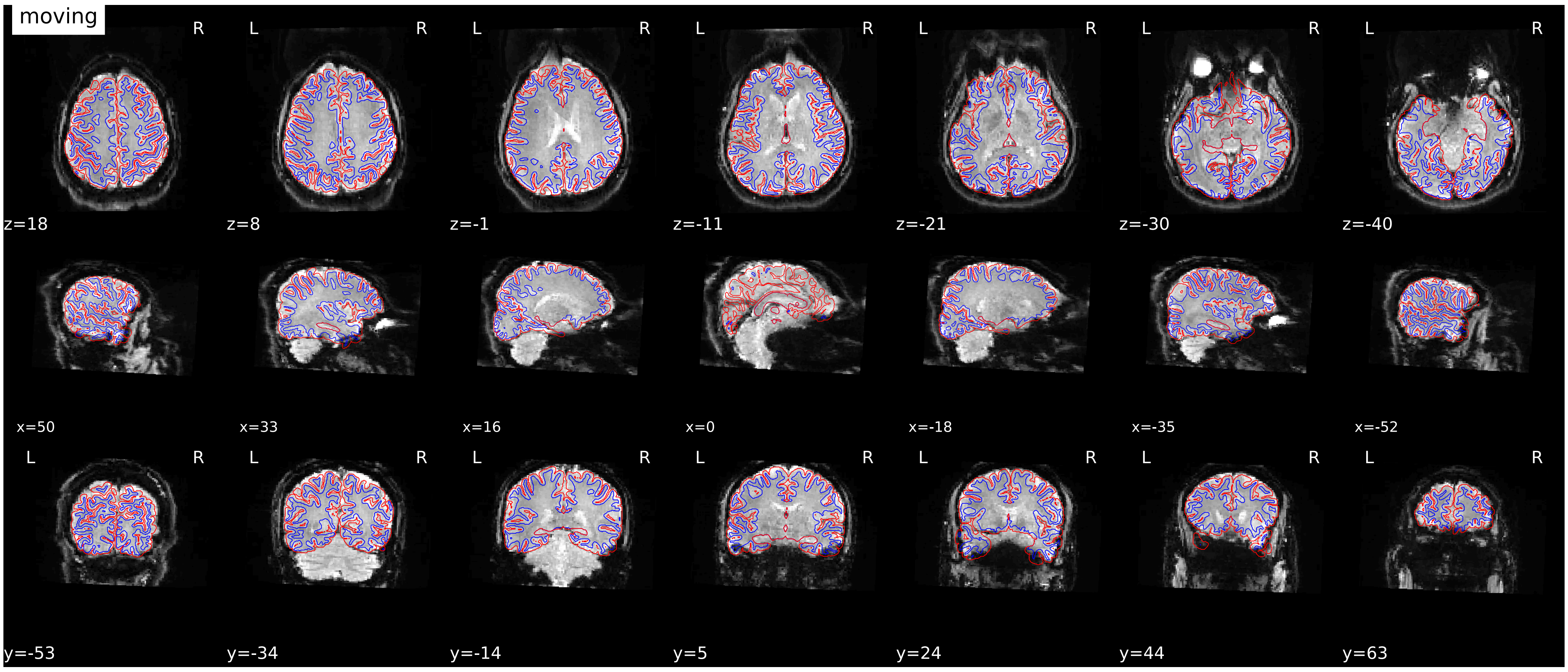

Get figure file: sub-01/figures/sub-01_task-rest_run-02_space-MNI152NLin6Asym_desc-bbregister_bold.svg

Carpet Plot Before Postprocessing

FD and DVARS are two measures of in-scanner motion. This plot shows standardized FD, DVARS, and then a carpet plot for the time series of each voxel/vertex's time series of activity.

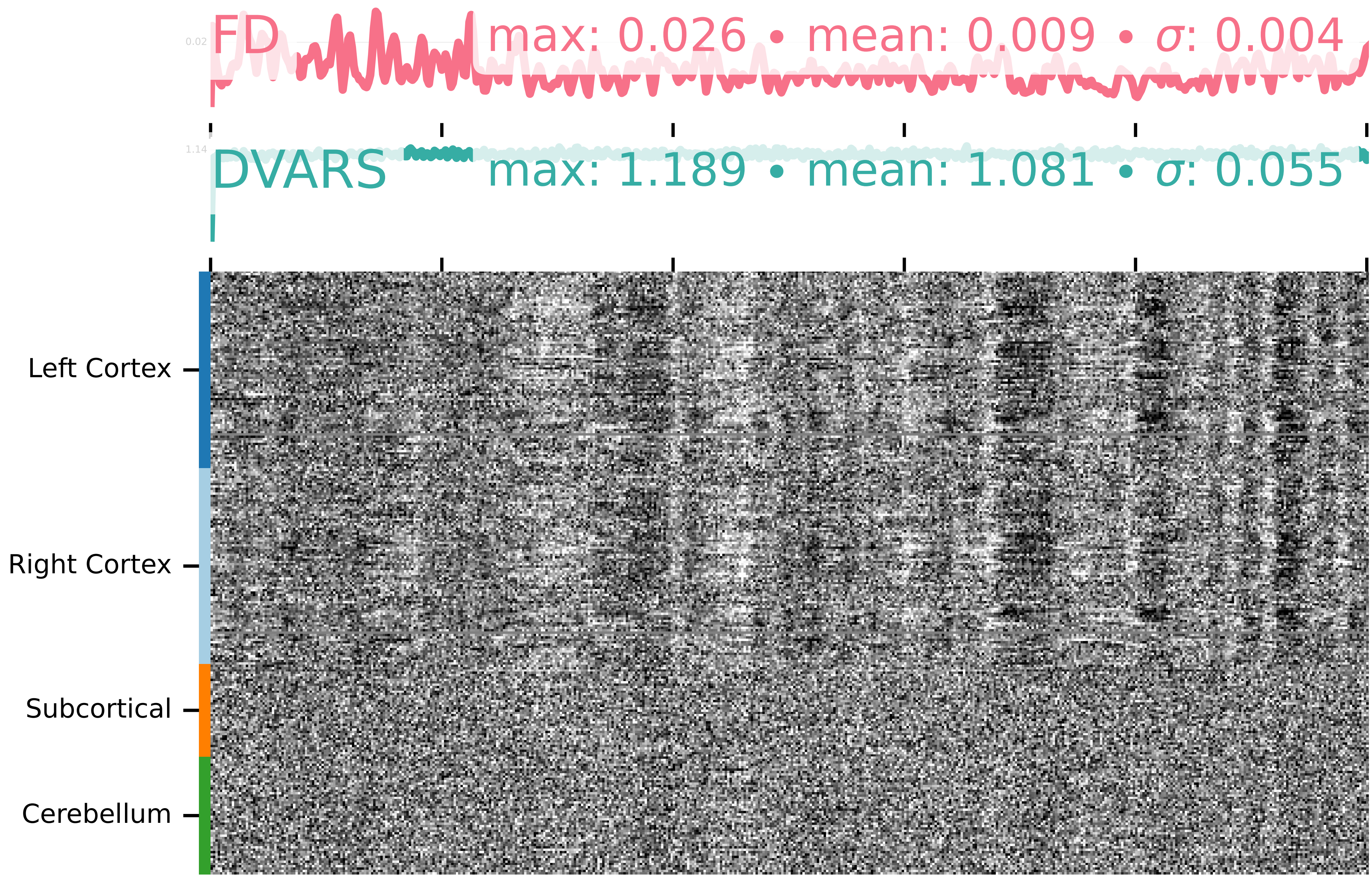

Get figure file: sub-01/figures/sub-01_task-rest_run-02_space-fsLR_desc-preprocessing_bold.svg

Framewise Displacement and Censored Volumes

Framewise displacement (FD) is used to flag high-motion volumes, which are then censored as part of the denoising procedure. If motion filtering is requested, then the six translation and rotation motion parameters are filtered to remove respiratory effects before FD is calculated and outlier volumes are identified.

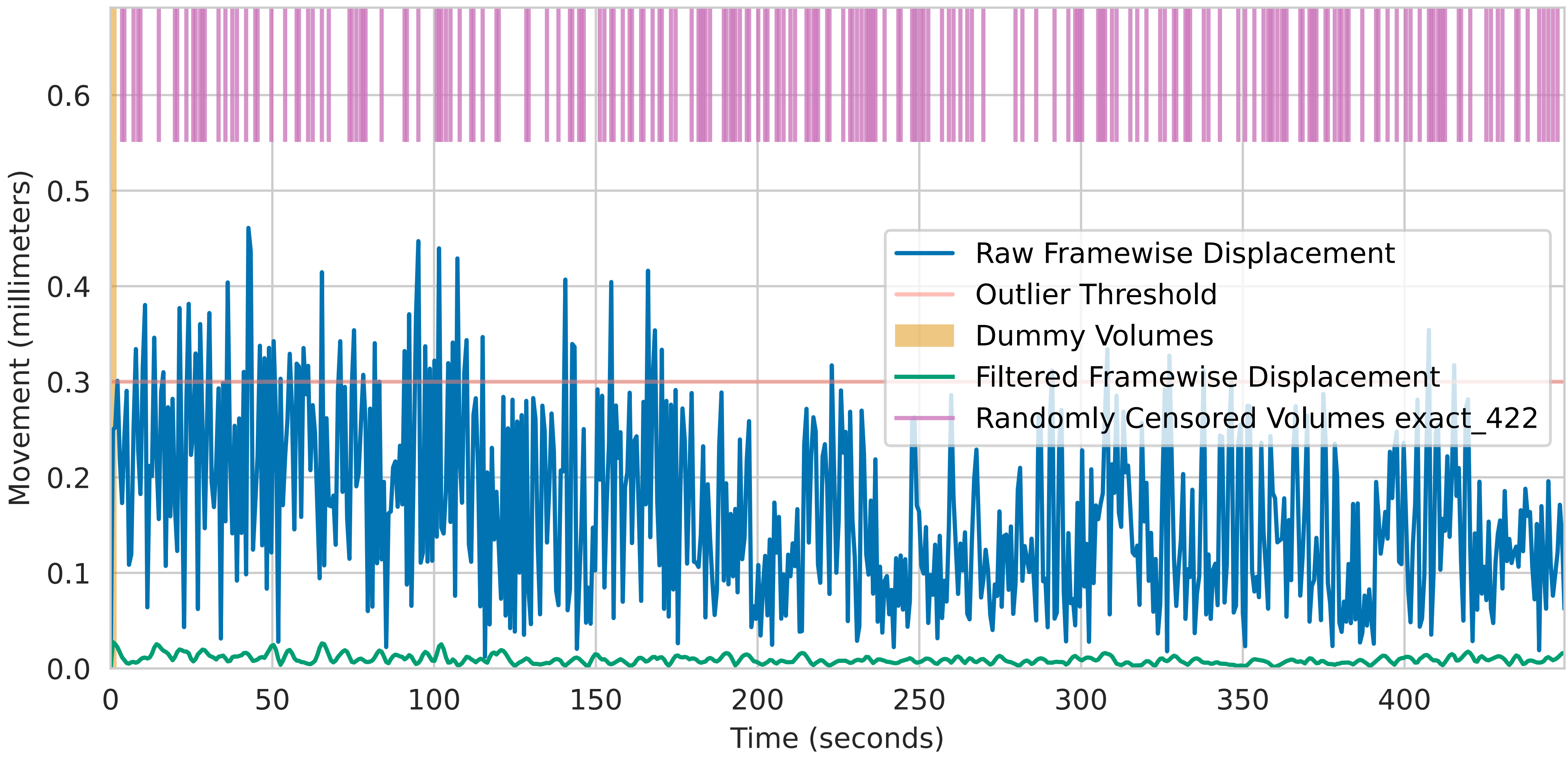

Get figure file: sub-01/figures/sub-01_task-rest_run-02_space-fsLR_desc-censoring_motion.svg

Design Matrix for Confound Regression

The "design matrix" represents the confounds that are used to denoise the BOLD data.

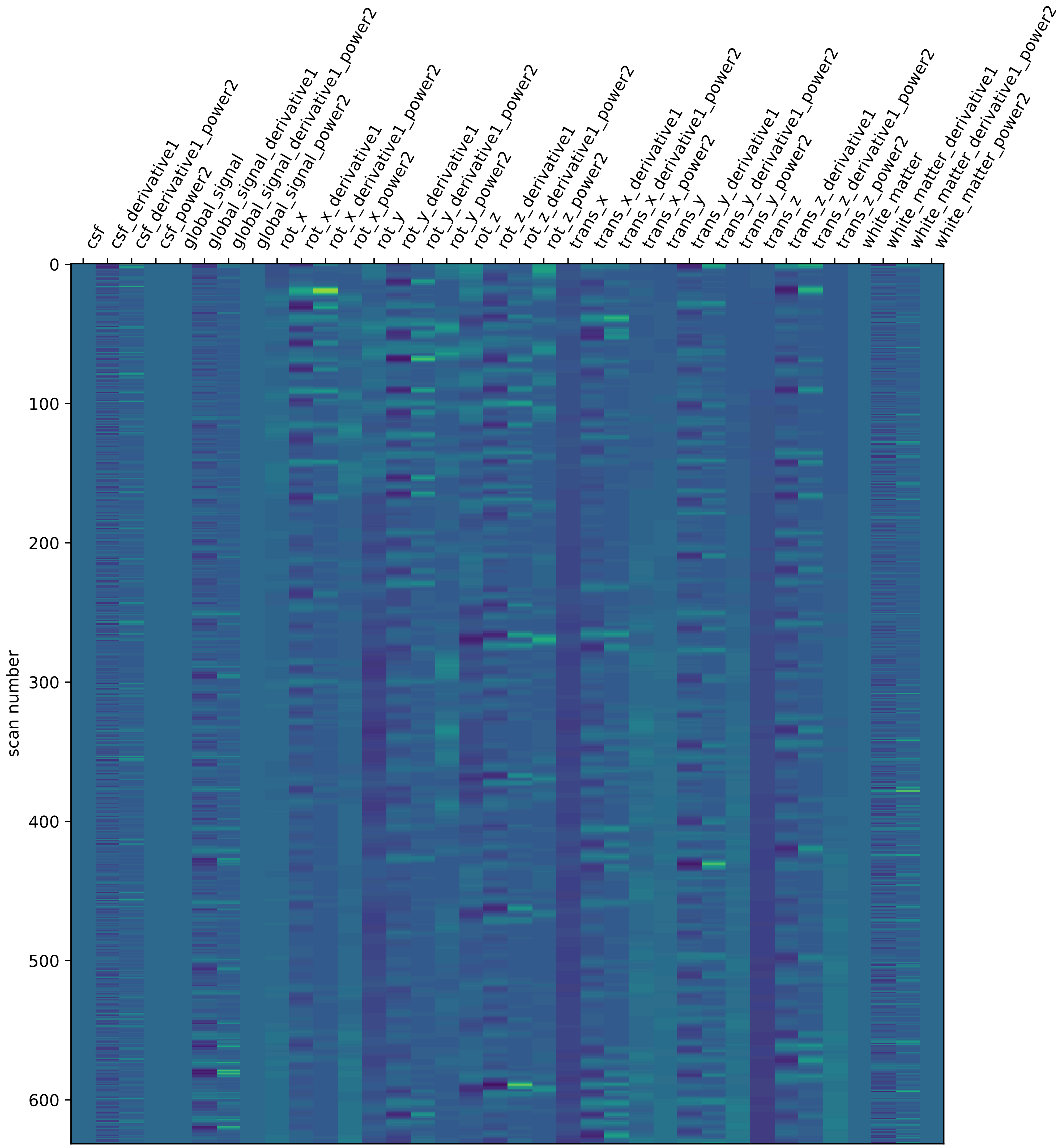

Get figure file: sub-01/figures/sub-01_task-rest_run-02_design.svg

Carpet Plot After Postprocessing

FD and DVARS are two measures of in-scanner motion. This plot shows standardized FD, DVARS, and then a carpet plot for the time series of each voxel/vertex's time series of activity.

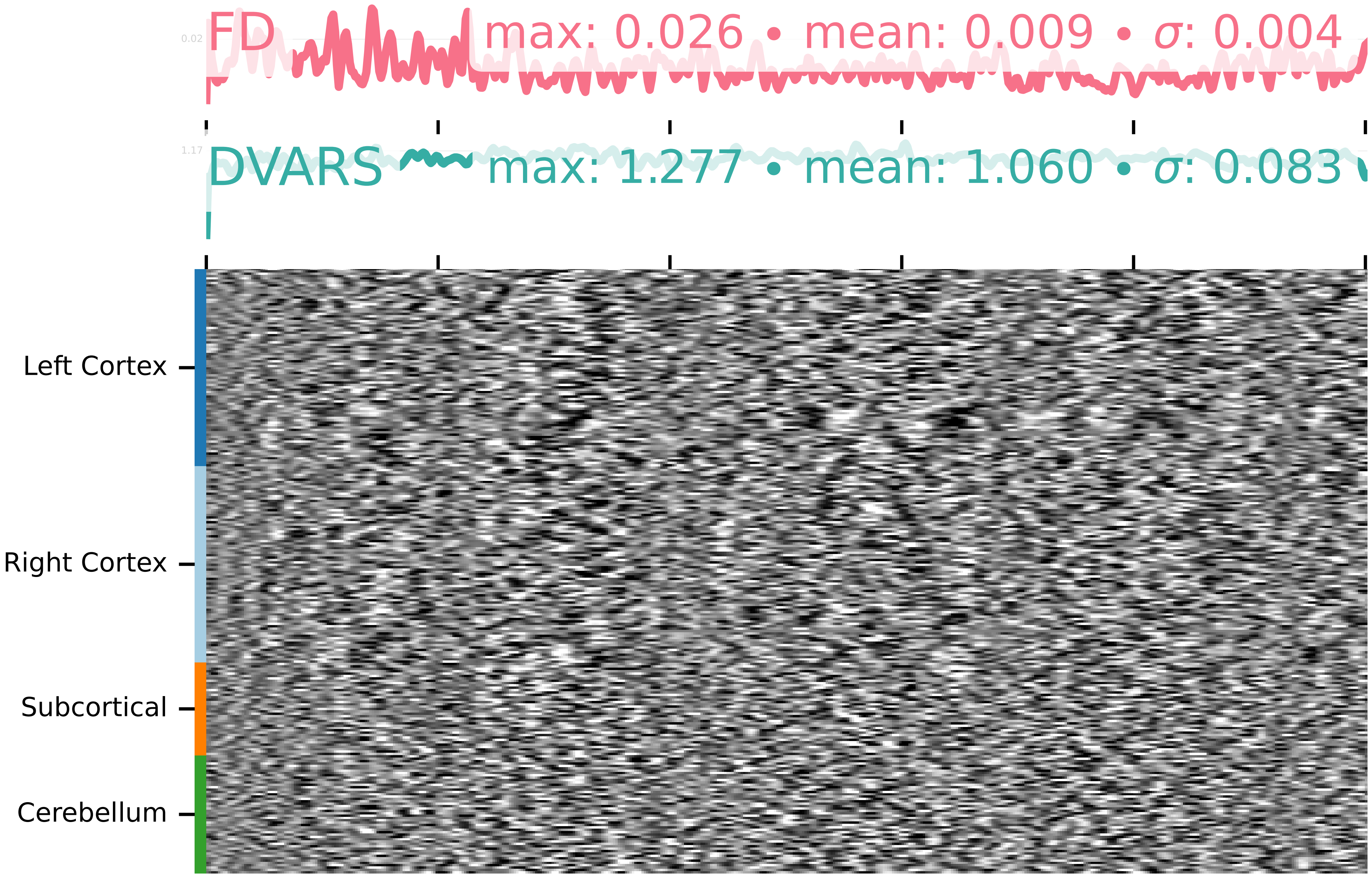

Get figure file: sub-01/figures/sub-01_task-rest_run-02_space-fsLR_desc-postprocessing_bold.svg

Correlation Heatmaps from Four Atlases

This plot shows heatmaps from ROI-to-ROI correlations from four atlases.

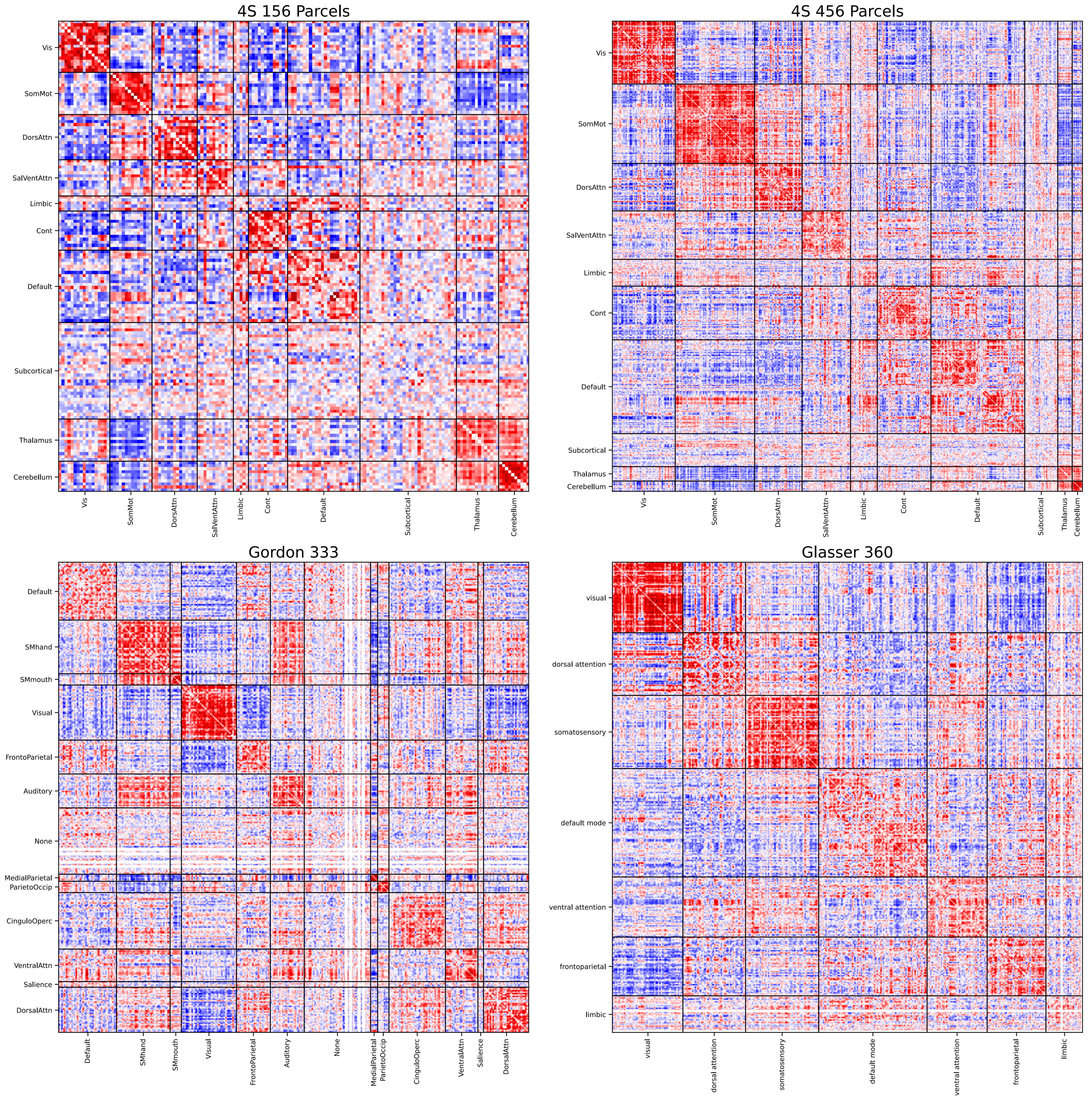

Get figure file: sub-01/figures/sub-01_task-rest_run-02_space-fsLR_desc-connectivityplot_bold.svg

About

- xcp_d version: 0.7.3.dev1+gdf975df
- xcp_d: `/usr/local/miniconda/bin/xcp_d /cbica/home/mehtaka/outputs/fmriprep exec_out participant --combineruns --warp-surfaces-native2std --omp-nthreads 6 --cifti --nthreads 1 --omp-nthreads 1 --mem_gb 10 --smoothing 2 --min_coverage 0.5 --min_time 100 --dummy-scans auto --random-seed 0 --bpf-order 2 --motion-filter-type lp --motion-filter-order 4 --head-radius auto --exact-time 300 480 600 --despike --lower-bpf 0.01 --upper-bpf 0.08 -p 36P -f 0.3 -w /cbica/home/mehtaka/exec_wd -vvv --band-stop-min 6 --input-type fmriprep`
- xcp_d preprocessed: 2024-04-26 17:45:46 -0400

Methods

We kindly ask to report results preprocessed with this tool using the following boilerplate.

HTML [Markdown] [LaTeX]

Post-processing of fmriprep outputs

The eXtensible Connectivity Pipeline- DCAN (XCP-D) (Ciric et al. 2018; Satterthwaite et al. 2013) was used to post-process the outputs of *fMRIPrep* version 23.1.3 (Esteban et al. 2019, 2020, RRID:SCR_016216). XCP-D was built with *Nipype* version 1.8.6 (Gorgolewski et al. 2011, RRID:SCR_002502). Native-space T1w images were transformed to MNI152NLin6Asym space at 1 mm3 resolution. fsLR-space morphometry surfaces were copied from the preprocessing derivatives to the XCP-D derivatives. HCP-style midthickness, inflated, and very-inflated surfaces were generated from the white-matter and pial surface meshes. fsnative-space surfaces were then warped to fsLR space. For each of the two BOLD runs found per subject (across all tasks and sessions), the following post-processing was performed.

Non-steady-state volumes were extracted from the preprocessed confounds and were discarded from both the BOLD data and nuisance regressors. The six translation and rotation head motion traces were low-pass filtered below 6.0 breaths-per-minute using a(n) fourth-order Butterworth filter, based on Gratton et al. (2020). The Volterra expansion of these filtered motion parameters was then calculated. Framewise displacement was calculated from the filtered motion parameters using the formula from Power et al. (2014), with a head radius of 67.98579611765847 mm. Volumes with filtered framewise displacement greater than 0.3 mm were flagged as high-motion outliers for the sake of later censoring (Power et al. 2014). Additional sets of censoring volumes were randomly selected to produce additional correlation matrices limited to 422 volumes. In total, 36 nuisance regressors were selected from the preprocessing confounds, according to the '36P' strategy. These nuisance regressors included six filtered motion parameters, mean global signal, mean white matter signal, mean cerebrospinal fluid signal with their temporal derivatives, and quadratic expansion of six motion parameters, tissue signals and their temporal derivatives (Ciric et al. 2017; Satterthwaite et al. 2013). The BOLD data were converted to NIfTI format, despiked with *AFNI*'s *3dDespike*, and converted back to CIFTI format.

Nuisance regressors were regressed from the BOLD data using a denoising method based on *Nilearn*'s approach. Any volumes censored earlier in the workflow were first cubic spline interpolated in the BOLD data. Outlier volumes at the beginning or end of the time series were replaced with the closest low-motion volume's values, as cubic spline interpolation can produce extreme extrapolations. The timeseries were band-pass filtered using a(n) second-order Butterworth filter, in order to retain signals between 0.01-0.1 Hz. The same filter was applied to the confounds. The resulting time series were then denoised via linear regression, in which the low-motion volumes from the BOLD time series and confounds were used to calculate parameter estimates, and then the interpolated time series were denoised using the low-motion parameter estimates. The interpolated time series were then censored using the temporal mask. The denoised BOLD was then smoothed using *Connectome Workbench* with a Gaussian kernel (FWHM=2.0 mm).

The amplitude of low-frequency fluctuation (ALFF) (Zou et al. 2008) was computed by transforming the mean-centered, standard deviation-normalized, denoised BOLD time series to the frequency domain using the Lomb-Scargle periodogram (Lomb 1976; Scargle 1982; Townsend 2010; Taylor et al. 2018). The power spectrum was computed within the 0.01-0.1 Hz frequency band and the mean square root of the power spectrum was calculated at each voxel to yield voxel-wise ALFF measures. The resulting ALFF values were then multiplied by the standard deviation of the denoised BOLD time series to retain the original scaling. The ALFF maps were smoothed with the Connectome Workbench using a Gaussian kernel (FWHM=2.0 mm).

For each hemisphere, regional homogeneity (ReHo) (Jiang and Zuo 2016) was computed using surface-based *2dReHo* (Zhang et al. 2019). Specifically, for each vertex on the surface, the Kendall's coefficient of concordance (KCC) was computed with nearest-neighbor vertices to yield ReHo. For the subcortical, volumetric data, ReHo was computed with neighborhood voxels using *AFNI*'s *3dReHo* (Taylor and Saad 2013).

Processed functional timeseries were extracted from residual BOLD using Connectome Workbench (Marcus et al. 2011) for the following atlases: the Schaefer Supplemented with Subcortical Structures (4S) atlas (Schaefer et al. 2018; Pauli, Nili, and Tyszka 2018; King et al. 2019; Najdenovska et al. 2018; Glasser et al. 2013) at 10 different resolutions (1056, 156, 256, 356, 456, 556, 656, 756, 856, and 956 parcels), the Glasser atlas (Glasser et al. 2016), the Gordon atlas (Gordon et al. 2016), the Tian subcortical atlas (Tian et al. 2020), and the HCP CIFTI subcortical atlas (Glasser et al. 2013). Corresponding pair-wise functional connectivity between all regions was computed for each atlas, which was operationalized as the Pearson's correlation of each parcel's unsmoothed timeseries with the Connectome Workbench. In cases of partial coverage, uncovered vertices (values of all zeros or NaNs) were either ignored (when the parcel had >50.0% coverage) or were set to zero (when the parcel had <50.0% coverage).

Postprocessing derivatives from multi-run tasks were then concatenated across runs and directions.

Many internal operations of *XCP-D* use *AFNI* (Cox 1996; Cox and Hyde 1997), *Connectome Workbench* (Marcus et al. 2011), *ANTS* (Avants et al. 2009), *TemplateFlow* version 24.2.0 (Ciric et al. 2022), *matplotlib* version 3.8.4 (Hunter 2007), *Nibabel* version 5.2.1 (Brett et al. 2022), *Nilearn* version 0.10.4 (Abraham et al. 2014), *numpy* version 1.26.4 (Harris et al. 2020), *pybids* version 0.16.4 (Yarkoni et al. 2019), and *scipy* version 1.13.0 (Virtanen et al. 2020). For more details, see the *XCP-D* website (https://xcp-d.readthedocs.io).

Copyright Waiver

The above methods description text was automatically generated by *XCP-D* with the express intention that users should copy and paste this text into their manuscripts *unchanged*. It is released under the CC0 license.

References

Abraham, Alexandre, Fabian Pedregosa, Michael Eickenberg, Philippe Gervais, Andreas Mueller, Jean Kossaifi, Alexandre Gramfort, Bertrand Thirion, and Gaël Varoquaux. 2014. "Machine Learning for Neuroimaging with Scikit-Learn." *Frontiers in Neuroinformatics*. Frontiers, 14.

Avants, Brian B, Nick Tustison, Gang Song, and others. 2009. "Advanced Normalization Tools (Ants)." *Insight J* 2 (365): 1–35.

Brett, Matthew, Christopher J. Markiewicz, Michael Hanke, Marc-Alexandre Côté, Ben Cipollini, Paul McCarthy, Dorota Jarecka, et al. 2022. *Nipy/Nibabel:* (version 4.0.0). Zenodo. https://doi.org/10.5281/zenodo.591597.

Ciric, Rastko, Adon F. G. Rosen, Guray Erus, Matthew Cieslak, Azeez Adebimpe, Philip A. Cook, Danielle S. Bassett, Christos Davatzikos, Daniel H. Wolf, and Theodore D. Satterthwaite. 2018. "Mitigating Head Motion Artifact in Functional Connectivity MRI." *Nature Protocols* 13 (12): 2801–26. https://doi.org/10.1038/s41596-018-0065-y.

Ciric, Rastko, William H Thompson, Romy Lorenz, Mathias Goncalves, Eilidh MacNicol, Christopher J Markiewicz, Yaroslav O Halchenko, et al. 2022. "TemplateFlow: FAIR-Sharing of Multi-Scale, Multi-Species Brain Models." *bioRxiv*. Cold Spring Harbor Laboratory, 2021–02. https://doi.org/10.1101/2021.02.10.430678.

Ciric, Rastko, Daniel H. Wolf, Jonathan D. Power, David R. Roalf, Graham Baum, Kosha Ruparel, Russell T. Shinohara, et al. 2017. "Benchmarking of Participant-Level Confound Regression Strategies for the Control of Motion Artifact in Studies of Functional Connectivity." *NeuroImage* 154 (July): 174–87. https://doi.org/10.1016/j.neuroimage.2017.03.020.

Cox, Robert W. 1996. "AFNI: Software for Analysis and Visualization of Functional Magnetic Resonance Neuroimages." *Computers and Biomedical Research* 29 (3). Elsevier: 162–73.

Cox, Robert W, and James S Hyde. 1997. "Software Tools for Analysis and Visualization of fMRI Data." *NMR in Biomedicine: An International Journal Devoted to the Development and Application of Magnetic Resonance in Vivo* 10 (4-5). Wiley Online Library: 171–78.

Esteban, Oscar, Rastko Ciric, Karolina Finc, Ross W Blair, Christopher J Markiewicz, Craig A Moodie, James D Kent, et al. 2020. "Analysis of Task-Based Functional Mri Data Preprocessed with fMRIPrep." *Nature Protocols* 15 (7). Nature Publishing Group: 2186–2202. https://doi.org/10.1038/s41596-020-0327-3.

Esteban, Oscar, Christopher J Markiewicz, Ross W Blair, Craig A Moodie, A Ilkay Isik, Asier Erramuzpe, James D Kent, et al. 2019. "FMRIPrep: A Robust Preprocessing Pipeline for Functional Mri." *Nature Methods* 16 (1). Nature Publishing Group: 111–16. https://doi.org/10.1038/s41592-018-0235-4.

Glasser, Matthew F., Timothy S. Coalson, Emma C. Robinson, Carl D. Hacker, John Harwell, Essa Yacoub, Kamil Ugurbil, et al. 2016. "A Multi-Modal Parcellation of Human Cerebral Cortex." *Nature* 536 (7615): 171–78. https://doi.org/10.1038/nature18933.

Glasser, Matthew F., Stamatios N. Sotiropoulos, J. Anthony Wilson, Timothy S. Coalson, Bruce Fischl, Jesper L. Andersson, Junqian Xu, et al. 2013. "The Minimal Preprocessing Pipelines for the Human Connectome Project." *NeuroImage* 80 (October): 105–24. https://doi.org/10.1016/j.neuroimage.2013.04.127.

Gordon, Evan M., Timothy O. Laumann, Babatunde Adeyemo, Jeremy F. Huckins, William M. Kelley, and Steven E. Petersen. 2016. "Generation and Evaluation of a Cortical Area Parcellation from Resting-State Correlations." *Cerebral Cortex* 26 (1): 288–303. https://doi.org/10.1093/cercor/bhu239.

Gorgolewski, Krzysztof, Christopher D. Burns, Cindee Madison, Dav Clark, Yaroslav O. Halchenko, Michael L. Waskom, and Satrajit S. Ghosh. 2011. "Nipype: A Flexible, Lightweight and Extensible Neuroimaging Data Processing Framework in Python." *Frontiers in Neuroinformatics* 5. https://doi.org/10.3389/fninf.2011.00013.

Gratton, Caterina, Ally Dworetsky, Rebecca S Coalson, Babatunde Adeyemo, Timothy O Laumann, Gagan S Wig, Tania S Kong, et al. 2020. "Removal of High Frequency Contamination from Motion Estimates in Single-Band fMRI Saves Data Without Biasing Functional Connectivity." *Neuroimage* 217. Elsevier: 116866. https://doi.org/10.1016/j.neuroimage.2020.116866.

Harris, Charles R., Jarrod K. Millman, Stéfan J. van der Walt, Ralf Gommers, Pauli Virtanen, David Cournapeau, Eric Wieser, et al. 2020. "Array Programming with NumPy." *Nature* 585 (7825): 357–62. https://doi.org/10.1038/s41586-020-2649-2.

Hunter, John D. 2007. "Matplotlib: A 2D Graphics Environment." *Computing in Science & Engineering* 9 (03). IEEE Computer Society: 90–95.

Jiang, Lili, and Xi-Nian Zuo. 2016. "Regional Homogeneity: A Multimodal, Multiscale Neuroimaging Marker of the Human Connectome." *The Neuroscientist* 22 (5). Sage Publications Sage CA: Los Angeles, CA: 486–505.

King, Maedbh, Carlos R Hernandez-Castillo, Russell A Poldrack, Richard B Ivry, and Jörn Diedrichsen. 2019. "Functional Boundaries in the Human Cerebellum Revealed by a Multi-Domain Task Battery." *Nature Neuroscience* 22 (8). Nature Publishing Group US New York: 1371–8. https://doi.org/10.1038/s41593-019-0436-x.

Lomb, Nicholas R. 1976. "Least-Squares Frequency Analysis of Unequally Spaced Data." *Astrophysics and Space Science* 39. Springer: 447–62.

Marcus, Daniel S, John Harwell, Timothy Olsen, Michael Hodge, Matthew F Glasser, Fred Prior, Mark Jenkinson, Timothy Laumann, Sandra W Curtiss, and David C Van Essen. 2011. "Informatics and Data Mining Tools and Strategies for the Human Connectome Project." *Frontiers in Neuroinformatics* 5. Frontiers Research Foundation: 4.

Najdenovska, Elena, Yasser Alemán-Gómez, Giovanni Battistella, Maxime Descoteaux, Patric Hagmann, Sebastien Jacquemont, Philippe Maeder, Jean-Philippe Thiran, Eleonora Fornari, and Meritxell Bach Cuadra. 2018. "In-Vivo Probabilistic Atlas of Human Thalamic Nuclei Based on Diffusion-Weighted Magnetic Resonance Imaging." *Scientific Data* 5 (1). Nature Publishing Group: 1–11. https://doi.org/10.1038/sdata.2018.270.

Pauli, Wolfgang M, Amanda N Nili, and J Michael Tyszka. 2018. "A High-Resolution Probabilistic in Vivo Atlas of Human Subcortical Brain Nuclei." *Scientific Data* 5 (1). Nature Publishing Group: 1–13. https://doi.org/10.1038/sdata.2018.63.

Power, Jonathan D., Anish Mitra, Timothy O. Laumann, Abraham Z. Snyder, Bradley L. Schlaggar, and Steven E. Petersen. 2014. "Methods to Detect, Characterize, and Remove Motion Artifact in Resting State fMRI." *NeuroImage* 84 (January): 320–41. https://doi.org/10.1016/j.neuroimage.2013.08.048.

Satterthwaite, Theodore D., Mark A. Elliott, Raphael T. Gerraty, Kosha Ruparel, James Loughead, Monica E. Calkins, Simon B. Eickhoff, et al. 2013. "An Improved Framework for Confound Regression and Filtering for Control of Motion Artifact in the Preprocessing of Resting-State Functional Connectivity Data." *NeuroImage* 64 (January): 240–56. https://doi.org/10.1016/j.neuroimage.2012.08.052.

Scargle, Jeffrey D. 1982. "Studies in Astronomical Time Series Analysis. II-Statistical Aspects of Spectral Analysis of Unevenly Spaced Data." *Astrophysical Journal, Part 1, Vol. 263, Dec. 15, 1982, P. 835-853.* 263: 835–53.

Schaefer, Alexander, Ru Kong, Evan M. Gordon, Timothy O. Laumann, Xi-Nian Zuo, Avram J. Holmes, Simon B. Eickhoff, and B. T. Thomas Yeo. 2018. "Local-Global Parcellation of the Human Cerebral Cortex from Intrinsic Functional Connectivity MRI." *Cerebral Cortex (New York, N.Y.: 1991)* 28 (9): 3095–3114. https://doi.org/10.1093/cercor/bhx179.

Taylor, Paul A, Gang Chen, Daniel R Glen, Richard C Reynolds, and Robert W Cox. 2018. "Lomb-Scargle Your Way to Rsfc Parameter Estimation in Afni-Fatcat." *International Society for Magnetic Resonance in Medicine*. International Society for Magnetic Resonance in Medicine.

Taylor, Paul A, and Ziad S Saad. 2013. "FATCAT:(An Efficient) Functional and Tractographic Connectivity Analysis Toolbox." *Brain Connectivity* 3 (5). Mary Ann Liebert, Inc. 140 Huguenot Street, 3rd Floor New Rochelle, NY 10801 USA: 523–35.

Tian, Ye, Daniel S Margulies, Michael Breakspear, and Andrew Zalesky. 2020. "Topographic Organization of the Human Subcortex Unveiled with Functional Connectivity Gradients." *Nature Neuroscience* 23 (11). Nature Publishing Group: 1421–32. https://doi.org/10.1038/s41593-020-00711-6.

Townsend, RHD. 2010. "Fast Calculation of the Lomb–Scargle Periodogram Using Graphics Processing Units." *The Astrophysical Journal Supplement Series* 191 (2). IOP Publishing: 247.

Virtanen, Pauli, Ralf Gommers, Travis E. Oliphant, Matt Haberland, Tyler Reddy, David Cournapeau, Evgeni Burovski, et al. 2020. "SciPy 1.0: Fundamental Algorithms for Scientific Computing in Python." *Nature Methods* 17 (3): 261–72. https://doi.org/10.1038/s41592-019-0686-2.

Yarkoni, Tal, Christopher J Markiewicz, Alejandro de la Vega, Krzysztof J Gorgolewski, Taylor Salo, Yaroslav O Halchenko, Quinten McNamara, et al. 2019. "PyBIDS: Python Tools for Bids Datasets." *Journal of Open Source Software* 4 (40). NIH Public Access.

Zhang, Bo, Fei Wang, Hao-Ming Dong, Xiao-Wei Jiang, Sheng-Nan Wei, Miao Chang, Zhi-Yang Yin, et al. 2019. "Surface-Based Regional Homogeneity in Bipolar Disorder: A Resting-State fMRI Study." *Psychiatry Research* 278 (August): 199–204. https://doi.org/10.1016/j.psychres.2019.05.045.

Zou, Qi-Hong, Chao-Zhe Zhu, Yihong Yang, Xi-Nian Zuo, Xiang-Yu Long, Qing-Jiu Cao, Yu-Feng Wang, and Yu-Feng Zang. 2008. "An Improved Approach to Detection of Amplitude of Low-Frequency Fluctuation (ALFF) for Resting-State fMRI: Fractional ALFF." *Journal of Neuroscience Methods* 172 (1): 137–41. https://doi.org/10.1016/j.jneumeth.2008.04.012.

Errors

No errors to report!

```
xcp_d
├── sub-20712_executive_summary.html
├── sub-20712.html
├── sub-20712_ses-12367_executive_summary.html
├── dataset_description.json
├── desc-linc_qc.json
├── logs
    ├── CITATION.bib
    ├── CITATION.html
    ├── CITATION.md
    └── CITATION.tex
├── atlases
    ├── dataset_description.json
    ├── atlas-4S1056Parcels
        ├── atlas-4S1056Parcels_dseg.json
        ├── atlas-4S1056Parcels_dseg.tsv
        └── atlas-4S1056Parcels_space-MNI152NLin6Asym_res-2_dseg.nii.gz
    │
    ├── atlas-4S156Parcels
        ├── atlas-4S156Parcels_dseg.json
        ├── atlas-4S156Parcels_dseg.tsv
        └── atlas-4S156Parcels_space-MNI152NLin6Asym_res-2_dseg.nii.gz
    │
    ├── atlas-4S256Parcels
        ├── atlas-4S256Parcels_dseg.json
        ├── atlas-4S256Parcels_dseg.tsv
        └── atlas-4S256Parcels_space-MNI152NLin6Asym_res-2_dseg.nii.gz
    │
    ├── atlas-4S356Parcels
        ├── atlas-4S356Parcels_dseg.json
        ├── atlas-4S356Parcels_dseg.tsv
        └── atlas-4S356Parcels_space-MNI152NLin6Asym_res-2_dseg.nii.gz
    │
    ├── atlas-4S456Parcels
        ├── atlas-4S456Parcels_dseg.json
        ├── atlas-4S456Parcels_dseg.tsv
        └── atlas-4S456Parcels_space-MNI152NLin6Asym_res-2_dseg.nii.gz
    │
    ├── atlas-4S556Parcels
        ├── atlas-4S556Parcels_dseg.json
        ├── atlas-4S556Parcels_dseg.tsv
        └── atlas-4S556Parcels_space-MNI152NLin6Asym_res-2_dseg.nii.gz
    │
    ├── atlas-4S656Parcels
        ├── atlas-4S656Parcels_dseg.json
        ├── atlas-4S656Parcels_dseg.tsv
        └── atlas-4S656Parcels_space-MNI152NLin6Asym_res-2_dseg.nii.gz
    │
    ├── atlas-4S756Parcels
        ├── atlas-4S756Parcels_dseg.json
        ├── atlas-4S756Parcels_dseg.tsv
        └── atlas-4S756Parcels_space-MNI152NLin6Asym_res-2_dseg.nii.gz
    │
    ├── atlas-4S856Parcels
        ├── atlas-4S856Parcels_dseg.json
        ├── atlas-4S856Parcels_dseg.tsv
        └── atlas-4S856Parcels_space-MNI152NLin6Asym_res-2_dseg.nii.gz
    │
    ├── atlas-4S956Parcels
        ├── atlas-4S956Parcels_dseg.json
        ├── atlas-4S956Parcels_dseg.tsv
        └── atlas-4S956Parcels_space-MNI152NLin6Asym_res-2_dseg.nii.gz
    │
    ├── atlas-Glasser
```

```
        ├── atlas-Glasser_dseg.json
        ├── atlas-Glasser_dseg.tsv
    │   └── atlas-Glasser_space-MNI152NLin6Asym_res-2_dseg.nii.gz
    ├── atlas-Gordon
        ├── atlas-Gordon_dseg.json
        ├── atlas-Gordon_dseg.tsv
        └── atlas-Gordon_space-MNI152NLin6Asym_res-2_dseg.nii.gz
    ├── atlas-HCP
        ├── atlas-HCP_dseg.json
        ├── atlas-HCP_dseg.tsv
        └── atlas-HCP_space-MNI152NLin6Asym_res-2_dseg.nii.gz
    ├── atlas-Tian
        ├── atlas-Tian_dseg.json
        ├── atlas-Tian_dseg.tsv
        └── atlas-Tian_space-MNI152NLin6Asym_res-2_dseg.nii.gz
├── sub-20712
    ├── ses-12367
      ├── anat
            ├── sub-20712_ses-12367_rec-defaced_space-
MNI152NLin6Asym_desc-preproc_T1w.nii.gz
            ├── sub-20712_ses-12367_rec-defaced_space-
MNI152NLin6Asym_dseg.nii.gz
      ├── func
            ├── sub-20712_ses-12367_task-restbold_run-1_desc-dcan_qc.hdf5
            ├── sub-20712_ses-12367_task-restbold_run-1_desc-
preproc_design.json
            ├── sub-20712_ses-12367_task-restbold_run-1_desc-
preproc_design.tsv
            ├── sub-20712_ses-12367_task-restbold_run-1_motion.json
            ├── sub-20712_ses-12367_task-restbold_run-1_motion.tsv
            ├── sub-20712_ses-12367_task-restbold_run-1_outliers.json
            ├── sub-20712_ses-12367_task-restbold_run-1_outliers.tsv
            ├── sub-20712_ses-12367_task-restbold_run-1_space-
MNI152NLin6Asym_desc-linc_qc.tsv
            ├── sub-20712_ses-12367_task-restbold_run-1_space-
MNI152NLin6Asym_res-2_desc-denoised_bold.json
            ├── sub-20712_ses-12367_task-restbold_run-1_space-
MNI152NLin6Asym_res-2_desc-denoised_bold.nii.gz
            ├── sub-20712_ses-12367_task-restbold_run-1_space-
MNI152NLin6Asym_res-2_desc-denoisedSmoothed_bold.json
            ├── sub-20712_ses-12367_task-restbold_run-1_space-
MNI152NLin6Asym_res-2_desc-denoisedSmoothed_bold.nii.gz
            ├── sub-20712_ses-12367_task-restbold_run-1_space-
MNI152NLin6Asym_res-2_desc-interpolated_bold.json
            ├── sub-20712_ses-12367_task-restbold_run-1_space-
MNI152NLin6Asym_res-2_desc-interpolated_bold.nii.gz
            ├── sub-20712_ses-12367_task-restbold_run-1_space-
MNI152NLin6Asym_res-2_stat-alff_boldmap.json
            ├── sub-20712_ses-12367_task-restbold_run-1_space-
MNI152NLin6Asym_res-2_stat-alff_boldmap.nii.gz
            ├── sub-20712_ses-12367_task-restbold_run-1_space-
MNI152NLin6Asym_res-2_stat-alff_desc-smooth_boldmap.json
            ├── sub-20712_ses-12367_task-restbold_run-1_space-
MNI152NLin6Asym_res-2_stat-alff_desc-smooth_boldmap.nii.gz
```

```
            ├── sub-20712_ses-12367_task-restbold_run-1_space-
MNI152NLin6Asym_res-2_stat-reho_boldmap.json
            ├── sub-20712_ses-12367_task-restbold_run-1_space-
MNI152NLin6Asym_res-2_stat-reho_boldmap.nii.gz
            ├── sub-20712_ses-12367_task-restbold_run-1_space-
MNI152NLin6Asym_seg-4S1056Parcels_stat-alff_bold.json
            ├── sub-20712_ses-12367_task-restbold_run-1_space-
MNI152NLin6Asym_seg-4S1056Parcels_stat-alff_bold.tsv
            ├── sub-20712_ses-12367_task-restbold_run-1_space-
MNI152NLin6Asym_seg-4S1056Parcels_stat-coverage_bold.json
            ├── sub-20712_ses-12367_task-restbold_run-1_space-
MNI152NLin6Asym_seg-4S1056Parcels_stat-coverage_bold.tsv
            ├── sub-20712_ses-12367_task-restbold_run-1_space-
MNI152NLin6Asym_seg-4S1056Parcels_stat-mean_timeseries.json
            ├── sub-20712_ses-12367_task-restbold_run-1_space-
MNI152NLin6Asym_seg-4S1056Parcels_stat-mean_timeseries.tsv
            ├── sub-20712_ses-12367_task-restbold_run-1_space-
MNI152NLin6Asym_seg-4S1056Parcels_stat-pearsoncorrelation_relmat.json
            ├── sub-20712_ses-12367_task-restbold_run-1_space-
MNI152NLin6Asym_seg-4S1056Parcels_stat-pearsoncorrelation_relmat.tsv
            ├── sub-20712_ses-12367_task-restbold_run-1_space-
MNI152NLin6Asym_seg-4S1056Parcels_stat-reho_bold.json
            ├── sub-20712_ses-12367_task-restbold_run-1_space-
MNI152NLin6Asym_seg-4S1056Parcels_stat-reho_bold.tsv
            ├── sub-20712_ses-12367_task-restbold_run-1_space-
MNI152NLin6Asym_seg-4S156Parcels_stat-alff_bold.json
            ├── sub-20712_ses-12367_task-restbold_run-1_space-
MNI152NLin6Asym_seg-4S156Parcels_stat-alff_bold.tsv
            ├── sub-20712_ses-12367_task-restbold_run-1_space-
MNI152NLin6Asym_seg-4S156Parcels_stat-coverage_bold.json
            ├── sub-20712_ses-12367_task-restbold_run-1_space-
MNI152NLin6Asym_seg-4S156Parcels_stat-coverage_bold.tsv
            ├── sub-20712_ses-12367_task-restbold_run-1_space-
MNI152NLin6Asym_seg-4S156Parcels_stat-mean_timeseries.json
            ├── sub-20712_ses-12367_task-restbold_run-1_space-
MNI152NLin6Asym_seg-4S156Parcels_stat-mean_timeseries.tsv
            ├── sub-20712_ses-12367_task-restbold_run-1_space-
MNI152NLin6Asym_seg-4S156Parcels_stat-pearsoncorrelation_relmat.json
            ├── sub-20712_ses-12367_task-restbold_run-1_space-
MNI152NLin6Asym_seg-4S156Parcels_stat-pearsoncorrelation_relmat.tsv
            ├── sub-20712_ses-12367_task-restbold_run-1_space-
MNI152NLin6Asym_seg-4S156Parcels_stat-reho_bold.json
            ├── sub-20712_ses-12367_task-restbold_run-1_space-
MNI152NLin6Asym_seg-4S156Parcels_stat-reho_bold.tsv
            ├── sub-20712_ses-12367_task-restbold_run-1_space-
MNI152NLin6Asym_seg-4S256Parcels_stat-alff_bold.json
            ├── sub-20712_ses-12367_task-restbold_run-1_space-
MNI152NLin6Asym_seg-4S256Parcels_stat-alff_bold.tsv
            ├── sub-20712_ses-12367_task-restbold_run-1_space-
MNI152NLin6Asym_seg-4S256Parcels_stat-coverage_bold.json
            ├── sub-20712_ses-12367_task-restbold_run-1_space-
MNI152NLin6Asym_seg-4S256Parcels_stat-coverage_bold.tsv
            ├── sub-20712_ses-12367_task-restbold_run-1_space-
MNI152NLin6Asym_seg-4S256Parcels_stat-mean_timeseries.json
```

```
        ├── sub-20712_ses-12367_task-restbold_run-1_space-
MNI152NLin6Asym_seg-4S256Parcels_stat-mean_timeseries.tsv
        ├── sub-20712_ses-12367_task-restbold_run-1_space-
MNI152NLin6Asym_seg-4S256Parcels_stat-pearsoncorrelation_relmat.json
        ├── sub-20712_ses-12367_task-restbold_run-1_space-
MNI152NLin6Asym_seg-4S256Parcels_stat-pearsoncorrelation_relmat.tsv
        ├── sub-20712_ses-12367_task-restbold_run-1_space-
MNI152NLin6Asym_seg-4S256Parcels_stat-reho_bold.json
        ├── sub-20712_ses-12367_task-restbold_run-1_space-
MNI152NLin6Asym_seg-4S256Parcels_stat-reho_bold.tsv
        ├── sub-20712_ses-12367_task-restbold_run-1_space-
MNI152NLin6Asym_seg-4S356Parcels_stat-alff_bold.json
        ├── sub-20712_ses-12367_task-restbold_run-1_space-
MNI152NLin6Asym_seg-4S356Parcels_stat-alff_bold.tsv
        ├── sub-20712_ses-12367_task-restbold_run-1_space-
MNI152NLin6Asym_seg-4S356Parcels_stat-coverage_bold.json
        ├── sub-20712_ses-12367_task-restbold_run-1_space-
MNI152NLin6Asym_seg-4S356Parcels_stat-coverage_bold.tsv
        ├── sub-20712_ses-12367_task-restbold_run-1_space-
MNI152NLin6Asym_seg-4S356Parcels_stat-mean_timeseries.json
        ├── sub-20712_ses-12367_task-restbold_run-1_space-
MNI152NLin6Asym_seg-4S356Parcels_stat-mean_timeseries.tsv
        ├── sub-20712_ses-12367_task-restbold_run-1_space-
MNI152NLin6Asym_seg-4S356Parcels_stat-pearsoncorrelation_relmat.json
        ├── sub-20712_ses-12367_task-restbold_run-1_space-
MNI152NLin6Asym_seg-4S356Parcels_stat-pearsoncorrelation_relmat.tsv
        ├── sub-20712_ses-12367_task-restbold_run-1_space-
MNI152NLin6Asym_seg-4S356Parcels_stat-reho_bold.json
        ├── sub-20712_ses-12367_task-restbold_run-1_space-
MNI152NLin6Asym_seg-4S356Parcels_stat-reho_bold.tsv
        ├── sub-20712_ses-12367_task-restbold_run-1_space-
MNI152NLin6Asym_seg-4S456Parcels_stat-alff_bold.json
        ├── sub-20712_ses-12367_task-restbold_run-1_space-
MNI152NLin6Asym_seg-4S456Parcels_stat-alff_bold.tsv
        ├── sub-20712_ses-12367_task-restbold_run-1_space-
MNI152NLin6Asym_seg-4S456Parcels_stat-coverage_bold.json
        ├── sub-20712_ses-12367_task-restbold_run-1_space-
MNI152NLin6Asym_seg-4S456Parcels_stat-coverage_bold.tsv
        ├── sub-20712_ses-12367_task-restbold_run-1_space-
MNI152NLin6Asym_seg-4S456Parcels_stat-mean_timeseries.json
        ├── sub-20712_ses-12367_task-restbold_run-1_space-
MNI152NLin6Asym_seg-4S456Parcels_stat-mean_timeseries.tsv
        ├── sub-20712_ses-12367_task-restbold_run-1_space-
MNI152NLin6Asym_seg-4S456Parcels_stat-pearsoncorrelation_relmat.json
        ├── sub-20712_ses-12367_task-restbold_run-1_space-
MNI152NLin6Asym_seg-4S456Parcels_stat-pearsoncorrelation_relmat.tsv
        ├── sub-20712_ses-12367_task-restbold_run-1_space-
MNI152NLin6Asym_seg-4S456Parcels_stat-reho_bold.json
        ├── sub-20712_ses-12367_task-restbold_run-1_space-
MNI152NLin6Asym_seg-4S456Parcels_stat-reho_bold.tsv
        ├── sub-20712_ses-12367_task-restbold_run-1_space-
MNI152NLin6Asym_seg-4S556Parcels_stat-alff_bold.json
        ├── sub-20712_ses-12367_task-restbold_run-1_space-
MNI152NLin6Asym_seg-4S556Parcels_stat-alff_bold.tsv
```

```
            ├── sub-20712_ses-12367_task-restbold_run-1_space-
MNI152NLin6Asym_seg-4S556Parcels_stat-coverage_bold.json
            ├── sub-20712_ses-12367_task-restbold_run-1_space-
MNI152NLin6Asym_seg-4S556Parcels_stat-coverage_bold.tsv
            ├── sub-20712_ses-12367_task-restbold_run-1_space-
MNI152NLin6Asym_seg-4S556Parcels_stat-mean_timeseries.json
            ├── sub-20712_ses-12367_task-restbold_run-1_space-
MNI152NLin6Asym_seg-4S556Parcels_stat-mean_timeseries.tsv
            ├── sub-20712_ses-12367_task-restbold_run-1_space-
MNI152NLin6Asym_seg-4S556Parcels_stat-pearsoncorrelation_relmat.json
            ├── sub-20712_ses-12367_task-restbold_run-1_space-
MNI152NLin6Asym_seg-4S556Parcels_stat-pearsoncorrelation_relmat.tsv
            ├── sub-20712_ses-12367_task-restbold_run-1_space-
MNI152NLin6Asym_seg-4S556Parcels_stat-reho_bold.json
            ├── sub-20712_ses-12367_task-restbold_run-1_space-
MNI152NLin6Asym_seg-4S556Parcels_stat-reho_bold.tsv
            ├── sub-20712_ses-12367_task-restbold_run-1_space-
MNI152NLin6Asym_seg-4S656Parcels_stat-alff_bold.json
            ├── sub-20712_ses-12367_task-restbold_run-1_space-
MNI152NLin6Asym_seg-4S656Parcels_stat-alff_bold.tsv
            ├── sub-20712_ses-12367_task-restbold_run-1_space-
MNI152NLin6Asym_seg-4S656Parcels_stat-coverage_bold.json
            ├── sub-20712_ses-12367_task-restbold_run-1_space-
MNI152NLin6Asym_seg-4S656Parcels_stat-coverage_bold.tsv
            ├── sub-20712_ses-12367_task-restbold_run-1_space-
MNI152NLin6Asym_seg-4S656Parcels_stat-mean_timeseries.json
            ├── sub-20712_ses-12367_task-restbold_run-1_space-
MNI152NLin6Asym_seg-4S656Parcels_stat-mean_timeseries.tsv
            ├── sub-20712_ses-12367_task-restbold_run-1_space-
MNI152NLin6Asym_seg-4S656Parcels_stat-pearsoncorrelation_relmat.json
            ├── sub-20712_ses-12367_task-restbold_run-1_space-
MNI152NLin6Asym_seg-4S656Parcels_stat-pearsoncorrelation_relmat.tsv
            ├── sub-20712_ses-12367_task-restbold_run-1_space-
MNI152NLin6Asym_seg-4S656Parcels_stat-reho_bold.json
            ├── sub-20712_ses-12367_task-restbold_run-1_space-
MNI152NLin6Asym_seg-4S656Parcels_stat-reho_bold.tsv
            ├── sub-20712_ses-12367_task-restbold_run-1_space-
MNI152NLin6Asym_seg-4S756Parcels_stat-alff_bold.json
            ├── sub-20712_ses-12367_task-restbold_run-1_space-
MNI152NLin6Asym_seg-4S756Parcels_stat-alff_bold.tsv
            ├── sub-20712_ses-12367_task-restbold_run-1_space-
MNI152NLin6Asym_seg-4S756Parcels_stat-coverage_bold.json
            ├── sub-20712_ses-12367_task-restbold_run-1_space-
MNI152NLin6Asym_seg-4S756Parcels_stat-coverage_bold.tsv
            ├── sub-20712_ses-12367_task-restbold_run-1_space-
MNI152NLin6Asym_seg-4S756Parcels_stat-mean_timeseries.json
            ├── sub-20712_ses-12367_task-restbold_run-1_space-
MNI152NLin6Asym_seg-4S756Parcels_stat-mean_timeseries.tsv
            ├── sub-20712_ses-12367_task-restbold_run-1_space-
MNI152NLin6Asym_seg-4S756Parcels_stat-pearsoncorrelation_relmat.json
            ├── sub-20712_ses-12367_task-restbold_run-1_space-
MNI152NLin6Asym_seg-4S756Parcels_stat-pearsoncorrelation_relmat.tsv
            ├── sub-20712_ses-12367_task-restbold_run-1_space-
MNI152NLin6Asym_seg-4S756Parcels_stat-reho_bold.json
```

```
            ├── sub-20712_ses-12367_task-restbold_run-1_space-
MNI152NLin6Asym_seg-4S756Parcels_stat-reho_bold.tsv
            ├── sub-20712_ses-12367_task-restbold_run-1_space-
MNI152NLin6Asym_seg-4S856Parcels_stat-alff_bold.json
            ├── sub-20712_ses-12367_task-restbold_run-1_space-
MNI152NLin6Asym_seg-4S856Parcels_stat-alff_bold.tsv
            ├── sub-20712_ses-12367_task-restbold_run-1_space-
MNI152NLin6Asym_seg-4S856Parcels_stat-coverage_bold.json
            ├── sub-20712_ses-12367_task-restbold_run-1_space-
MNI152NLin6Asym_seg-4S856Parcels_stat-coverage_bold.tsv
            ├── sub-20712_ses-12367_task-restbold_run-1_space-
MNI152NLin6Asym_seg-4S856Parcels_stat-mean_timeseries.json
            ├── sub-20712_ses-12367_task-restbold_run-1_space-
MNI152NLin6Asym_seg-4S856Parcels_stat-mean_timeseries.tsv
            ├── sub-20712_ses-12367_task-restbold_run-1_space-
MNI152NLin6Asym_seg-4S856Parcels_stat-pearsoncorrelation_relmat.json
            ├── sub-20712_ses-12367_task-restbold_run-1_space-
MNI152NLin6Asym_seg-4S856Parcels_stat-pearsoncorrelation_relmat.tsv
            ├── sub-20712_ses-12367_task-restbold_run-1_space-
MNI152NLin6Asym_seg-4S856Parcels_stat-reho_bold.json
            ├── sub-20712_ses-12367_task-restbold_run-1_space-
MNI152NLin6Asym_seg-4S856Parcels_stat-reho_bold.tsv
            ├── sub-20712_ses-12367_task-restbold_run-1_space-
MNI152NLin6Asym_seg-4S956Parcels_stat-alff_bold.json
            ├── sub-20712_ses-12367_task-restbold_run-1_space-
MNI152NLin6Asym_seg-4S956Parcels_stat-alff_bold.tsv
            ├── sub-20712_ses-12367_task-restbold_run-1_space-
MNI152NLin6Asym_seg-4S956Parcels_stat-coverage_bold.json
            ├── sub-20712_ses-12367_task-restbold_run-1_space-
MNI152NLin6Asym_seg-4S956Parcels_stat-coverage_bold.tsv
            ├── sub-20712_ses-12367_task-restbold_run-1_space-
MNI152NLin6Asym_seg-4S956Parcels_stat-mean_timeseries.json
            ├── sub-20712_ses-12367_task-restbold_run-1_space-
MNI152NLin6Asym_seg-4S956Parcels_stat-mean_timeseries.tsv
            ├── sub-20712_ses-12367_task-restbold_run-1_space-
MNI152NLin6Asym_seg-4S956Parcels_stat-pearsoncorrelation_relmat.json
            ├── sub-20712_ses-12367_task-restbold_run-1_space-
MNI152NLin6Asym_seg-4S956Parcels_stat-pearsoncorrelation_relmat.tsv
            ├── sub-20712_ses-12367_task-restbold_run-1_space-
MNI152NLin6Asym_seg-4S956Parcels_stat-reho_bold.json
            ├── sub-20712_ses-12367_task-restbold_run-1_space-
MNI152NLin6Asym_seg-4S956Parcels_stat-reho_bold.tsv
            ├── sub-20712_ses-12367_task-restbold_run-1_space-
MNI152NLin6Asym_seg-Glasser_stat-alff_bold.json
            ├── sub-20712_ses-12367_task-restbold_run-1_space-
MNI152NLin6Asym_seg-Glasser_stat-alff_bold.tsv
            ├── sub-20712_ses-12367_task-restbold_run-1_space-
MNI152NLin6Asym_seg-Glasser_stat-coverage_bold.json
            ├── sub-20712_ses-12367_task-restbold_run-1_space-
MNI152NLin6Asym_seg-Glasser_stat-coverage_bold.tsv
            ├── sub-20712_ses-12367_task-restbold_run-1_space-
MNI152NLin6Asym_seg-Glasser_stat-mean_timeseries.json
            ├── sub-20712_ses-12367_task-restbold_run-1_space-
MNI152NLin6Asym_seg-Glasser_stat-mean_timeseries.tsv
```

```
            ├── sub-20712_ses-12367_task-restbold_run-1_space-
MNI152NLin6Asym_seg-Glasser_stat-pearsoncorrelation_relmat.json
            ├── sub-20712_ses-12367_task-restbold_run-1_space-
MNI152NLin6Asym_seg-Glasser_stat-pearsoncorrelation_relmat.tsv
            ├── sub-20712_ses-12367_task-restbold_run-1_space-
MNI152NLin6Asym_seg-Glasser_stat-reho_bold.json
            ├── sub-20712_ses-12367_task-restbold_run-1_space-
MNI152NLin6Asym_seg-Glasser_stat-reho_bold.tsv
            ├── sub-20712_ses-12367_task-restbold_run-1_space-
MNI152NLin6Asym_seg-Gordon_stat-alff_bold.json
            ├── sub-20712_ses-12367_task-restbold_run-1_space-
MNI152NLin6Asym_seg-Gordon_stat-alff_bold.tsv
            ├── sub-20712_ses-12367_task-restbold_run-1_space-
MNI152NLin6Asym_seg-Gordon_stat-coverage_bold.json
            ├── sub-20712_ses-12367_task-restbold_run-1_space-
MNI152NLin6Asym_seg-Gordon_stat-coverage_bold.tsv
            ├── sub-20712_ses-12367_task-restbold_run-1_space-
MNI152NLin6Asym_seg-Gordon_stat-mean_timeseries.json
            ├── sub-20712_ses-12367_task-restbold_run-1_space-
MNI152NLin6Asym_seg-Gordon_stat-mean_timeseries.tsv
            ├── sub-20712_ses-12367_task-restbold_run-1_space-
MNI152NLin6Asym_seg-Gordon_stat-pearsoncorrelation_relmat.json
            ├── sub-20712_ses-12367_task-restbold_run-1_space-
MNI152NLin6Asym_seg-Gordon_stat-pearsoncorrelation_relmat.tsv
            ├── sub-20712_ses-12367_task-restbold_run-1_space-
MNI152NLin6Asym_seg-Gordon_stat-reho_bold.json
            ├── sub-20712_ses-12367_task-restbold_run-1_space-
MNI152NLin6Asym_seg-Gordon_stat-reho_bold.tsv
            ├── sub-20712_ses-12367_task-restbold_run-1_space-
MNI152NLin6Asym_seg-HCP_stat-alff_bold.json
            ├── sub-20712_ses-12367_task-restbold_run-1_space-
MNI152NLin6Asym_seg-HCP_stat-alff_bold.tsv
            ├── sub-20712_ses-12367_task-restbold_run-1_space-
MNI152NLin6Asym_seg-HCP_stat-coverage_bold.json
            ├── sub-20712_ses-12367_task-restbold_run-1_space-
MNI152NLin6Asym_seg-HCP_stat-coverage_bold.tsv
            ├── sub-20712_ses-12367_task-restbold_run-1_space-
MNI152NLin6Asym_seg-HCP_stat-mean_timeseries.json
            ├── sub-20712_ses-12367_task-restbold_run-1_space-
MNI152NLin6Asym_seg-HCP_stat-mean_timeseries.tsv
            ├── sub-20712_ses-12367_task-restbold_run-1_space-
MNI152NLin6Asym_seg-HCP_stat-pearsoncorrelation_relmat.json
            ├── sub-20712_ses-12367_task-restbold_run-1_space-
MNI152NLin6Asym_seg-HCP_stat-pearsoncorrelation_relmat.tsv
            ├── sub-20712_ses-12367_task-restbold_run-1_space-
MNI152NLin6Asym_seg-HCP_stat-reho_bold.json
            ├── sub-20712_ses-12367_task-restbold_run-1_space-
MNI152NLin6Asym_seg-HCP_stat-reho_bold.tsv
            ├── sub-20712_ses-12367_task-restbold_run-1_space-
MNI152NLin6Asym_seg-Tian_stat-alff_bold.json
            ├── sub-20712_ses-12367_task-restbold_run-1_space-
MNI152NLin6Asym_seg-Tian_stat-alff_bold.tsv
            ├── sub-20712_ses-12367_task-restbold_run-1_space-
MNI152NLin6Asym_seg-Tian_stat-coverage_bold.json
```

```
            ├── sub-20712_ses-12367_task-restbold_run-1_space-
MNI152NLin6Asym_seg-Tian_stat-coverage_bold.tsv
            ├── sub-20712_ses-12367_task-restbold_run-1_space-
MNI152NLin6Asym_seg-Tian_stat-mean_timeseries.json
            ├── sub-20712_ses-12367_task-restbold_run-1_space-
MNI152NLin6Asym_seg-Tian_stat-mean_timeseries.tsv
            ├── sub-20712_ses-12367_task-restbold_run-1_space-
MNI152NLin6Asym_seg-Tian_stat-pearsoncorrelation_relmat.json
            ├── sub-20712_ses-12367_task-restbold_run-1_space-
MNI152NLin6Asym_seg-Tian_stat-pearsoncorrelation_relmat.tsv
            ├── sub-20712_ses-12367_task-restbold_run-1_space-
MNI152NLin6Asym_seg-Tian_stat-reho_bold.json
            ├── sub-20712_ses-12367_task-restbold_run-1_space-
MNI152NLin6Asym_seg-Tian_stat-reho_bold.tsv
```

Supplemental Figure 3: Example outputs of the XCP-D walkthrough for one run.

NMIND Coding Standards Checklist

Documentation

Bronze: 9/9

- √ Landing page (e.g., GitHub README, website) provides a link to documentation and brief description of what program does
- √ Documentation is up to date with version of software
- √ Typical intended usage is described
- √ An example of its usage is shown
- √ Document functions intended to be used by users (i.e., public function docstring / help coverage ≥ 10%)
- √ Description of required input parameters for user-facing functions with reasonable description of inputs (i.e., "NIfTI of brain mask in MNI" vs. "An image file")
- √ Description of output(s)
- √ User installation instructions available
- √ Dependencies listed (i.e., external and within-language requirements)

Silver: 7/7

- √ All items from bronze tier
- √ Background/significance of program
- √ One or more tutorial to showcase the multiple of the program's usages (i.e., if program has multiple usages)
- √ Any alternative usage that is advertised is thoroughly documented
- √ Thorough description of required and optional input parameters
- √ Document public functions (i.e., public function docstring / help coverage ≥ 20%)
- √ A statement of supported operating systems / environments (i.e., could be a container recipe)

Gold: 8/8

- √ All items from bronze tier
- √ All items from silver tier
- √ Continuous integration badges in README for build status
- √ Continuous integration badges in README for tests passing
- √ Continuous integration badges in README for coverage
- √ Document functions, classes, modules, etc. (i.e., public + private docstring / help coverage ≥ 40%)
- √ Has a documented style guide
- √ Maintenance status is documented (e.g., expected turnaround time on pull requests, whether project is maintained)

Infrastructure

Bronze: 7/7

- √ Code is open source
- √ Package is under version control

√ Readme is present
√ License is present
√ Issues tracking is enabled (i.e., either through GitHub or external site)Digital Object
√ Identifier (DOI) points to latest version (e.g., Zenodo)
√ All documented installation instructions can be successfully followed

Silver: 4/4
√ All items from bronze tier
√ Issue template(s) available (i.e., information requested by developers)
√ Continuous integration runs tests
√ No excessive files included (i.e., unused files / cache; e.g., .gitignore)

Gold: 6/7
√ All items from bronze tier
√ All items from silver tier
√ Continuous integration builds packages
√ Continuous integration validates style
x Journal of Open Source Software submission
√ Contribution guide present
√ Code of Conduct present

<u>Testing</u>
Bronze: 2/2
√ Provide / generate / point to test data
√ Provide instructions for users to run tests that include instructions for evaluation for correct behavior

Silver: 3/3
√ All items from bronze tier
√ Some form of testing suite present
√ Test coverage > 50%

Gold: 3/4
√ All items from bronze tier
√ All items from silver tier
x Test coverage > 90%
√ Benchmarking information is provided for examples

Supplemental Figure 4: **NMIND Checklist for coding standards.**
