# Supplementary material for: XCP-D: A robust pipeline for the post-processing of fMRI data
Source: Imaging Neurosci (Camb). 2024 Aug 13;2:imag-2-00257. doi: 10.1162/imag_a_00257 (PMC12288603; doi:10.1162/imag_a_00257)
Supplement: Supplemental_Table_1 [file supplemental_table1.pdf]

**XCP-D STEP**	**TESTING**
Confound selection	Confirming that a loaded confound matrix has the right shape.
Removing dummy time	Looping through N=1-10 volumes to be dropped and confirming that the correct number of volumes have been dropped from a BOLD file and the corresponding confounds file.
Censoring	Creating a confounds file with values that should be omitted and confirming that the image file and the confounds file have had the same number of volumes dropped.
Despiking	Confirming that the maximum value of the voxel-wise data has decreased, and the minimum value of the voxel-wise data has increased after despiking.
Confound regression	Confirming simulated signals are retained in an image while simulated confounds are removed post denoising.
Interpolation	Confirming that volumes at the beginning and end of a simulated dataset are replaced with the nearest non-outlier volumes post the workflow.
Filtering	Confirming simulated signals are retained in an image post denoising.

Functional timeseries and connectivity matrices	Confirming that the correlation coefficient of a parcellated timeseries is the same as the connectivity matrix produced. (Parcellations were also performed manually in a separate Python notebook and compared to results from XCP-D.)
ReHo	Adding artificial noise to an image and confirming that the mean ReHo value decreases.
ALFF	Computing the FFT of a BOLD file, adding to the amplitude of its lower frequencies and confirming the ALFF increases.
Residual BOLD and resting-state derivatives smoothing	Confirming that smoothness has increased after the module - via AFNI for NIfTIs and via Connectome Workbench for CIFTIs.
Quality control	Visually inspecting the quality check reports.

Suppplemental Table 1: Continuous integration tests for different XCP-D stages.
